# Dynamically blocking leakage current in molecular tunneling junctions†

**DOI:** 10.1039/d4sc02829e

**Published:** 2024-06-27

**Authors:** Yu Xie, Shengzhe Qiu, Qianqian Guo, Chengtai Li, Ningyue Chen, Ziming Zhou, Zhenyu Yang, Zhou Cao, Tao Wang, Wei Du, Lejia Wang, Yuan Li

**Affiliations:** a Key Laboratory of Organic Optoelectronics and Molecular Engineering, Department of Chemistry, Tsinghua University Beijing 100084 P. R. China yuanli_thu@tsinghua.edu.cn; b Institute of Functional Nano & Soft Materials (FUNSOM), Soochow University Suzhou Jiangsu 215123 P. R. China wangtao2019@suda.edu.cn duwei2021@suda.edu.cn; c School of Materials and Chemical Engineering, Ningbo University of Technology Ningbo Zhejiang 315211 P. R. China wanglejia@nbut.edu.cn

## Abstract

Molecular tunneling junctions based on self-assembled monolayers (SAMs) have demonstrated rectifying characteristics at the nanoscale that can hardly be achieved using traditional approaches. However, defects in SAMs result in high leakage when applying bias. The poor performance of molecular diodes compared to silicon or thin-film devices limits their further development. In this study, we show that incorporating “mixed backbones” with flexible-rigid structures into molecular junctions can dynamically block tunneling currents, which is difficult to realize using non-molecular technology. Our idea is achieved by the interaction between interfacial dipole moments and electric field, triggering structured packing in SAMs. Efficient blocking of leakage by more than an order of magnitude leads to a significant enhancement of the rectification ratio to the initial value. The rearrangement of supramolecular structures has also been verified through electrochemistry and electroluminescence measurements. Moreover, the enhanced rectification is extended to various challenging environments, including endurance measurements, bending of electrodes, and rough electrodes, thus demonstrating the feasibility of the dynamic behavior of molecules for practical electronic applications.

## Abstract 2

Incorporating mixed flexible-rigid backbones into molecular junctions can dynamically block tunneling currents and enhance rectification.

## Graphical abstract

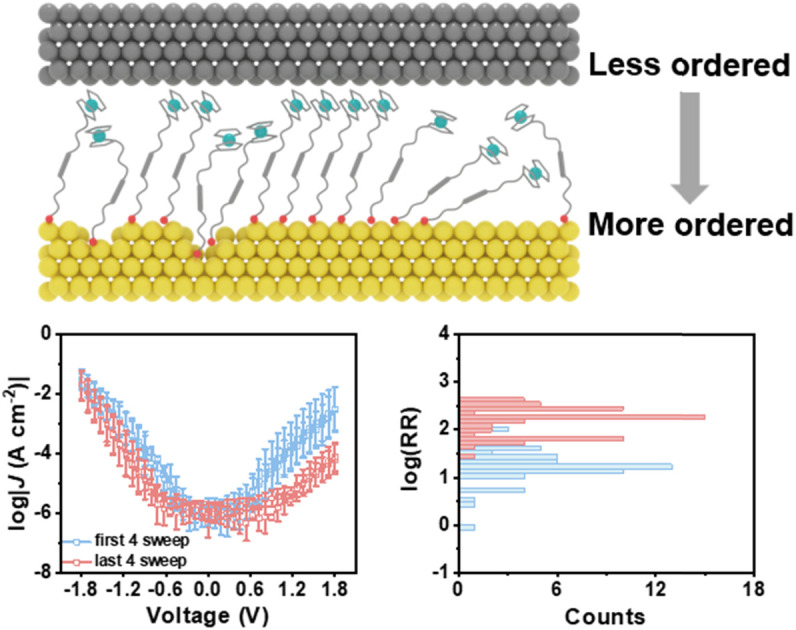

## Introduction

The ever-increasing demand for smaller, softer, and greener electronics has sparked a necessity for integrating molecules into electronics through nanofabrication.^1–3^ To tackle this challenge, the field of molecular electronics has emerged, utilizing self-assembled monolayers (SAMs), Langmuir–Blodgett films, or single molecules as the fundamental building blocks for functional electronics, such as resistors, capacitors, diodes, switches, and transistors.^4–10^ Molecular diodes, based on the unsymmetric accessibility of molecular orbitals (spatial or/and energetical) at different directions of applied bias, are the essential components among these elements. SAMs-based diodes are reliable platforms to study current rectification at the nanoscale, in which the supramolecular and electronic structure of SAMs are crucial.^11–15^ The magnitude of rectification is the most important parameter for evaluating molecular diodes. Most molecular diodes have a disappointing rectification ratio lower than 10^2^, which is attributed to large leakage derived from the supramolecular defects in SAMs.^16,17^ Previous research shows that molecular diodes with a high rectification ratio (*i.e.* small leakage) highly depend on ordered supramolecular structures of SAMs. Thus, comprehensive investigations have been conducted to systematically elucidate the optimal conditions for forming more ordered, rigid, and densely packed monolayers, including an ultra-template bottom electrode,^16^ pure molecular precursors,^18^ orbital coupling strength with an electrode,^17,19,20^ and intermolecular interactions in SAMs.^21–24^ Indeed, super-ordered SAMs can efficiently block leakage of tunneling current, but unexpected diode failures still arise in ordered SAMs owing to the charge scattering at the interfaces generating Joule heating.^25^ Differing from silicon materials, molecules mainly made from carbons are dynamic and easy to trigger chemistry or deformation that can be used to control the packing of SAMs *in situ*. The continuous molecular conformational changes under an electric field can present unpredictable dynamic behaviors in the molecular junctions, such as blinking of plasmon excitation, electrostatic contact with electrodes, and dihedral rotations.^19,26–29^ Here, we took advantage of the dynamic behavior of molecules and developed a general method to block the leakage to recuse failed molecular diodes to give high performance. We designed a series of molecules with “mixed backbones” comprising flexible linear alkane and rigid aromatic backbones. These molecules could gradually diminish leakage when subjected to an external electric field. We assume that SAMs can dynamically reach more ordered structures owing to the interaction between dipole moments and electric fields.

## Results and discussion

### Electrical response of SAMs

**Fig. 1:**
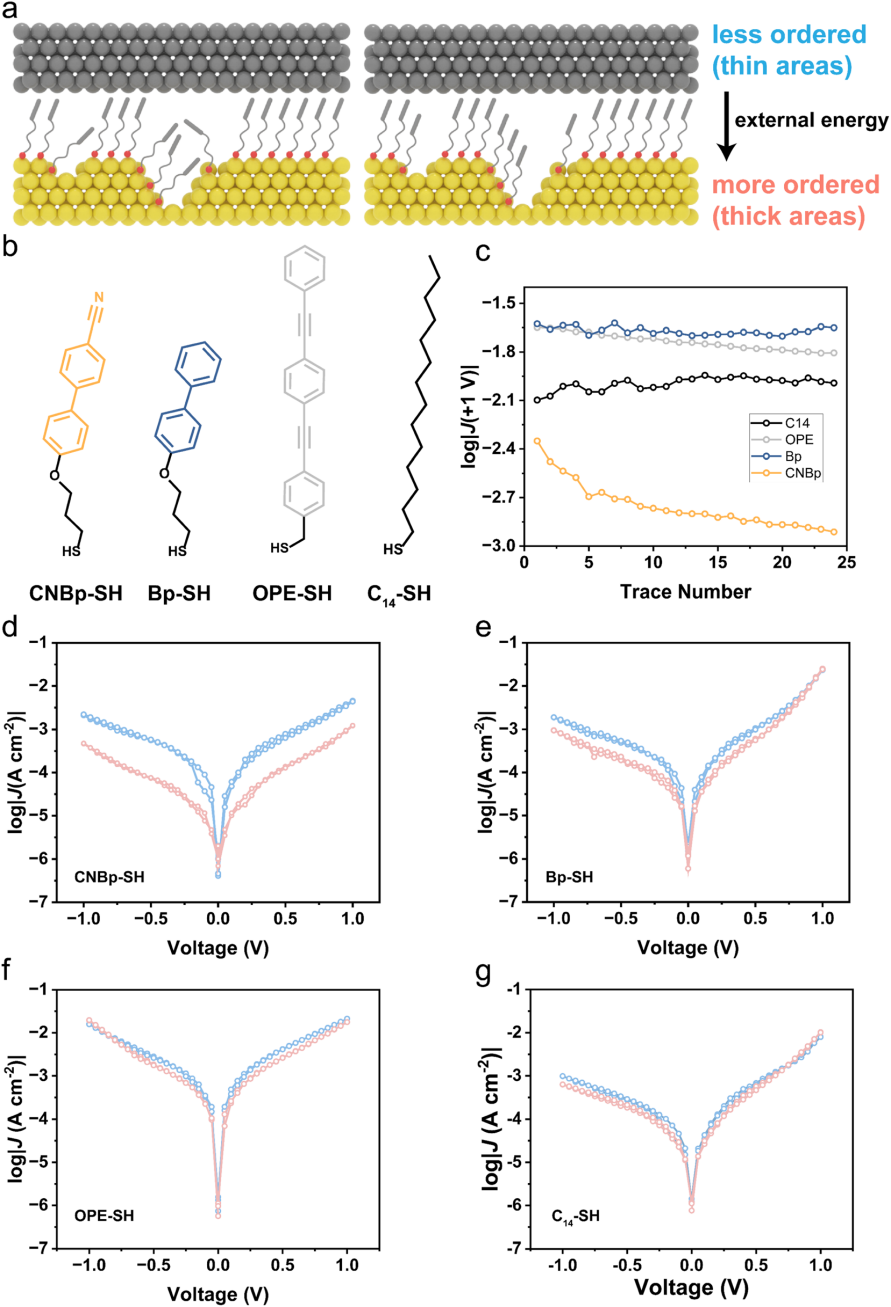
(a) Schematic illustration of Au^TS^-SAM//Ga_2_O_3_/EGaIn molecular junctions. (b) Chemical structures of the thiol precursors. (c) Plots of tunneling current log|*J*(+1.0 V)| *versus* sweep number for the molecular junctions. (d–g) log|*J*(*V*)| plots for the molecular junctions at the first sweep and last sweep.

SAMs inherently contain disordered domains, such as back-bending conformation, step-edges, and pinholes (Fig. 1a).^30–32^ We used the Simmons equation to simply correlate tunneling current, *J*, with the degree of order in SAMs:

1 *J* = *J*_0_ e^−*βd*^

Tunneling currents are highly sensitive to the tunneling barrier length, *d*. Ordered structures of molecules yield thicker SAMs (*d*), meaning longer tunneling distances and lower tunneling currents.^33–35^ Thus, it is a reflection of a more ordered structure in SAMs if the current across the junction diminishes owing to the exponential decay over the longer tunneling distance.

As illustrated in Fig. 1a, molecules comprising biphenyl (–Bp–) are introduced into SAMs and we use eutectic gallium indium (EGaIn) liquid metal as the top electrode to form a non-invasive contact with SAMs and perform *J*(*V*) measurements.^36^ According to the chemical structures of thiol precursors, we classify the CNBp-SH (4′-(3-mercaptopropoxy)-[1,1′-biphenyl]-4-carbonitrile) and Bp-SH (3-([1,1′-biphenyl]-4-yloxy)propane-1-thiol) as “mixed backbone”, C14-SH (tetradecane-1-thiol) as “flexible backbone” and OPE-SH ((4-((4-(phenylethynyl)phenyl)ethynyl)phenyl)methanethiol) as “rigid backbone” (Fig. 1b). We sweep external bias on the junction following 0 → −1.0 → +1.0 → 0 V and measure the corresponding tunneling current density *J*. To elucidate the trends of tunneling currents, we plot *J*(+1 V) *versus* sweep number. A clear current decay for Au^TS^–S–BpCN//Ga_2_O_3_/EGaIn, as it appears from Fig. 1c, signals the gradual increase of tunneling distance in the junction when sweeping. In contrast, the current decay is absent for other junctions. To show the difference between the first and last sweep, we plot *J*(*V*) and observe a distinct decrease in tunneling currents for Au^TS^–S–BpCN//Ga_2_O_3_/EGaIn (Fig. 1d–g).

Based on our measurements, we conclude that the molecular junctions can gradually block tunneling currents only if molecules have (i) a “mixed backbone” and (ii) polarized substituents. The schematic illustrations of the structural transformation of SAMs are presented in Fig. 1a. This observation implies that the polarized substituents, such as nitrile functionality, have a propensity to align with the electric field, effectively blocking the leakage currents in the junctions. We infer that the electrostatic energy arising from the interaction of interfacial dipoles and external electric field can induce rearrangements in the supramolecular structures and longer tunneling distances for charge transport. When an external electric field (*E⃑*) is correctly applied, aligning the vertical component of the molecular dipole moment with the direction of *E⃑* may lead to a “pull-up” effect on the molecules. This encourages a quasi-stable supramolecular structure with a higher degree of order (Fig. 1a) and consequently yields thicker SAMs. We hypothesize that the conjugated OPE molecules in SAMs lack a degree of freedom, while the flexible C14 are densely packed, requiring higher energy for conformational change. Thus, introducing mixed backbones and polar groups is crucial for dynamically diminishing tunneling currents.

### Molecular diodes: mechanism and supramolecular structures

**Fig. 2:**
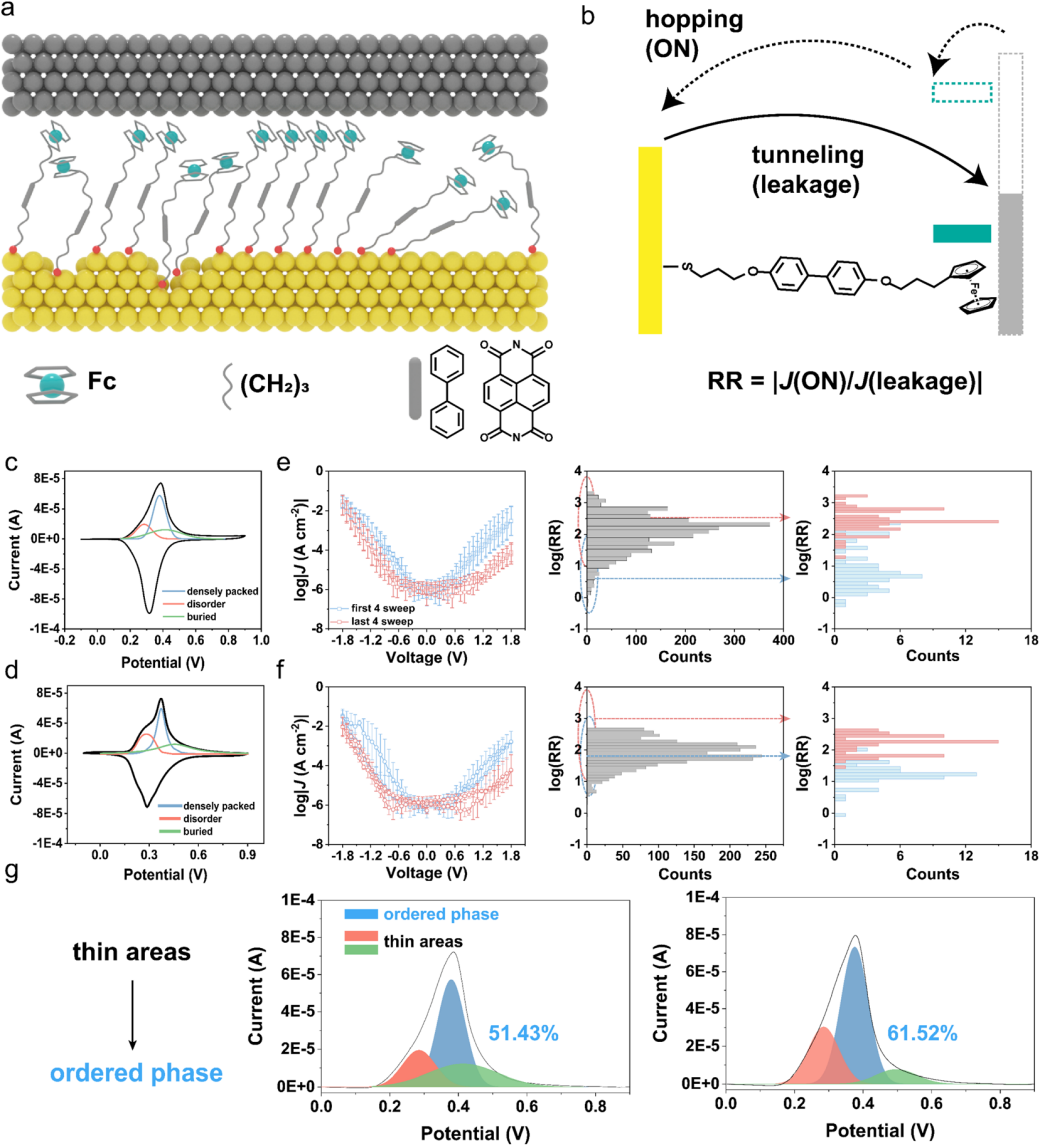
(a) Schematic illustration of SAM-based molecular diodes. Thiols are composed of mixed backbones with an Fc functional group. (b) Energy level diagram and charge transport of Fc-based diodes. CV measurements and deconvolution of the anodic peaks of (c) Au–S–B1–Fc and (d) Au–S–B2–Fc. Demonstration of the blocking of leakage and corresponding rectification ratio enhancement for (e) Au–S–B1–Fc and (f) Au–S–B2–Fc. Average log|*J*(*V*)| plots of molecular junctions in the first four sweeps and last four sweeps. The distribution of log(RR) for all 150 sweeps and corresponding log (RR) in the first four sweeps and last four sweeps. (g) CV measurements on SAMs are carried out to reflect the transition from thin areas (disordered, buried domains) to ordered phase. Peak split shows the ratio of ordered phase in SAMs.

We observe that as for trivial molecular junctions with mixed backbones, ordered supramolecular structures can diminish tunneling currents in both directions of bias (Fig. 1d). Similarly, leakage of molecular didoes is highly dependent on supramolecular structures. Thus, it is promising to incorporate a “mixed backbone” into molecular diodes to diminish leakage and enhance rectification (Fig. 2a).

We use ferrocene (Fc), a commonly employed functional moiety, to generate strong current rectification with a well-defined mechanism of rectification at the molecular scale.^37–40^ This well-established mechanism of rectification with Fc-based molecular junctions is schematically illustrated in Fig. 2b: under negative bias at the top electrode, the HOMO of Fc falls within the energy window and participates in the charge transport, which allows current to flow more efficiently through the junction. Under reverse bias, the HOMO falls below both Fermi levels, causing the diodes to be in the OFF state, blocking the current and leaving only a small leakage (denoted as *J*(+*V*), *i.e.* current density at positive bias in this work).^41^ From our previous studies, we found that the presence of disordered domains in SAMs significantly increases the leakage currents.^16,42^ Especially, when molecules composed of Fc form SAMs on Au^TS^ surfaces (the surface contains smaller flat grains compared to Ag^TS^ and Pt^TS^ surfaces) only showing a disappointing rectification ratio (RR) ∼ 10 and remain consistent even under the influence of a large applied electric field (approximately 1–10 GV m^−1^).^39,43,44^

Various parameters can reflect failed molecular diodes directly or indirectly, including EGaIn junctions (leakage), photoelectron spectroscopy (tilt angle, thickness, and binding energy), and cyclic voltammograms (redox peaks). Generally, high-performance Fc diodes with small leakage are dependent on highly ordered SAMs. Unfortunately, only a few molecules match the standard and yield a high-performance diode (RR > 10^2^). In consideration of the strong correlation between leakage and supramolecular structures of SAMs, the supramolecular structures, together with the leakage, were determined once the SAM had been formed. If the supramolecular structures of SAMs become more ordered by degrees, it could decouple the leakage and initial structures of SAMs. From the above-mentioned experiments, introducing mixed backbones can induce ordered SAMs with assistance from an external electric field, which may provide a general template to dynamically yield high-performance molecular diodes.

To fully investigate the effect of mixed backbones in molecular diodes, we fabricate SAMs derived from Fc–B–SH (B represents the backbone of Fc thiols), which incorporates biphenyl (B1) and naphthalene diimides (B2) into SAMs. The schematic structure of the molecular diode is depicted in Fig. 2a. In our study, we form Fc-based SAMs on a gold substrate and perform XPS to determine the species of sulfur components (Fig. S11†). According to the fitting of the S_2p_ doublet, whose binding energy is ∼162.0 eV, we verify that the thiols covalently form Au–S bonds on the gold substrate rather than physisorption. UPS is used to determine the HOMO of the molecules in SAMs (Fig. S12†). The HOMO is lower than −5.0 eV for Fc molecules with different backbones, which means rectifying mechanism (Fig. 2b) is proper for these Fc molecules.^18^ We characterize the structures of SAMs with cyclic voltammetry (CV). The CV of SAMs shows the electrochemical behavior of Fc termini in relation to their supramolecular structures. Surface coverage (*Γ*) can be determined following equations reported in previous research, where *Q*_tot_ is total charge when redox, *F* is Faraday's constant and *A* is charging area.^21^

2 *Γ* = *Q*_tot_/*nFA*

SAMs formed with Fc–B1–SH and Fc–B2–SH have surface coverage *Γ* = 3.79 × 10^−10^ and 2.97 × 10^−10^ mol cm^−2^, respectively, slightly lower than the value derived from ferrocene alkanethiols. A mismatch of the size between the backbone and Fc unit and roughness of the substrates results in defects in SAMs. Peak deconvolution of the anodic peaks reflects three different types of structures in SAMs:^45^ densely packed, disordered, and buried domains (Fig. 2c and d). The ratio of densely packed domains is 51.43% and 40.51% for Au^TS^–S–B1–Fc and Au^TS^–S–B2–Fc, respectively. Disordered and buried domains form thin areas in SAMs, which induce higher leakage and lead to the molecular diodes failing.^16^

To measure the electrical characteristics of molecular diodes comprising B1 and B2, we used EGaIn top electrodes to form tunneling junctions with SAM. The rectification ratio is given by eqn (3), where we define the current density *J* (−1.8 V) flowing across the junction with “hopping” as ON current while *J* (+1.8 V) is leakage.3
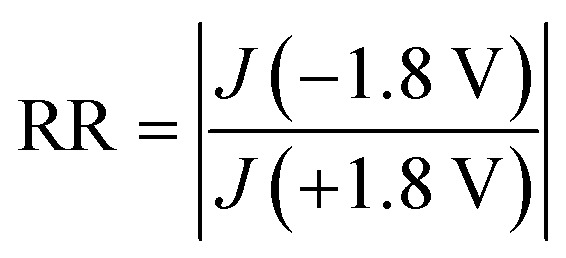


We applied 150 continuous sweeps at one junction and measured ∼20 junctions in total for statistical analysis of 3000 sweeps for one molecule. Fig. 2e and f demonstrates the average log|*J*(*V*)| sweeps of the first four sweeps (blue) and last four sweeps (pink). We observe that the high leakage results in the failed molecular diodes (blue, RR < 10), in line with defects in SAMs demonstrated through surface characterization. Nonetheless, after 150 sweeps, the leakage of the molecular diodes decreases remarkably, resulting in a higher rectification ratio (pink, RR > 100). We plot the histograms of log(RR) collected from 150 sweeps of Au^TS^–S–B–Fc//Ga_2_O_3_/EGaIn junctions, which show a broad distribution. The noteworthy discreteness in the log(RR) distributions for the first four and last four sweeps demonstrates a significant enhancement of rectification for molecular diodes with mixed backbones.

According to eqn (1), dynamically blocking leakage suggests a longer tunneling distance (*d*), that is, yielding thicker SAMs in the tunneling junction. Thin areas (disordered, buried domains) inducing leakage in molecular diodes can gradually convert to densely packed domains under an electric field. To validate the more ordered SAMs under an electric field, we biased the bottom electrode to apply an electric field on Au^TS^–S–B1–Fc in aqueous solution and analyze its electrochemical behavior. To avoid cleavage of the Au–S bond and redox reaction of Fc units, we used 1.0 mol L^−1^ Na_2_SO_4_ as the electrolyte and applied bias from +0.3 to −1.3 V, followed by removing Na_2_SO_4_ solution and carrying out CV measurements in HClO_4_. The peak split shows that the portion of ordered domains increases to 61.52% from 51.43% after applying an electric field on SAMs in aqueous solution, suggesting the formation of more ordered structures under an electric field (Fig. 2g).

### Blocking leakage in failed molecular diodes

**Fig. 3:**
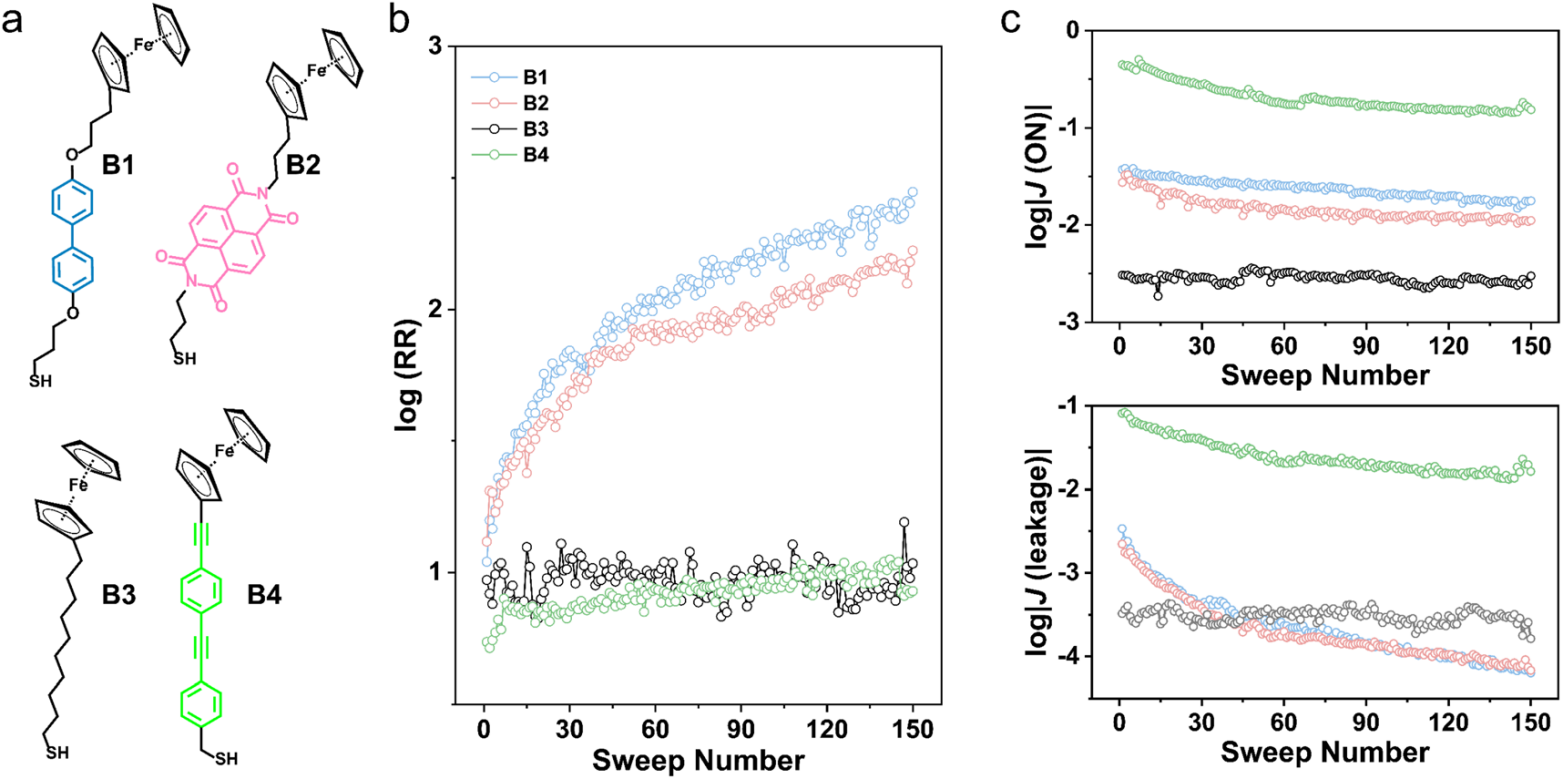
(a) Chemical structures of Fc-based thiols. Plots of average trend of (b) log(RR) and (c) corresponding log|*J*(*V*)| over 150 sweeps.

For comparison, we introduce molecular diodes containing only flexible (B3) or only rigid (B4) backbones (Fig. 3a). Fig. 3b demonstrates the trend of log(RR) in 150 continuous sweeps (plots with error bars are available in ESI Section 5†). Table 1 compiles the mean and standard deviations of the initial and ultimate log(RR). The initial RR is about 10 for all the molecular diodes. However, we observe an increase of RR by more than one order of magnitude with molecules B1 and B2, which is absent for molecules B3 and B4. This observation manifests that the addition of mixed backbones can efficiently improve the performance of failed molecular diodes. Furthermore, the corresponding log|*J*(*V*)| reveals that the enhancement of log(RR) is exclusively attributed to the decline of leakage currents (Fig. 3c). The corresponding values of ON currents (log|*J*(ON)|) decrease slightly in 150 sweeps. The leakage, however, decreases more significantly for B1 and B2, in comparison with B3 and B4. We investigate the attenuation of ON currents and block of leakage independently, which rely on electronic structures and supramolecular structures respectively of molecular diodes.

**Table 1:** Initial and ultimate log(RR) and corresponding current density

Molecule	log(RR)			log|*J*(ON)|		log|*J*(leakage)|	
	Initial (*σ*)	Ultimate (*σ*)	Enhancement (%)	Initial (*σ*)	Ultimate (*σ*)	Initial (*σ*)	Ultimate (*σ*)
Fc–B1–SH	1.04(0.88)	2.45(0.31)	135.58	−1.43(0.37)	−1.75(0.42)	−2.47(0.69)	−4.20(0.37)
Fc–B2–SH	1.12(0.43)	2.22(0.26)	98.21	−1.56(0.36)	−1.95(0.42)	−2.65(0.49)	−4.17(0.68)
Fc–B3–SH	0.97(0.42)	1.03(0.43)	6.19	−2.52(0.48)	−2.52(0.77)	−3.49(0.67)	−3.78(0.86)
Fc–B4–SH	0.74(0.40)	0.93(0.46)	25.7	−0.35(0.87)	−0.81(0.93)	−1.09(0.92)	−1.79(1.24)

### Attenuation of ON currents when applying an electric field

As mentioned above, ON currents of molecular diodes rely mostly on a proper energy alignment between molecular orbitals and the Fermi level of the electrodes. The continuous application of such a large electric field at molecular junctions may change the properties of the SAM and SAM//EGaIn interface. Thus, we infer that the attenuation is attributed to the oxidation of the EGaIn tip and can be verified by the transition voltage spectrum. The growing Ga_2_O_3_ layer can form a Schottky contact between the top electrode and SAMs and increase the tunneling barrier length, in turn decreasing the current.^46^

**Fig. 4:**
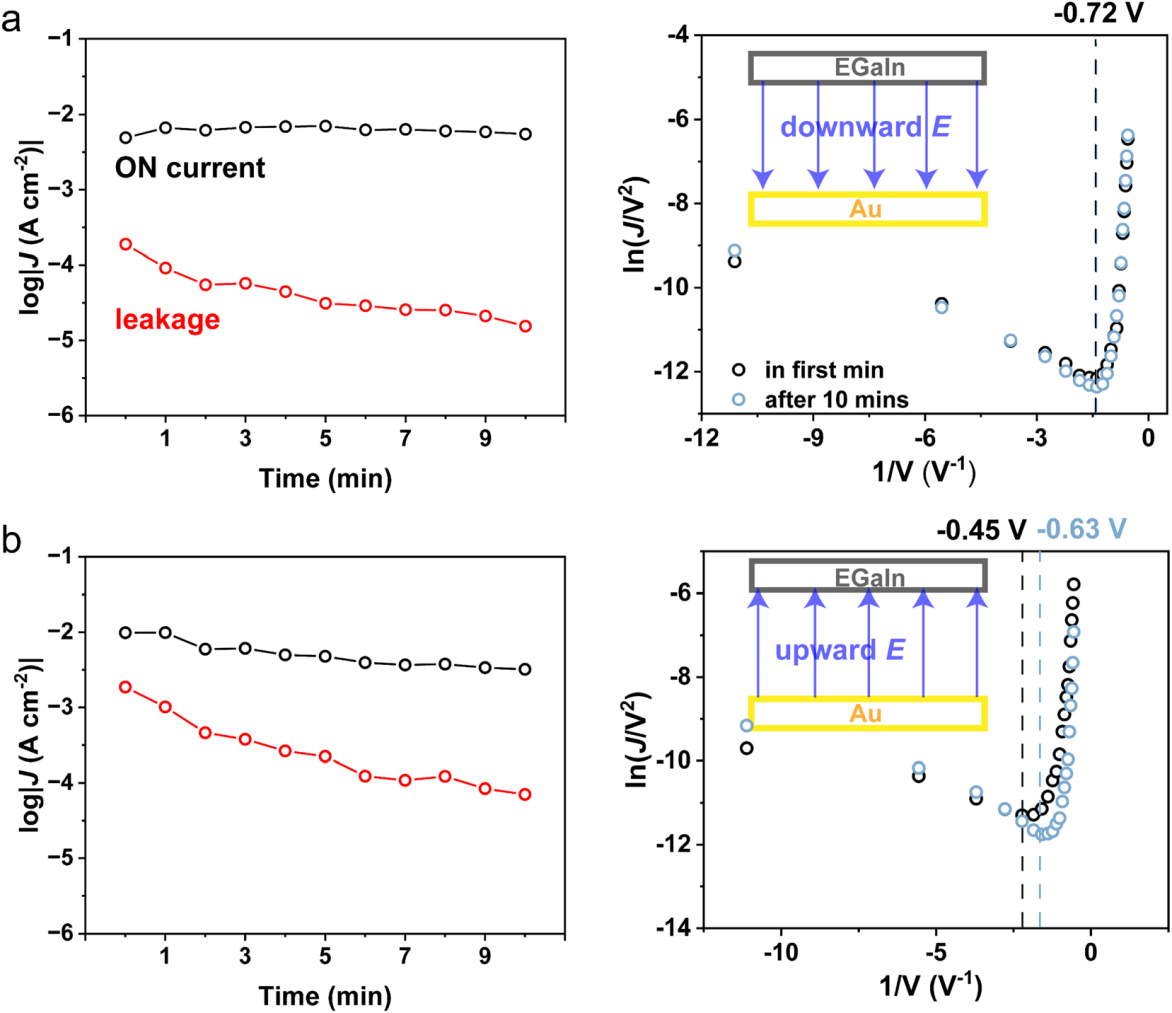
Plots of log|*J*(±1.8 V)| measured every 1 min when applying (a) downward and (b) upward *E⃑* on Au–S–B1–Fc//Ga_2_O_3_/EGaIn junctions and corresponding transition voltage spectrum. The dashed lines correspond to the transition voltage, reflecting the tunneling barrier height in the SAMs//Ga_2_O_3_ interface.

To elucidate the effect of the electric field on the electronic structure of SAMs, we applied a constant bias of +1.8 V or −1.8 V on the junction for 10 minutes and performed *J*(*V*) measurements every 1 minute to study the trend of log|*J*(±1.8 V)|. Fig. 4 shows that the leakage can be blocked under downward and upward *E⃑*. However, under upward *E⃑*, there is a slight attenuation of the ON current, log|*J*(−1.8 V)|, which is absent when applying downward *E⃑*. We assume it originates from the oxidation of the EGaIn tip after a long time scanning under ambient conditions. The transition voltage remains the same when applying downward *E⃑* (Fig. 4a).^47^ In contrast, the absolute value of the transition voltage increases from −0.45 V to −0.63 V after applying upward *E⃑* for 10 min, representing a slight increase in the height of the tunneling barrier (Fig. 4b). Therefore, the attenuation of ON currents originates from an upward electric field. According to the constant tunneling barrier height, we can safely exclude the effect of the Ga_2_O_3_ layer in the blocking of leakage when applying downward *E⃑*.

### *In situ* visualization of ordered SAMs in molecular diodes

**Fig. 5:**
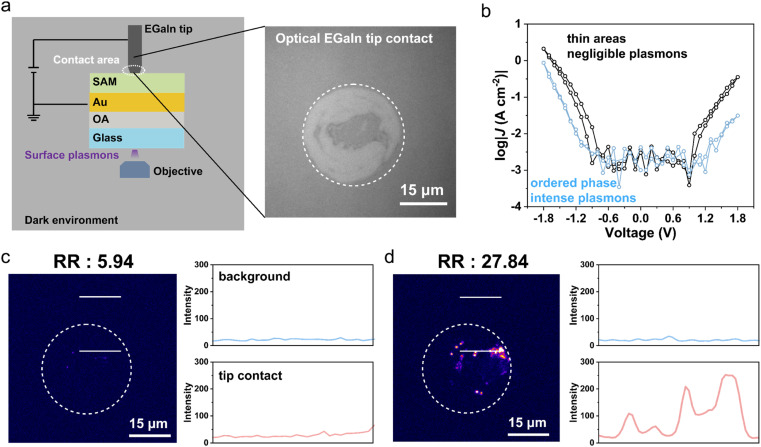
(a) Schematic illustration of home-built inverted wide-field microscope to conduct electrical and optical measurements simultaneously. Footprint of EGaIn tip with SAMs under bright field inside the dashed circle. Outside area is regarded as background area. (b) *J*(*V*) sweeps of Au–S–B1–Fc//Ga_2_O_3_/EGaIn junction before and after applying downward *E⃑*. (c) Image of plasmons excited in the molecular junction with conformational defects, whose current rectification ratio is 5.94. Cross-section intensity profiles of plasmons are plotted along the white lines in the background and tip contact areas. (d) Image of plasmons excited in a more ordered phase with much brighter electroluminescent spots, whose current rectification ratio is 27.84. Line scans show the electroluminescent intensity in the background area and EGaIn footprint area.

Previous studies have shown that the tunneling electrons can excite surface plasmons. The number of electroluminescent spots is directly proportional to the number of molecules that conduct current inside SAMs.^19^ Ordered SAMs can produce more upright molecules to contact the top electrode serving as conducting channels. Electroluminescent measurements provide evidence of the correlation between the applied electrical field and number of conducting molecules, as indicated by electroluminescent spots. Fig. 5a shows a schematic illustration of the home-built testbeds for conducting electrical and optical measurements simultaneously. The footprint of the EGaIn tip with SAMs is captured through the inverted objective circled in dash line.^48^ Light emission images were acquired at −1.8 V with an acquisition time of 30 seconds. Initially, for molecular diodes with conformational defects, high leakage currents and a lower rectification ratio are observed (black curve in Fig. 5b), alongside negligible electroluminescent spots and emission intensity (Fig. 5c). However, after applying downward *E⃑* for 1 minute, the number of light-emitting spots increases (Fig. 5d), along with the blocking of leakage and a higher rectification ratio (blue curve in Fig. 5b). The augmentation of conducting molecules is indicative of the dynamic rearrangement of molecules in response to the electric field. Leakage is more sensitive to the supramolecular structures, showing a more pronounced decrease than *J*(−1.8 V). Therefore, we conclude that, after applying downward *E⃑*, more molecules are forced to adopt upright conformation and become involved with charge transport, thereby blocking leakage more efficiently.

From the preceding section, tunneling junctions with mixed backbones can gradually block tunneling currents through transition into more ordered structures. As for flexible molecules with an alkyl chain, the strong chain–chain entanglement increases the total packing energy of SAMs, which requires high energy for conformational transformation. As for rigid backbones, the lack of degrees of freedom of the molecules is unfavorable for conformational change. It is worth noting that dipole moments of SAMs are indispensable for the dynamic blocking of leakage current. The interfacial dipole moments of molecules tend to align perpendicularly with the electric field, resulting in a “pulling effect” on the molecules to yield thicker SAMs. Thus, molecular diodes can block leakage current gradually under an electric field. Under upward *E⃑*, the Fc units are oxidized to form Fc^+^. Molecules are inclined to rearrange to minimize Fc^+^–Fc^+^ electrostatic repulsion, stabilizing the overall energy of junctions. This rearrangement forces the molecules to be more “upright”, increasing the tunneling barrier length.^19,21,49^ Besides, Fc^+^ can rotate to approach the negatively charged top electrode by electrostatic attraction, further increasing the tunneling distance as well.^19^ In summary, from both scenarios, interfacial dipole moments interact with the electric field, leading to the rearrangement of molecules and formation of thicker films in SAMs. Leakage currents, which are highly sensitive to the supramolecular structures of SAMs, are dynamically blocked under the influence of an electric field, thereby effectively enhancing the rectification ratio.

### Application of rectification enhancement

To thoroughly assess the rectification enhancement and demonstrate that the “mixed backbone” can be used as a general method for the structural design of molecular diodes, we present three distinct experiments that address different scenarios for the application of molecular diodes:

#### (i) Endurance test for enhanced rectification

**Fig. 6:**
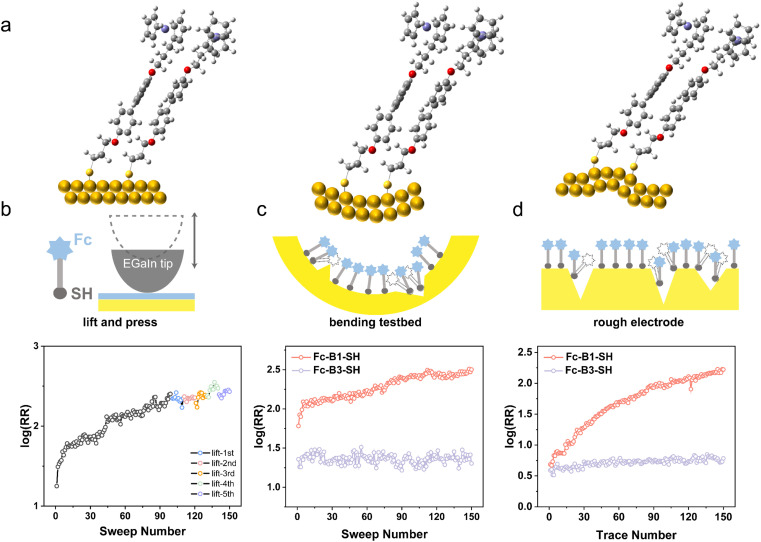
(a) Simple CPK models of Au–S–B1–Fc dimer under different substrates. (b) Successful enhancement of rectification on molecular diodes of Au^TS^–S–B1–Fc//Ga_2_O_3_/EGaIn in 100 sweeps (black scatters), followed by breaking and forming junctions at the same place five times (colored scatters). (c) Enhancement of log(RR) on bending testbed with bending radius of 17.0 mm. (d) Enhancement of log(RR) can be observed on rough electrodes Au^DE^ (RMS = 1.0 nm) as well.

To determine whether the conformational transformation of SAMs induces a persistent RR enhancement after the removal of *E⃑*, we initially performed 100 sweeps (represented by black spots in Fig. 6b), and subsequently, we lifted the tip to break the junction. We then pressed to re-form the junction in the same location and measured 10 *J*(*V*) sweeps from the newly formed junctions (represented by colored spots in Fig. 6b). The rectification ratio of these newly formed junctions was found to be similar to the ultimate value achieved after the initial 100 sweeps. This compelling result suggests that the more ordered structures of SAMs are persistent, leading to a robust RR enhancement.

#### (ii) Flexible device application

To explore the possibility of using molecular diodes in flexible electronic applications, we utilized a home-built bending testbed (Fig. S10†). In this experiment, we employed PET (polyethylene terephthalate)-supported template-stripped gold electrodes, which were bent in a mechanically controlled device with a bending radius of 17.0 mm, as the bottom electrode. By comparing the performance of flexible Au–S–B3–Fc//Ga_2_O_3_/EGaIn junctions with that of Au–S–B1–Fc//Ga_2_O_3_/EGaIn molecular diodes, we observed an RR enhancement of approximately five times for the latter (Fig. 6c). This outcome indicates that molecular diodes exhibit promising characteristics for a wide range of flexible and durable electronic applications.

#### (iii) Feasibility on rough surface

While it is well known that ultra-flat bottom electrodes can efficiently block leakage currents in molecular diodes, direct-deposition bottom electrodes (Au^DE^), which possess rougher surfaces compared to template-stripped bottom electrodes, often struggle to effectively block leakage currents. Consequently, the rectification ratio of diodes constructed on such rough electrodes typically remains close to unity.^16^ In contrast, molecular diodes featuring flexible-rigid structures demonstrate more efficient blocking of leakage currents under the influence of an electric field. This feature is associated with a significant enhancement in the rectification ratio, as illustrated in Fig. 6d.

## Conclusion

In summary, we demonstrate that the introduction of mixed backbones into SAMs can dynamically block the tunneling currents within molecular junctions. Thin areas in SAMs induce large tunneling currents of molecular junctions. This effect arises from the “pull-up” conformation of molecules under an applied electric field, which is maintained after the electric field is removed. Our CV measurements and *in situ* electrical field-driven plasmonic excitation measurements provide direct evidence of the rearrangement of SAM structures. CV measurements manifest the increasing portion of ordered phase in SAMs after applying electric field in aqueous solution. The increasing number and intensity of electroluminescent spots indicates conformational changes in the SAM structure. Leakage of failed molecular diodes is successfully blocked when applying an electric field, together with rectification enhancement. Introducing “mixed backbones” compromises the packing energy and degrees of freedom in SAMs, making conformational changes of molecules possible. The blocking of leakage is achieved through the formation of more ordered SAMs structures, facilitated by the electrostatic interaction between dipole moments and electric field.

Therefore, backbone rearrangement, leading to optimal supramolecular structures, efficiently blocks leakage currents, as we expected, and we can showcase that the incorporation of mixed backbones has extended the applicability of molecular diodes to challenging environments, such as rough surfaces and bending testbeds.

## Data availability

The data supporting this article have been included as part of the ESI.† All the NMR and MS origin data can be found in https://doi.org/10.6084/m9.figshare.26058946.v1.

## Author contributions

Y. X., L. W. and Y. L. conceptualized the work. Y. X. and S. Q. performed electrical characteristics measurements. S. Q., C. L. and Z. Z. synthesized the compounds. N. C. and Z. Y. performed characterization of SAMs. Q. G., Y. X., T. W. and W. D. performed optical experiments. Z. C. conducted AFM measurements. Y. X., L. W. and Y. L. wrote the manuscript. All authors discussed the results and commented on the manuscript.

## Conflicts of interest

The authors declare no competing interests.

## Supplementary Material

SC-015-D4SC02829E-s001
