# Supplementary material for: Dynamically blocking leakage current in molecular tunneling junctions
Source: Chem Sci. 2024 Jun 27;15(32):12721–31. doi: 10.1039/d4sc02829e (PMC11322961; doi:10.1039/d4sc02829e)
Supplement: SC-015-D4SC02829E-s001 [file SC-015-D4SC02829E-s001.pdf]

Electronic Supplementary Material (ESI) for Chemical Science.
This journal is © The Royal Society of Chemistry 2024

# Dynamically Blocking Leakage Current in Molecular Tunneling Junctions

Yu Xie[#], Shengzhe Qiu[#], Qianqian Guo, Chengtai Li, Ningyue Chen, Ziming Zhou, Zhenyu Yang, Zhou Cao, Tao Wang,[*] Wei Du,[*] Lejia Wang,[*] and Yuan Li[*]

1. **Materials and methods**

2. **Synthesis and characterization of molecules**

   2.1 **NC-Bp-O-$(CH_2)_3$-SH**

   2.2 **Bp-O-$(CH_2)_3$-SH**

   2.3 **OPE-SH**

   2.4 **Fc-$(CH_2)_3$-O-Bp-O-$(CH_2)_3$-SH**

   2.5 **Fc-$(CH_2)_3$-NDI-$(CH_2)_3$-SH**

   2.6 **Fc-OPE-SH**

   2.7 **NMR and MS spectra, CHN analysis of novel compounds**

3. **Bottom electrode fabrication and preparation of the SAMs**

4. **Electrochemistry**

5. **Fabrication and measurement of the molecular junctions**

   5.1 **Junction results under continuously changed electric field *E***

   5.2 **Light emission experiments**

   5.3 **Bending testbeds**

6. **Photoelectron spectroscopy**

## 1. Materials and Methods

All chemicals were purchased from commercial suppliers, including General-reagent®, Bidepharm and Merck, which were used without further purification. Column chromatography was conducted using chromatographic column (SEPAFLASH™ E, irregular silica, 40-63 μm, 60 Å) purchased from Santai Technologies, Inc.

**NMR spectra** were recorded on a Bruker AVANCE III 400M NMR Spectrometer.

**Mass spectra** of compounds were determined on SHIMADZU LCMS IT/TOF Mass Spectrometer: ESI total flow, 0.4 mL/min; detector voltage, 1.63 kV; nebulizing gas flow, 1.5 L/min.

**CHN analysis** was conducted on Thermo Scientific™ FlashSmart™ Elemental & Isotope Analysis.

**Atomic force microscopy (AFM)** was conducted on a Bruker Dimension Icon AFM system with ScanAsyst and the data were processed by NanoScope version 1.9.

**Thermal evaporation**: Gold pellets with purity of 99.999% were obtained from DM Material. Silicon (100, p-type) wafers were from SiBranch, with a thickness of 525 ± 25 μm with one side polished. Thermal evaporator KYKY-400 (KYKY TECHNOLOGY CO., LTD.) to deposit a layer of Au at about $9.0 \times 10^{-5}$ bar.

**Electrochemical measurements** were carried out with An Autolab PGSTAT302T equipped with NOVA 2.1.4 software.

**X-ray photoelectron spectroscopy (XPS)** and **ultraviolet photoelectron spectroscopy (UPS)** were carried out at the National Center of Electron Spectroscopy in Beijing with Thermo Scientific™ ESCALAB™ Xi. The ultra-high vacuum (UHV) chamber had a base pressure of $1.0 \times 10^{-10}$ mbar and measurements are at room temperature.

## 2. Synthesis and characterization of molecules

NC-Bp-O-$(CH_2)_3$-SH and Bp-O-$(CH_2)_3$-SH were synthesized to investigate the significance of mixed backbones in charge transport of molecular tunneling junctions. Fc-$(CH_2)_3$-O-Bp-O-$(CH_2)_3$-SH, Fc-$(CH_2)_3$-NDI-$(CH_2)_3$-SH and Fc-OPE-SH were synthesized to demonstrate the rectification enhancement in molecular didoes. The synthetic sections 1.4 and 1.5 were finished in School of Materials and Chemical Engineering, Ningbo University of Technology. The rest synthetic sections were finished in Key Laboratory of Organic Optoelectronics and Molecular Engineering, Department of Chemistry, Tsinghua University

**2.1 NC-Bp-O-$(CH_2)_3$-SH**

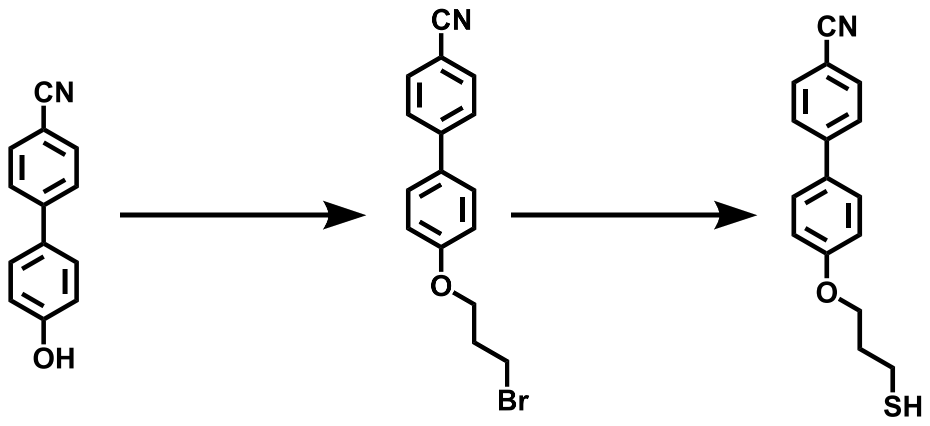


**S1** **S2**

**Synthesis of S1**: Put 195.1 mg 4'-hydroxy-[1,1'-biphenyl]-4-carbonitrile (1.0 mmol) and 1.0 g 1,3-dibromopropane (5.0 mmol) into flask, adding 10.0 mmol $K_2CO_3$ and 150.0 ml anhydrous acetone into the flask. The mixture was stirred and heated to reflux under nitrogen atmosphere for 12 hours. After filtration and solvent evaporation, the residue was purified by silica-gel column chromatography using dichloromethane/petroleum ether 1:3 as an eluent. $^1$H-NMR (400 MHz, $CDCl_3$): δ 7.75 – 7.65 (m, 4H), 7.56 (d, J = 8.8 Hz, 2H), 7.04 (d, J = 8.8 Hz, 2H), 4.19 (t, J = 5.8 Hz, 2H), 3.65 (t, J = 6.4 Hz, 2H), 2.38 (m, 2H).

**Synthesis of S2**: 157.5 mg **S1** (0.5 mmol) and 2.5 mmol thiocarbamide were dissolved in 20.0 ml of anhydrous ethanol under nitrogen atmosphere. After refluxing at 80 °C for 2 h, degassed aqueous sodium hydroxide solution was added and fluxed for 2 h. After the solution cooled to room temperature, 1.0 mol/L

HCl was added to adjust the pH < 7.0. The solid was separated and rinsed with water. The crude product was dissolved in dichloromethane and dried over $Na_2SO_4$, purified by silica-gel column chromatography using dichloromethane/petroleum ether 1:3 as an eluent. $^1$H NMR (400 MHz, $CDCl_3$) δ 7.72 – 7.61 (m, 4H), 7.53 (d, J = 8.8 Hz, 2H), 7.00 (d, J = 8.8 Hz, 2H), 4.15 (t, J = 5.9 Hz, 2H), 2.76 (dt, J = 8.1, 7.0 Hz, 2H), 2.16 – 2.08 (m, 2H). $^{13}$C NMR (126 MHz, $CDCl_3$) δ = 159.50, 145.22, 132.61, 131.67, 128.42, 127.15, 119.11, 115.12, 110.18, 65.79, 33.24, 21.22. MS (ESI) m/z : M+ calcd for 268.0796; found: 268.0819.

**2.2 Bp-O-$(CH_2)_3$-SH**

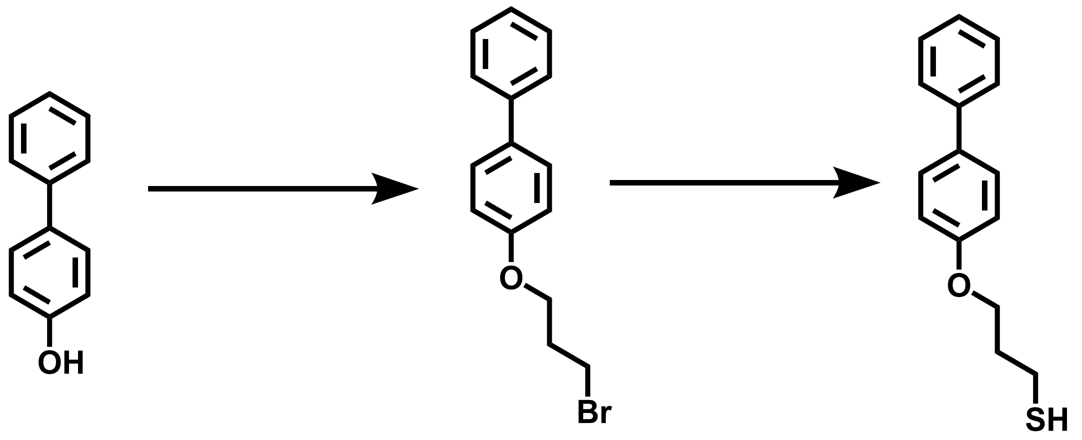


**S3** **S4**

**Synthesis of S3**: Put 170.1 mg [1,1'-biphenyl]-4-ol (1.0 mmol) and 1.0 g 1,3-dibromopropane (5 mmol) into flask, adding 10.0 mmol $K_2CO_3$ and 150 ml anhydrous acetone into the flask. The mixture was stirred and heated to reflux under nitrogen atmosphere for 12 hours. After filtration and solvent evaporation, the residue was purified by silica-gel column chromatography using dichloromethane/petroleum ether 1:5 as an eluent. $^1$H NMR (400 MHz, $CDCl_3$) δ 7.56 (m, 4H), 7.44 (dd, J = 8.5, 6.9 Hz, 2H), 7.36 – 7.30 (m, 1H), 7.01 (d, J = 8.8 Hz, 2H), 4.18 (t, J = 5.8 Hz, 2H), 3.65 (t, J = 6.5 Hz, 2H), 2.37 (m, 2H).

**Synthesis of S4**: 145.0 mg **S3** (0.5 mmol) and 2.5 mmol thiocarbamide were dissolved in 20.0 ml of anhydrous ethanol under nitrogen atmosphere. After refluxing at 80 °C for 2 h, degassed aqueous sodium hydroxide solution was added and fluxed for 2 h. After the solution cooled to room temperature, 1.0 mol/L

HCl was added to adjust the pH < 7.0. The solid was separated and rinsed with water. The crude product was dissolved in dichloromethane and dried over $Na_2SO_4$, purified by silica-gel column chromatography using dichloromethane/petroleum ether 1:10 as an eluent. $^1H$ NMR (400 MHz, $CDCl_3$) δ 7.60 – 7.53 (m, 4H), 7.44 (dd, J = 8.4, 6.9 Hz, 2H), 7.36 – 7.30 (m, 1H), 7.00 (d, J = 8.7 Hz, 2H), 4.16 (t, J = 5.9 Hz, 2H), 2.79 (dt, J = 8.1, 7.0 Hz, 2H), 2.22 – 2.03 (m, 2H). $^{13}C$ NMR (126 MHz, $CDCl_3$) δ = 158.41, 140.81, 133.93, 128.74, 128.19, 126.75, 126.70, 114.80, 65.72, 33.37, 21.29. MS (ESI) m/z : M+ calcd for 243.0844; found: 243.0861.

**2.3 OPE-SH**

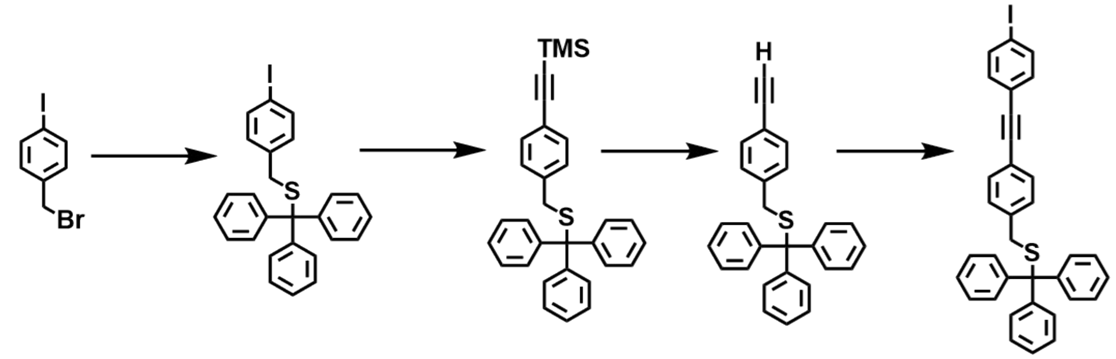


S5 S6 S7 S8

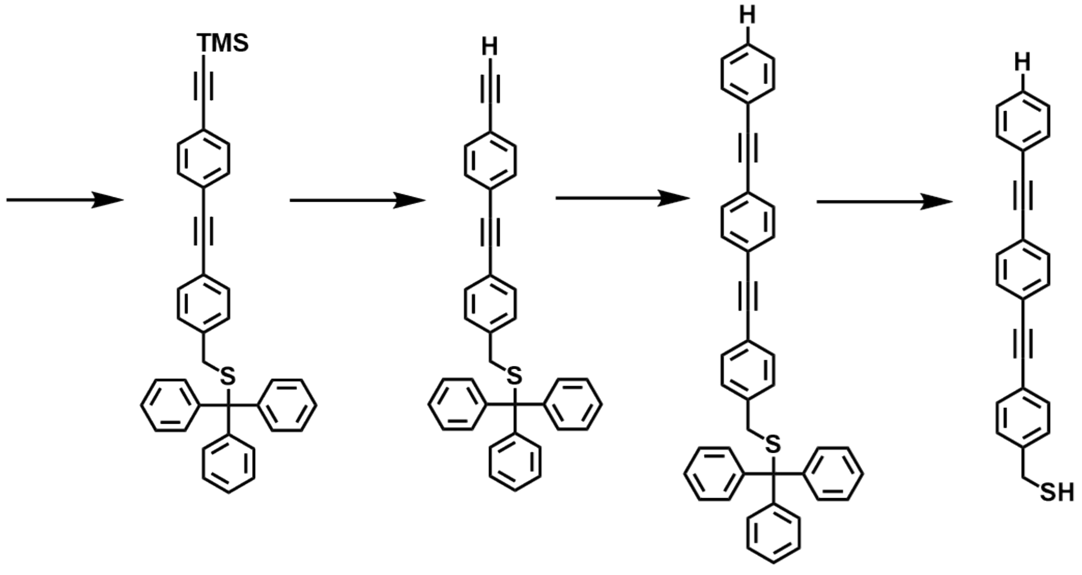


S9 S10 S11 S12

**Synthesis of S5**: 1-(Bromomethyl)-4-iodobenzene (3.0 g, 10 mmol), triphenylmethanethiol (2.76 g, 10 mmol) and potassium carbonate (13.8 g, 100 mmol) were added into acetonitrile (150 mL) in a 250 mL two

necked bottle. After degassing by bubbling with $N_2$ for 15 min, the mixture was then heated at 85 °C for 18 h under $N_2$ atmosphere. The mixture was then cooled down to room temperature, the solid was filtered and then removed solvent by rotary evaporator, the crude product was purified by column chromatography (silica gel, pure petroleum ether) to obtain pure product as a white solid. Yield: 4.92 g, 62%. $^1$H NMR (400 MHz, $CDCl_3$) δ 7.55 (d, $J$ = 8.3 Hz, 2H), 7.47 – 7.42 (m, 6H), 7.30 (dd, $J$ = 10.2, 4.8 Hz, 6H), 7.23 (dd, $J$ = 8.3, 6.2 Hz, 3H), 6.85 (d, $J$ = 8.3 Hz, 2H), 3.25 (s, 2H).

**Synthesis of S6**: The compound **S5** (4.93 g, 10.0 mmol), $Pd(PPh_3)_2Cl_2$ (350 mg, 0.5 mmol), and CuI (190 mg, 1.0 mmol), anhydrous triethylamine (10 mL), anhydrous THF (120 mL) were added into a 250 mL two necked bottle. After degassing by bubbling with $N_2$ for 15 min, the mixture was then heated at 40 °C for 12 h under $N_2$ atmosphere After the mixture was cooled down to room temperature, the solvents were removed in vacuo. The crude product was then purified by column chromatography (silica gel, pure petroleum ether) and obtained pure product as a pale yellow oil. Yield: 4.40 g, 95%.$^1$H NMR (400 MHz, $CDCl_3$) δ 7.44 (d, $J$ = 7.5 Hz, 6H), 7.31 (d, $J$ = 8.1 Hz, 2H), 7.27 (t, $J$ = 7.6 Hz, 6H), 7.19 (t, $J$ = 7.3 Hz, 3H), 7.02 (d, $J$ = 8.1 Hz, 2H), 3.28 (s, 2H), 0.23 (s, 2H).

**Synthesis of S7**: To a solution of **S6** (2.31 g, 5 mmol) in THF (40 mL) and $CH_3OH$ (40 mL), anhydrous $K_2CO_3$ (2.76 g, 20 mmol) was added. Stir the reaction mixture at room temperature for 4 hours. The solid was filtered and then removed solvent by rotary evaporator, the crude product was purified by column chromatography (silica gel, petroleum ether to $CH_2Cl_2$/petroleum ether = 1/9 v/v) to obtain pure product as a white solid. Yield: 1.92 g, 98%. $^1$H NMR (400 MHz, $CDCl_3$) δ 7.50 – 7.45 (m, 6H), 7.37 (d, $J$ = 8.2 Hz, 2H), 7.32 (t, $J$ = 7.5 Hz, 6H), 7.24 (t, $J$ = 7.3 Hz, 3H), 7.08 (d, $J$ = 8.1 Hz, 2H), 3.32 (s, 2H), 3.05 (s, 1H).

**Synthesis of S8**: The compound **S7** (0.62 g, 1.58 mmol), 1,4-Diiodobenzene (2.00 g, 6.32 mmol),, $Pd(PPh_3)_2Cl_2$ (55 mg, 0.079 mmol), and CuI (30 mg, 0.079 mmol), anhydrous triethylamine (15 mL), anhydrous THF (15 mL) were added into a 100 mL Schlenk tube. After degassing by bubbling with $N_2$ for 15 min, the mixture was then heated at 40 °C for 12 h under $N_2$ atmosphere After the mixture was cooled down to room temperature, the solvents were removed in vacuo. The crude product was then purified by column chromatography (silica gel, petroleum ether to $CH_2Cl_2$/petroleum ether = 1/9v/v) and obtained pure product as a white solid. Yield: 4.40 g, 70%. $^1$H NMR (400 MHz, $CDCl_3$) δ 7.70 – 7.64 (m, 2H), 7.49 – 7.43 (m, 6H), 7.38 (d, $J$ = 8.2 Hz, 2H), 7.34 – 7.28 (m, 6H), 7.25 – 7.20 (m, 5H), 7.10 (d, $J$ = 8.2 Hz, 1H), 3.31 (s, 2H).

**Synthesis of S9**: The method was the same as **S6**. The crude product was then purified by column chromatography (silica gel, petroleum ether to $CH_2Cl_2$/petroleum ether = 1/9 v/v) and obtained pure product as viscous transparent liquid. Yield: 2.5g, 65.8%. $^1$H NMR (400 MHz, Chloroform-d) δ 7.49 – 7.45 (m, 6H), 7.43 (s, 4H), 7.38 (d, $J$ = 8.2 Hz, 2H), 7.34 – 7.28 (m, 6H), 7.25 – 7.20 (m, 3H), 7.10 (d, J = 8.1 Hz, 2H), 3.31 (s, 2H).

**Synthesis of S10**: The method was the same as compound **S7**. The crude product was then purified by column chromatography (silica gel, petroleum ether to $CH_2Cl_2$/petroleum ether = 1/4 v/v) and obtained pure product as a white solid. Yield: 1.7g, 78%. $^1$H NMR (400 MHz, Chloroform-d) δ 7.49 – 7.45 (m, 10H), 7.39 (d, $J$ = 8.0 Hz, 2H), 7.34 – 7.27 (m, 6H), 7.25 – 7.20 (m, 3H), 7.10 (d, $J$ = 7.8 Hz, 2H), 3.32 (s, 2H), 3.17 (s, 1H).

**Synthesis of S11**: The method was the same as compound **S8**. The crude product was then purified by column chromatography (silica gel, petroleum ether to $CH_2Cl_2$/petroleum ether = 1/4 v/v) and obtained pure

product as white solid. Yield: 130mg, 37.6%. $^{1}H$ NMR (400 MHz, $CDCl_3$) δ 7.60 – 7.47 (m, 12H), 7.42 (d, $J$ = 8.0 Hz, 2H), 7.40 – 7.23 (m, 12H), 7.13 (d, $J$ = 8.0 Hz, 2H), 3.36 (s, 2H).

**Synthesis of S12**: Precursor compound **S11** (130 mg, 0.23 mmol) was deprotected by dissolution in dry $CH_2Cl_2$ (10 mL), followed by addition of triethylsilane (1 mL), trifluoroacetic acid (0.2 mL) was immediately added in one portion, and the resulting solution swirled for 5 min. The reaction was quenched through addition of 20 mL water, the mixture was extracted three times with $CH_2Cl_2$ (3 × 20 mL), the combined organic phases were then dried over $Na_2SO_4$, filtered and concentrated under reduced pressure. The crude product was then purified by column chromatography (silica gel, $CH_2Cl_2$/petroleum ether = 1/1 v/v) under $N_2$ protect condition and obtained pure product as a white solid. Yield: 70mg, 53.9%. $^{1}H$ NMR (400 MHz, Chloroform-d) δ 7.55 – 7.46 (m, 8H), 7.37 – 7.30 (m, 4H), 3.75 (d, $J$ = 7.6 Hz, 2H), 1.78 (t, $J$ = 7.6 Hz, 1H). $^{13}C$ NMR (101 MHz, $CDCl_3$) δ 147.70, 141.56, 131.93, 131.63, 131.53, 128.47, 128.39, 128.14, 123.12, 123.05, 123.03, 121.77, 91.26, 90.97, 89.26, 89.09, 28.85. TOF-MS m/z calc for $C_{23}H_{16}S$ [M-2H]+ 322.0816, found 322.0846.

**2.4 Fc-$(CH_2)_3$-O-Bp-O-$(CH_2)_3$-SH**

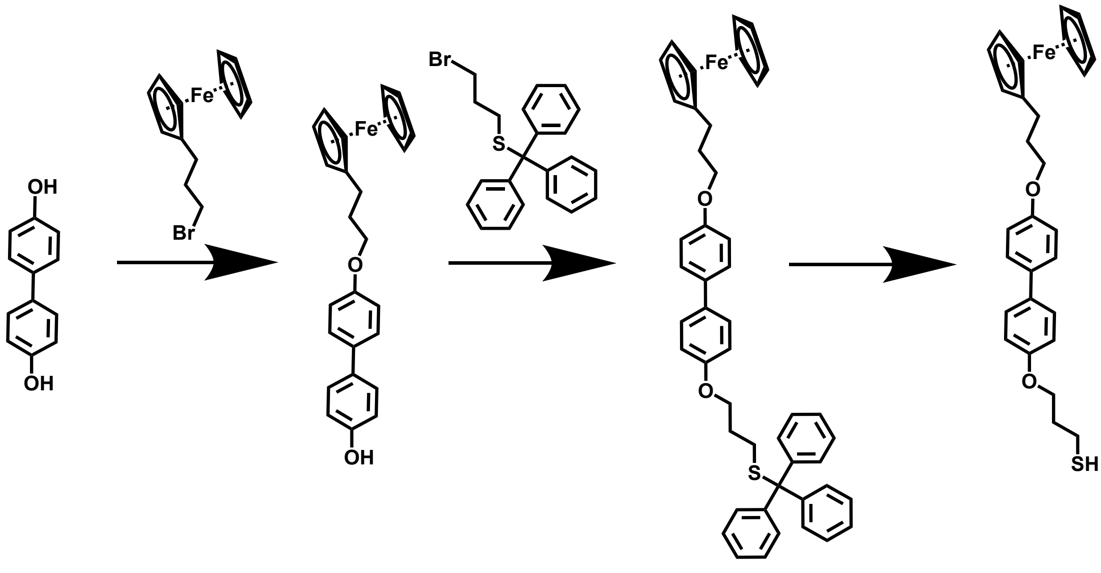

**S13** **S14** **S15**

**Synthesis of S13**: Fc-$(CH_2)_3$-Br (2.0 mmol, 0.6 g) and [1,1'-biphenyl]-4,4'-diol (8.0 mmol, 1.5 g) and $K_2CO_3$ (20.0 mmol, 2.8 g) and NaI (4.0 mmol, 599.6 mg) were mixed in N, N-Dimethylformamide (DMF, 100 ml), the mixture was heated and stirred at 120 °C overnight. Then the mixture cooled down to room temperature, the reaction mixture was poured into a large amount of water and extracted with ethyl acetate. The combined organic layers were washed with water for three times and dried over anhydrous $Na_2SO_4$. After filtration and solvent evaporation, the residue was purified by silica-gel column chromatography using ethyl acetate/petroleum ether as an eluent. A yellow solid was obtained. $^1$H NMR (500 MHz, $CDCl_3$) δ 7.49 – 7.40 (m, 4H), 6.99 – 6.93 (m, 2H), 6.91 – 6.86 (m, 2H), 4.14 – 4.11 (m, 5H), 4.08 (dt, *J* = 15.2, 1.7 Hz, 4H), 4.01 (t, *J* = 6.3 Hz, 2H), 2.58 – 2.50 (m, 2H), 2.02 (tt, *J* = 12.8, 6.3 Hz, 2H). $^{13}$C NMR (101 MHz, $CDCl_3$) δ 158.20, 154.62, 133.75, 133.37, 127.93, 127.70, 115.61, 114.84, 88.32, 68.58, 68.18, 67.47, 67.29, 30.60, 25.98. HRMS (ESI+): m/z calc for $C_{25}H_{24}FeO_2$ [M]+ 412.1120, found 412.1129.

**Synthesis of S14:** Compound **S13** (2.0 mmol, 0.8 g) and Br-$(CH_2)_3$-STr (2.2 mmol, 0.9 g) and $K_2CO_3$ (20.0 mmol, 2.8 g) were mixed in DMF (100 ml), the mixture was heated and stirred at 120 °C overnight. Then

the mixture cooled down to room temperature, the reaction mixture was poured into a large amount of water and extracted with dichloromethane. The combined organic layers were washed with water for three times and dried over anhydrous $Na_2SO_4$. After filtration and solvent evaporation, the residue was purified by silica-gel column chromatography using dichloromethane/petroleum ether as an eluent. $^1H$ NMR (500 MHz, $CDCl_3$) δ 7.47 – 7.40 (m, 10H), 7.37 (m, 6H), 7.30 (m, 3H), 6.94 (dd, *J* = 42.8, 8.7 Hz, 4H), 4.11 – 4.05 (m, 9H), 4.00 (t, *J* = 6.3 Hz, 2H), 3.94 (t, *J* = 6.1 Hz, 2H), 2.54 (t, *J* = 7.7 Hz, 2H), 2.37 (t, *J* = 7.1 Hz, 2H), 2.04 – 1.98 (m, 2H), 1.86 – 1.81 (m, 2H). $^{13}C$ NMR (101 MHz, $CDCl_3$) δ 158.23, 157.95, 144.94, 133.54, 133.46, 129.65, 127.90, 127.72, 127.66, 126.65, 114.86, 88.30, 68.57, 68.19, 67.46, 67.29, 66.69, 66.39, 34.74, 30.66, 28.57, 28.46, 26.03. HRMS (ESI+): m/z calc for $C_{47}H_{44}FeO_2S$ [M]+ 728.2407, found 728.2409.

**Synthesis of S15:** Put **S14** (0.15 mmol, 109.2 mg) into flask under $N_2$ protection, and add 25.0 ml anhydrous dichloromethane to dissolve it. Then add 3 drops of trifluoroacetic acid, the solution turned green could be observed, when mixture color doesn't change anymore, add 0.3 ml $Et_3SiH$ in the mixture. Keep reacting for 30 minutes, 10.0 ml deionized water was added to the mixture for quenching. The reaction system returned to yellow after quenching. The mixture then extracted with dichloromethane. The combined organic layers were washed with water for three times and dried over anhydrous $Na_2SO_4$. After filtration and solvent evaporation, the residue was purified by silica-gel column chromatography with $N_2$ protection and using dichloromethane/hexane as an eluent. $^1H$ NMR (500 MHz, $CDCl_3$) δ 7.51 – 7.44 (m, 4H), 6.99 – 6.93 (m, 4H), 4.15 – 4.05 (m, 11H), 4.01 (t, *J* = 6.3 Hz, 2H), 2.76 (dd, *J* = 15.0, 7.0 Hz, 2H), 2.60 – 2.50 (m, 2H), 2.15 – 2.07 (m, 2H), 2.06 – 1.98 (m, 2H), 1.42 (t, *J* = 8.1 Hz, 1H). $^{13}C$ NMR (126 MHz, $CDCl_3$) δ 158.18, 157.90, 127.74, 114.76, 114.73, 88.24, 68.53, 68.15, 67.37, 67.25, 33.38, 30.60, 25.97, 21.31. MS (ESI) m/z : M+ calcd for 486.1310; found: 486.1310.MS (ESI) m/z : M+ calcd for 486.1310; found: 486.1307. CHN analysis: C 68.64%, H 6.22%.

**2.5 Fc-$(CH_2)_3$-NDI-$(CH_2)_3$-SH**

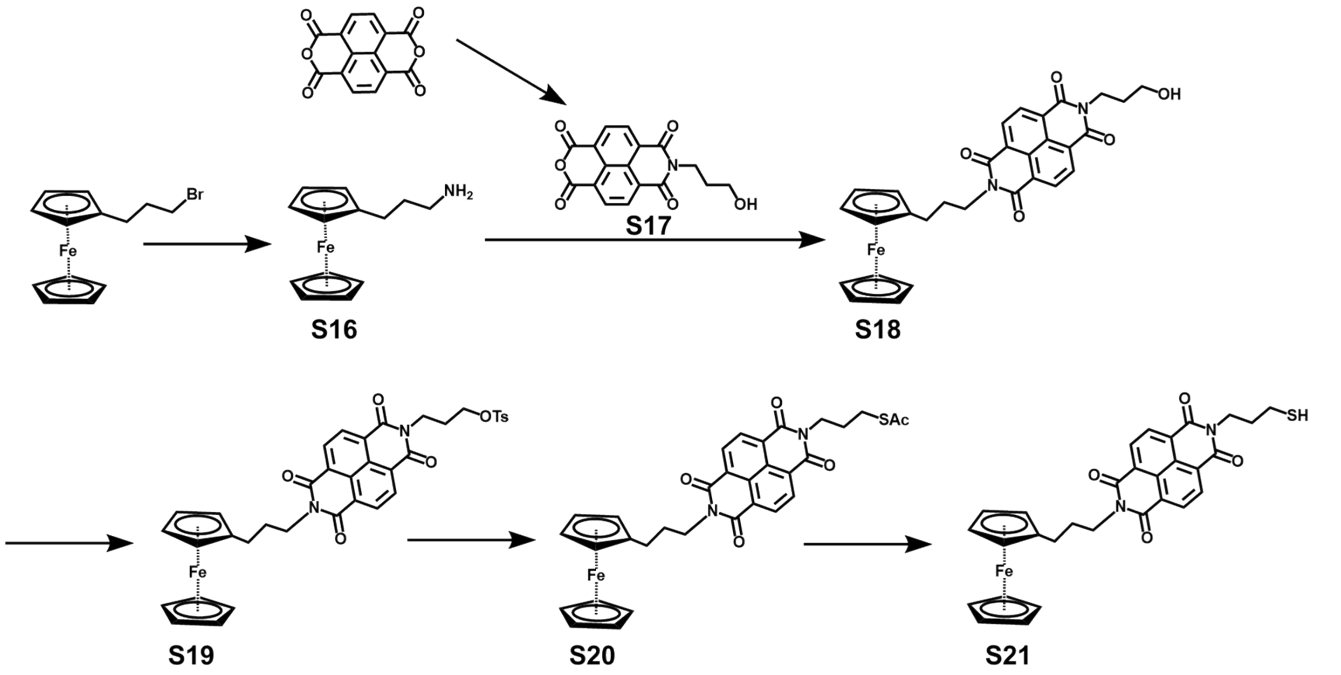


**Synthesis of S16:** Put Fc-$(CH_2)_3$-Br (12.0 mmol, 3.7 g) and phthalimide potassium (18.0 mmol, 5.5 g) into flask under $N_2$ protection, and add anhydrous N, N-dimethylformamide to dissolve it. Then the solution was heated to 70 °C and stirred for 5 h. The mixture was cooled to room temperature and removed the solvent. The residue was purified by silica-gel column chromatography using dichloromethane/ ethyl acetate/ methanol as eluent. Put Fc-$(CH_2)_3$-phthalimide (8 mmol, 3.0 g) into flask under $N_2$ protection and add 200 ml ethanol. Hydrazine hydrate (28 mmol, 1.4 g) was added and the mixture was refluxed for 4 h. After the mixture was cooled to room temperature, 1.0 mol/L hydrous potassium hydroxide (120 ml) was added. The crude product was extracted with dichloromethane and purified by silica-gel column chromatography using chloroform as an eluent. $^1$H NMR (500 MHz, $CDCl_3$) δ 4.09 – 4.03 (m, 9H), 2.72 (t, $J$ = 7.1 Hz, 2H), 2.36 (t, $J$ = 7.8 Hz, 2H), 1.65 (m, 2H), 1.38 (s, 2H).

**Synthesis of S17:** Put 1,4,5,8-Naphthalenetetracarboxylic dianhydride (18.7 mmol, 5.0 g) and potassium hydroxide (75 mmol, 4.2 g) into flask under $N_2$ protection, and add 125 ml $H_2O$ to make clear solution. Then the solution was acidified to pH = 6.4 using 1.0 mol/L phosphoric acid. $NH_2$-$(CH_2)_3$-OH (18.7 mmol, 1.85

ml) was added and the solution was again acidified to pH = 6.4 using 1 M phosphoric acid. After that, the mixture was heated to 110 °C and reacted for 6 h. The mixture was cooled to room temperature and filtered. Add 1 M hydrochloric acid into the filtrate to gain pale solid and washed with water. After dried over vacuum, the product can be used without further purification.

**Synthesis of S18:** Put compound **S16** (5.0 mmol, 1.2 g) and **S17** (5.0 mmol, 1.6 g) into flask under $N_2$ protection, and add anhydrous N, N-dimethylformamide to dissolve it. The mixture was heated to 110 °C and reacted for 24h. After that, the mixture was extracted with dichloromethane and washed with brine 3 times. After dried over $Na_2SO_4$ and solvent evaporation, the residue was purified by silica-gel column chromatography with $N_2$ protection and using chloroform as an eluent. $^1H$ NMR (500 MHz, $CDCl_3$) δ 8.77 (s, 4H), 4.37 (t, $J$ = 6.3 Hz, 2H), 4.27 – 4.23 (m, 2H), 4.12 (s, 2H), 4.10 (s, 5H), 4.02 (s, 2H), 3.64 (t, $J$ = 5.7 Hz, 2H), 2.48 (t, $J$ = 7.8 Hz, 2H), 2.10 – 1.93 (m, 4H). $^{13}C$ NMR (126 MHz, $CDCl_3$) δ 163.42, 162.70, 131.24, 130.96, 126.83, 126.68, 126.66, 126.21, 68.59, 67.96, 67.25, 59.14, 40.94, 37.50, 30.90, 28.93, 26.99. HRMS (ESI+): m/z calc for $C_{30}H_{26}FeN_2O_5$ [M]+ 550.1186, found 550.1188.

**Synthesis of S19:** Put compound **S18** (1.0 mmol, 550.0 mg) into flask under $N_2$ protection, and add 50.0 ml anhydrous dichloromethane. The suspension was cooled in ice bath. Then 4-dimethylaminopyridine (50.0 mg) and 2.8 ml triethylamine was added into flask. The Tosyl chloride (3.5 mmol, 665.0 mg) was dissolved in 20.0 ml anhydrous dichloromethane and added dropwise into suspension. The mixture was warmed to room temperature and stirred for 24 h. After that, the mixture was washed with brine 3 times and extracted with dichloromethane. After dried over $Na_2SO_4$ and solvent evaporation, the residue was purified by silica-gel column chromatography with $N_2$ protection and using dichloromethane/ethyl acetate as an eluent. $^1H$ NMR (500 MHz, $CDCl_3$) δ 8.73 (q, $J$ = 7.6 Hz, 4H), 7.73 (d, $J$ = 8.3 Hz, 2H), 7.29 (d, $J$ = 8.0 Hz, 2H), 4.28

– 4.24 (m, 4H), 4.18 (t, *J* = 6.1 Hz, 2H), 4.11 (s, 2H), 4.09 (s, 5H), 4.01 (s, 2H), 2.48 (m, 2H), 2.42 (s, 3H), 2.14 (m, 2H), 1.99 (m, 2H). $^{13}$C NMR (126 MHz, $CDCl_3$) δ 162.75, 162.72, 144.76, 132.75, 131.03, 130.90, 129.76, 127.85, 126.69, 126.62, 126.28, 68.55, 68.26, 67.93, 67.22, 40.91, 37.65, 28.90, 27.47, 26.96, 21.62. HRMS (ESI+): m/z calc for $C_{37}H_{32}FeN_2O_7S$ [M]+ 704.1275, found 704.1276.

**Synthesis of S20:** Put **S19** (1.0 mmol, 704.6 mg) into flask under $N_2$ protection, and add 50.0 ml anhydrous N, N-dimethylformamide to dissolve it. Potassium thioacetate (1.3 mmol, 148.5 mg) was added into flask. The mixture was stirred at room temperature overnight. After that, the mixture was washed with brine 3 times and extracted with dichloromethane. After dried over $Na_2SO_4$ and solvent evaporation, the residue was purified by silica-gel column chromatography with $N_2$ protection and using dichloromethane/ ethyl acetate as an eluent. $^1$H NMR (500 MHz, $CDCl_3$) δ 8.75 (s, 4H), 4.26 (m, 4H), 4.10 (s, 2H), 4.08 (s, 5H), 4.00 (s, 2H), 2.98 (t, *J* = 7.1 Hz, 2H), 2.49 (t, *J* = 7.8 Hz, 2H), 2.33 (s, 3H), 2.09 – 1.97 (m, 4H). $^{13}$C NMR (126 MHz, $CDCl_3$) δ 195.48, 162.84, 162.76, 131.04, 130.94, 126.72, 126.70, 126.44, 88.09, 68.51, 67.90, 67.18, 40.93, 39.77, 30.59, 28.95, 28.18, 27.00, 26.60. HRMS (ESI+): m/z calc for $C_{32}H_{28}FeN_2O_5S$ [M]+ 608.1063, found 608.1061.

**Synthesis of S21:** Put **S20** (0.1 mmol, 60.8 mg) into flask under $N_2$ protection, and add 30.0 ml anhydrous tetrahydrofuran to dissolve it. Sodium borohydride (1.00 mmol, 37.8 mg) was dissolved in anhydrous tetrahydrofuran and was added dropwise into flask in ice bath. The mixture was warmed to room temperature and stirred overnight. The mixture then extracted with dichloromethane. After solvent evaporation, the residue was purified by silica-gel column chromatography with $N_2$ protection and using dichloromethane/ ethyl acetate as an eluent. $^1$H NMR (500 MHz, CDC13) δ 8.76 (s, 4H), 4.33 (t, *J* = 7.1 Hz, 2H), 4.25 (t, *J* = 7.0 Hz, 2H), 4.10 (t, *J* = 1.8 Hz, 2H), 4.08 (s, 5H), 4.00 (t, *J* = 1.9 Hz, 2H), 2.64 (q, *J* = 7.4

Hz, 2H), 2.49 (m, 2H), 2.12 – 1.99 (m, 4H). $^{13}C$ NMR (126 MHz, CDCl3) δ 162.91, 162.85, 162.76, 131.06, 130.96, 126.75, 126.71, 126.44, 88.07, 68.50, 67.90, 67.18, 40.95, 39.55, 32.31, 30.60, 29.68, 28.96, 28.18, 27.01, 26.61, 22.23. MS (ESI) m/z : M+ calcd for 566.0963; found: 566.0957. CHN analysis: C 63.39%, H 4.65%, N 4.64%.

**2.6 Fc-OPE-SH**

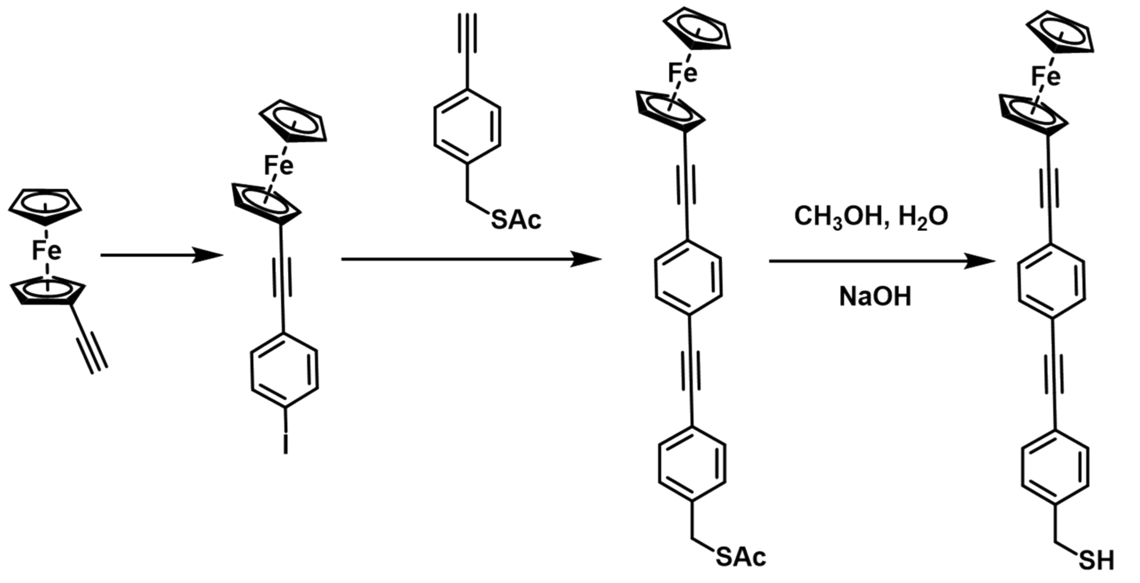


S22 S23 S24

**Synthesis of S22:** The ethynylferrocene (0.52 g, 2.500 mmol), 1,4-diiodobenzene (3.29 g, 10.00 mmol), $Pd(PPh_3)_2Cl_2$ (87.5 mg, 0.125 mmol), and CuI (47.5 mg, 0.25 mmol) were dissolved in anhydrous THF (20 ml) in a Schlenk tube, then 5 mL N,N-diisopropylethylamine was added. The mixture was deoxygenated using the freeze-pump-thaw method (three cycles). The mixture was then stirred at 50 °C for 18 h. When the reaction was complete, the solvents were removed in vacuo and the desired product was isolated by column chromatography (DCM/hexane 1:9) to provide product (721 mg) in 70% yield as an orange solid. $^1$H NMR (400 MHz, $CDCl_3$) δ 7.65 (d, $J$ = 8.5 Hz, 2H), 7.21 (d, $J$ = 8.5 Hz, 2H), 4.50 (t, $J$ = 1.8 Hz, 2H), 4.32 – 4.19 (m, 7H). $^{13}$C NMR (101 MHz, $CDCl_3$) δ 137.42, 132.89, 123.52, 93.17, 90.06, 84.85, 71.44, 69.99, 68.98, 64.81. $^{13}$C NMR (101 MHz, $CDCl_3$) δ 137.42, 132.89, 123.52, 93.17, 90.06, 84.85, 71.44, 69.99, 68.98, 64.81. HRMS (ESI) m/z : $C_{18}H_{13}FeI$ M+ calcd for 411.9406; found: 411.9402.

**Synthesis of S23:** The **S22** (412 mg, 1.00 mmol), S-(4-ethynylbenzyl) ethanethioate (228 mg, 1.20 mmol), $Pd(PPh_3)_2Cl_2$ (35 mg, 0.05 mmol), and CuI (19 mg, 0.10 mmol) were dissolved in anhydrous THF 15 mL in a Schlenk tube, then 5 mL N,N-diisopropylethylamine was added. The reaction mixture was deoxygenated

using the freeze-pump-thaw method (three cycles). The mixture was then stirred at 50 °C for 18 h. When the reaction was complete, the solvents were removed in vacuo and the desired product was isolated by column chromatography (DCM/hexane 1:1) to provide product (308 mg) in 65% yield as an orange solid. $^1$H NMR (400 MHz, $CDCl_3$) δ 7.52 – 7.41 (m, 6H), 7.29 (br, 2H), 4.52 – 4.50 (m, 2H), 4.28 – 4.24 (m, 7H), 4.12 (s, 2H), 2.36 (s, 3H). $^{13}$C NMR (101 MHz, $CDCl_3$) δ 194.87, 138.06, 131.81, 131.47, 131.27, 128.89, 123.88, 122.31, 122.11, 90.67, 90.57, 89.54, 85.54, 71.47, 70.01, 68.98, 64.95, 33.29, 30.33. MS (ESI) m/z : $C_{29}H_{22}FeOS$ M+ calcd for 474.0736; found: 474.0739.

**Synthesis of S24:** Sodium hydroxide (200 mg, 5.00 mmol) was dissolved in 8 mL of deionized water and transferred to a 25 mL round bottom flask. Next, S23 (308 mg, 0.65 mmol) was dissolved in 2 mL of MeOH and added dropwise to the NaOH solution, and the reaction was stirred at room temperate overnight. The reaction was extracted with DCM (3 x 15 mL) and the combined organic extracts were dried over anhydrous $Na_2SO_4$ and concentrated under reduced pressure. Compound 3 was isolated as a orange solid by column chromatography (56 mg, 20% yield) using DCM/hexanes (1/3-1/1, v/v). $^1$H NMR (400 MHz, $CDCl_3$) δ 7.55 – 7.42 (m, 6H), 7.32 (d, $J$ = 8.2 Hz, 2H), 4.51 (t, $J$ = 1.8 Hz, 2H), 4.30 – 4.21 (m, 7H), 3.75 (d, $J$ = 7.6 Hz, 2H), 1.78 (t, $J$ = 7.6 Hz, 1H). $^{13}$C NMR (101 MHz, CDCl3) δ 141.61, 132.04, 131.62, 131.43, 128.27, 124.03, 122.48, 122.03, 90.87, 90.73, 89.60, 85.70, 71.62, 70.15, 69.13, 65.10, 28.99. MS (MALDI-TOF) m/z : M+ calcd for 432.0635; found: 432.0639. CHN analysis: C 72.26%, H 4.89%

**2.7 NMR and MS spectra of novel compounds**

We have synthesized some compounds that have not been reported before. Here we show the NMR and MS spectra of novel compounds.

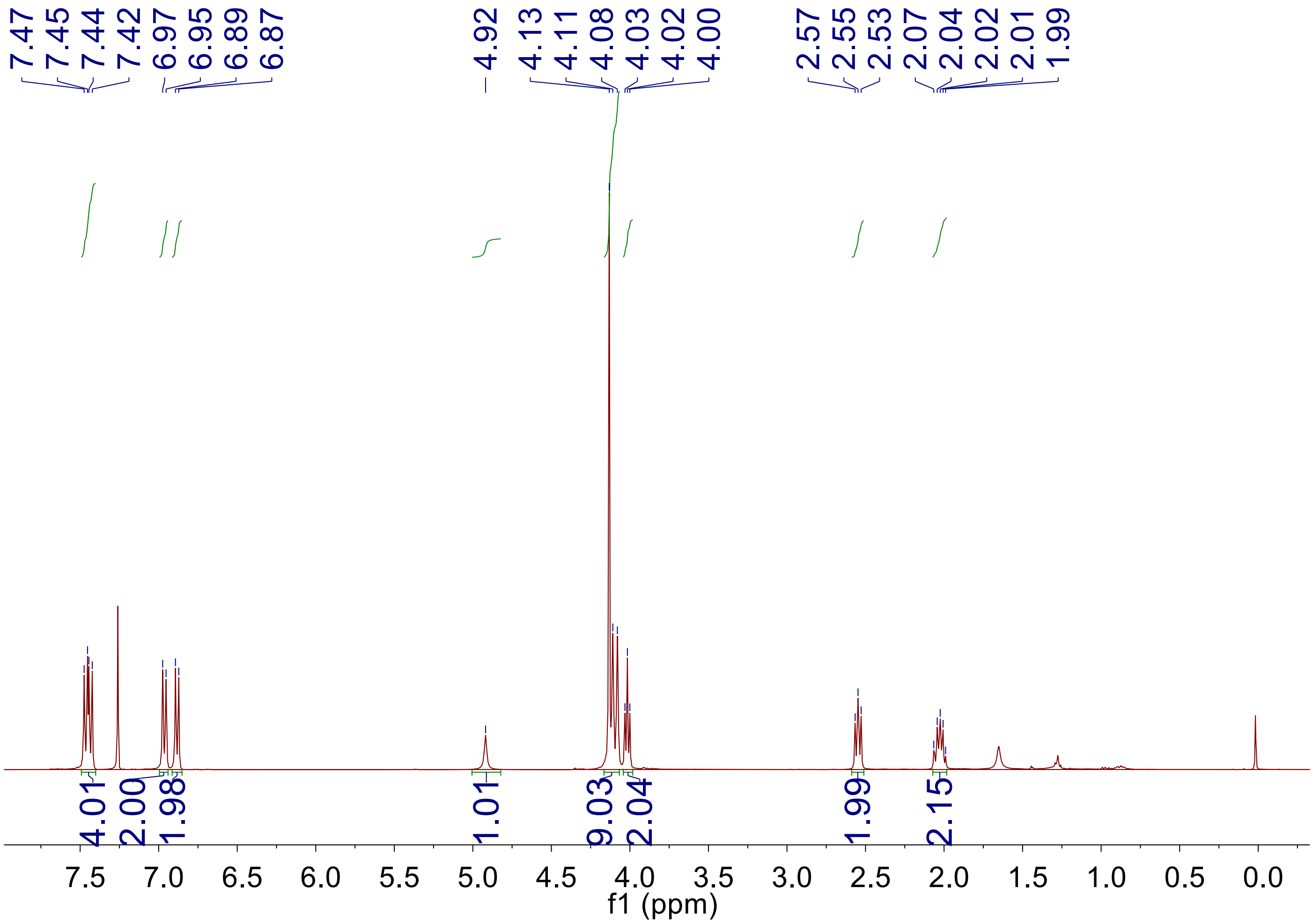


$^1$H NMR spectrum of compound **S13** in CDCl3.

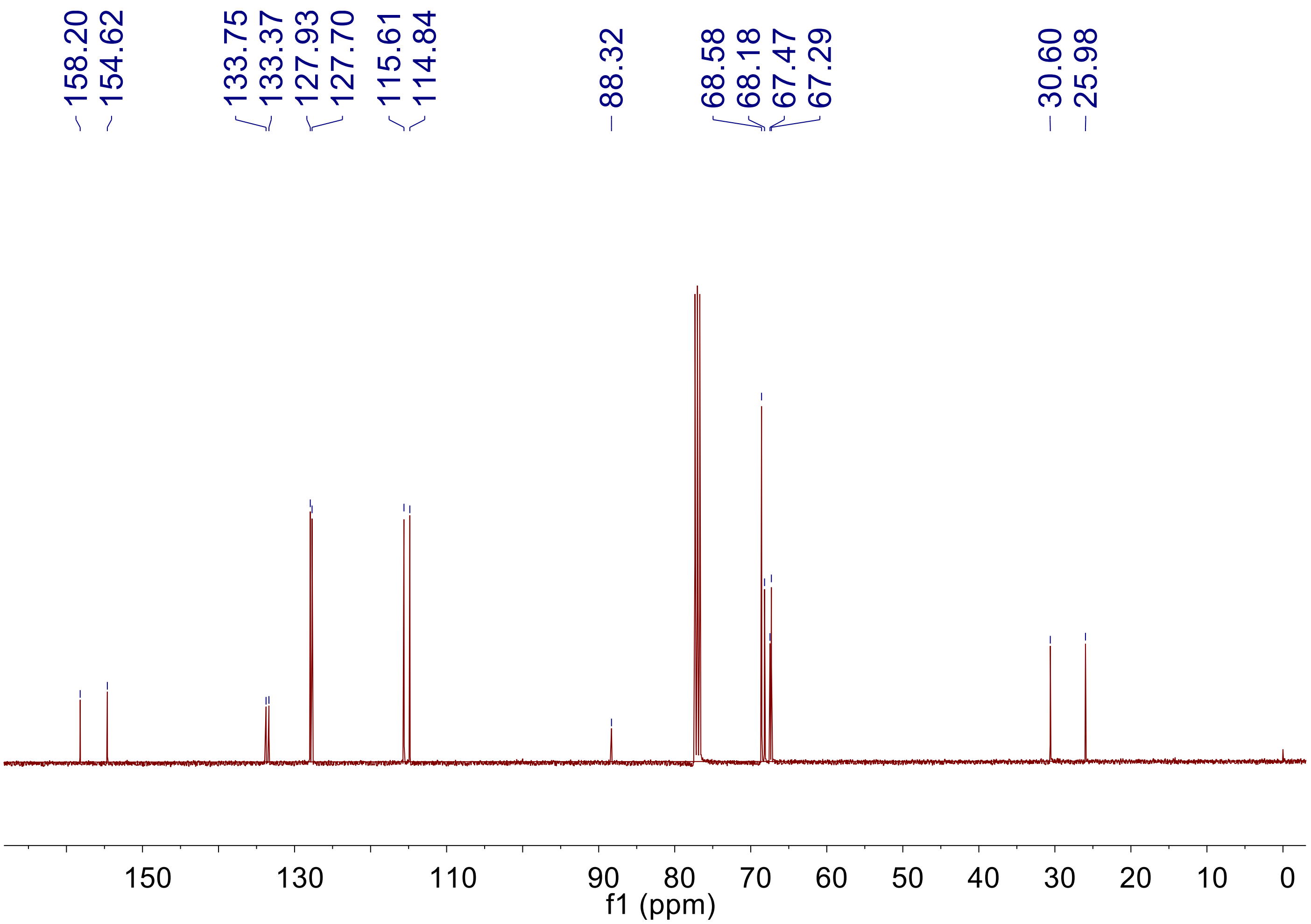


$^{13}$C NMR spectrum of compound **S13** in CDCl3.

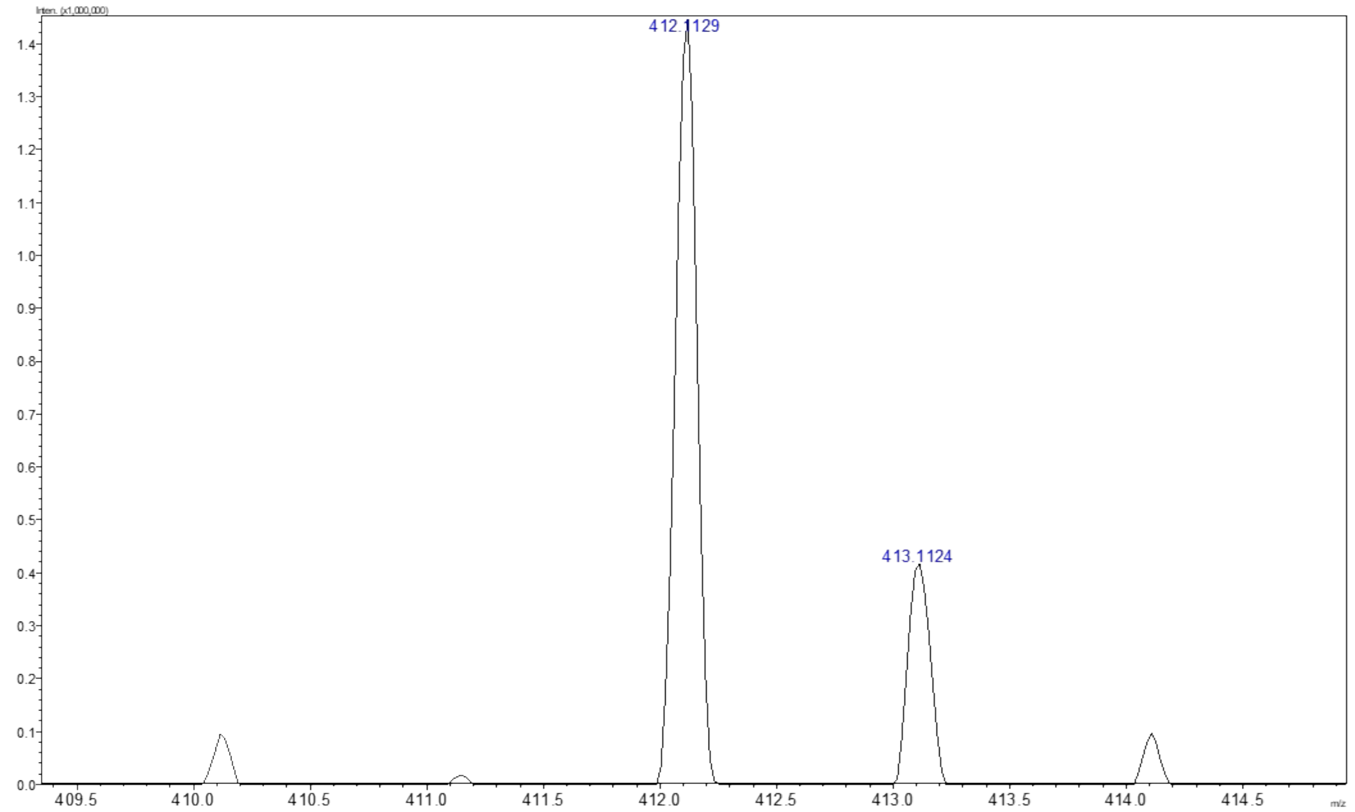


ESI-MS spectrum of compound **S13**.

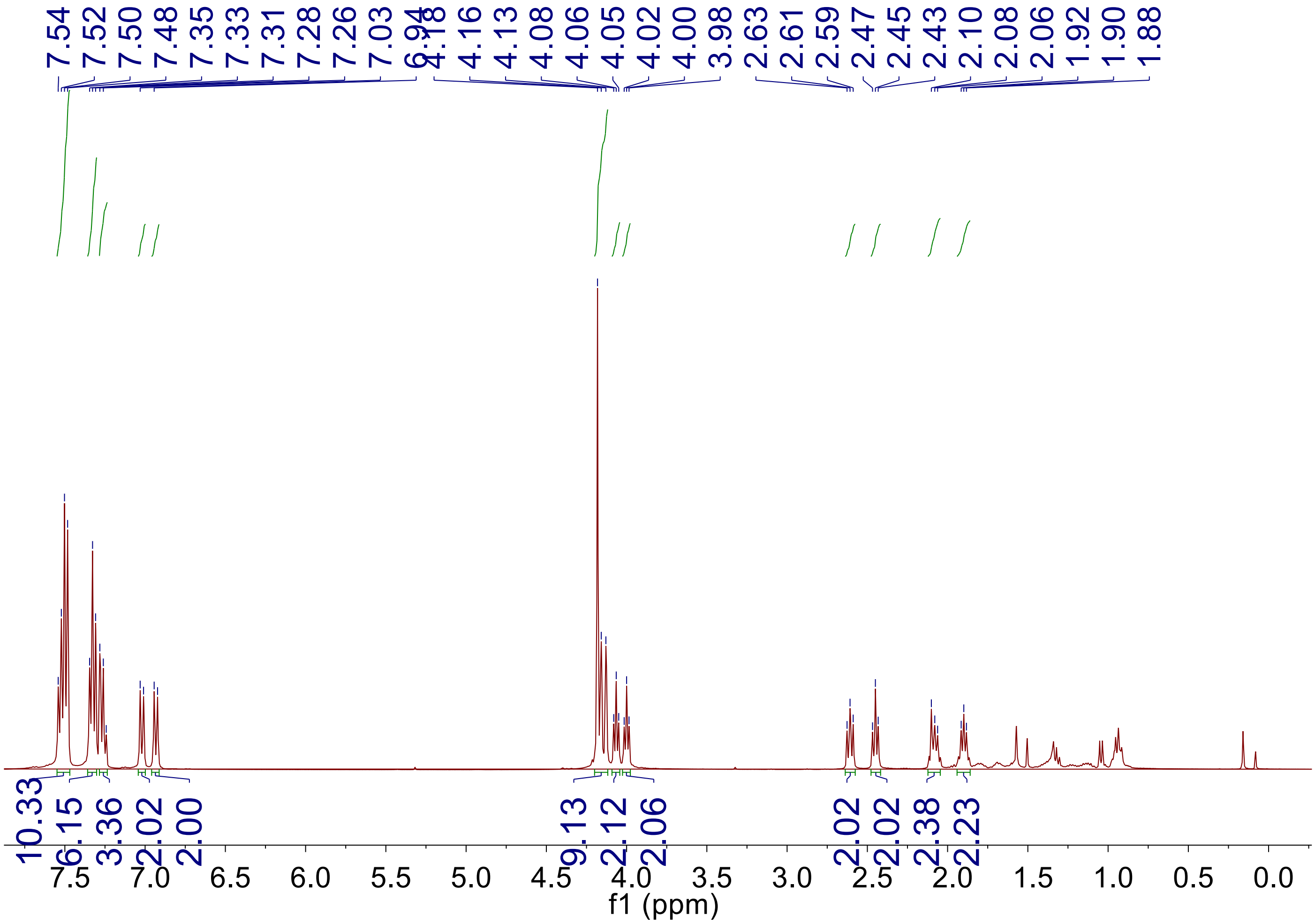


$^{1}$H NMR spectrum of compound **S14** in CDCl3.

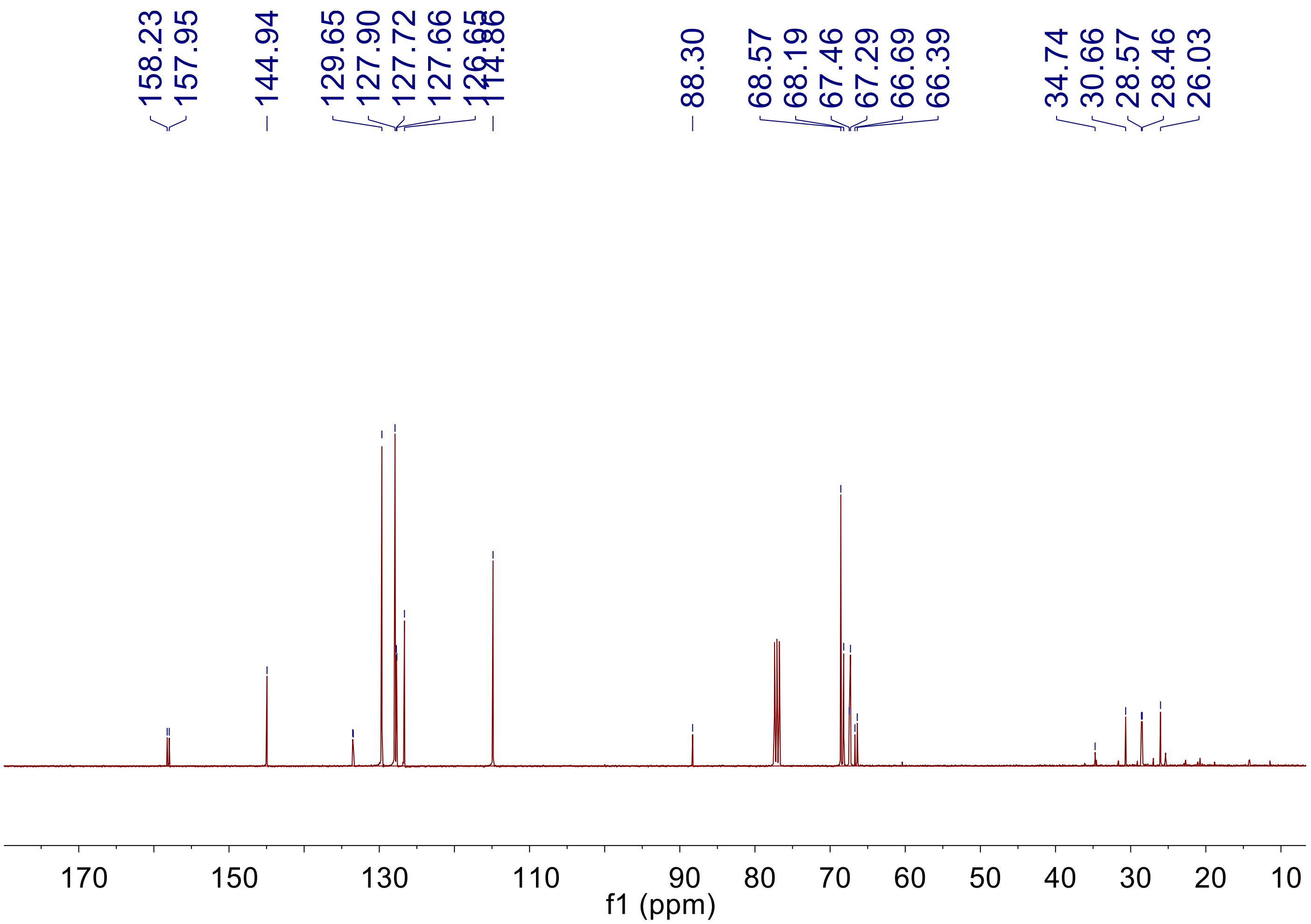


$^{13}$C NMR spectrum of compound **S14** in CDCl3.

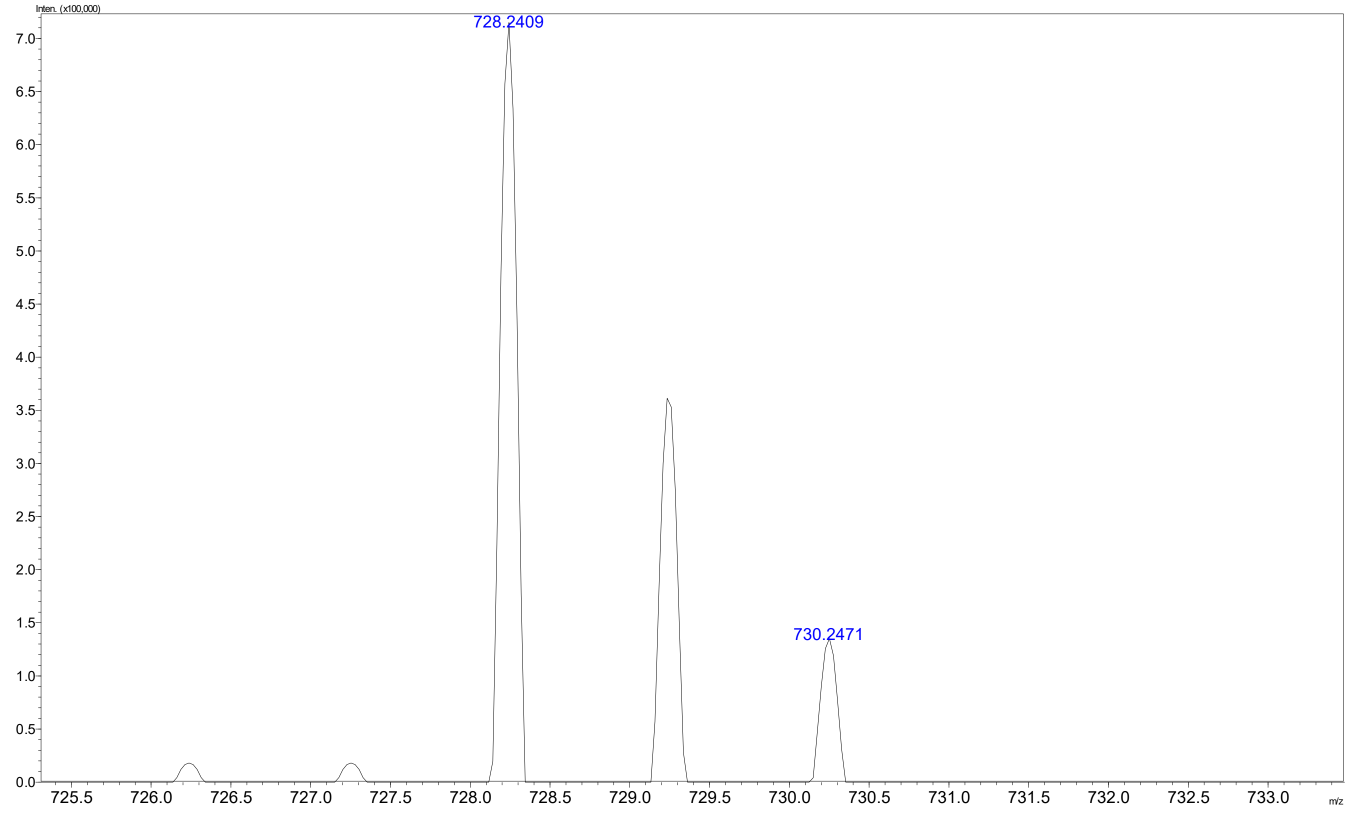


ESI-MS spectrum of compound **S14**.

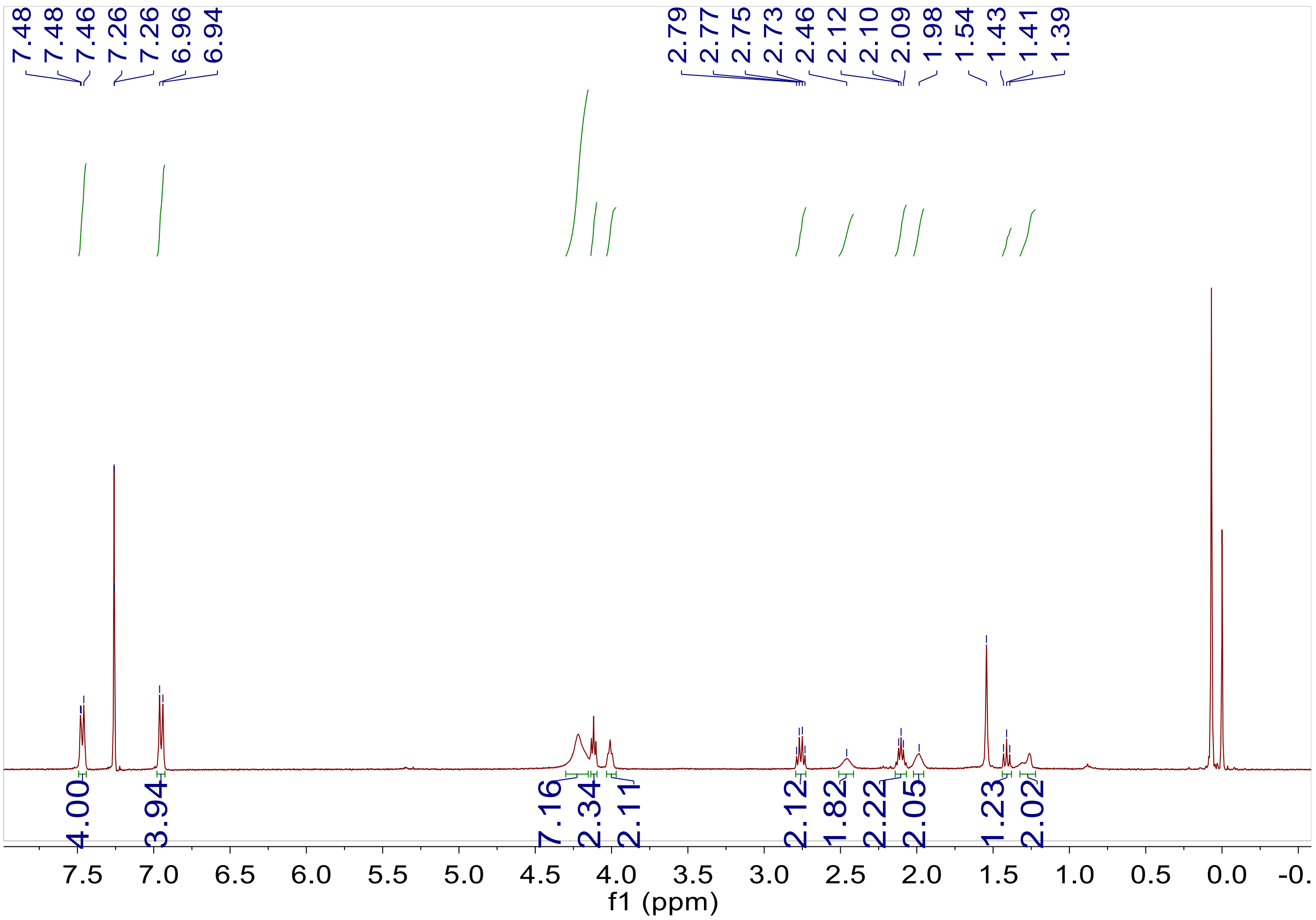


$^1$H NMR spectrum of compound **S15** in CDCl3.

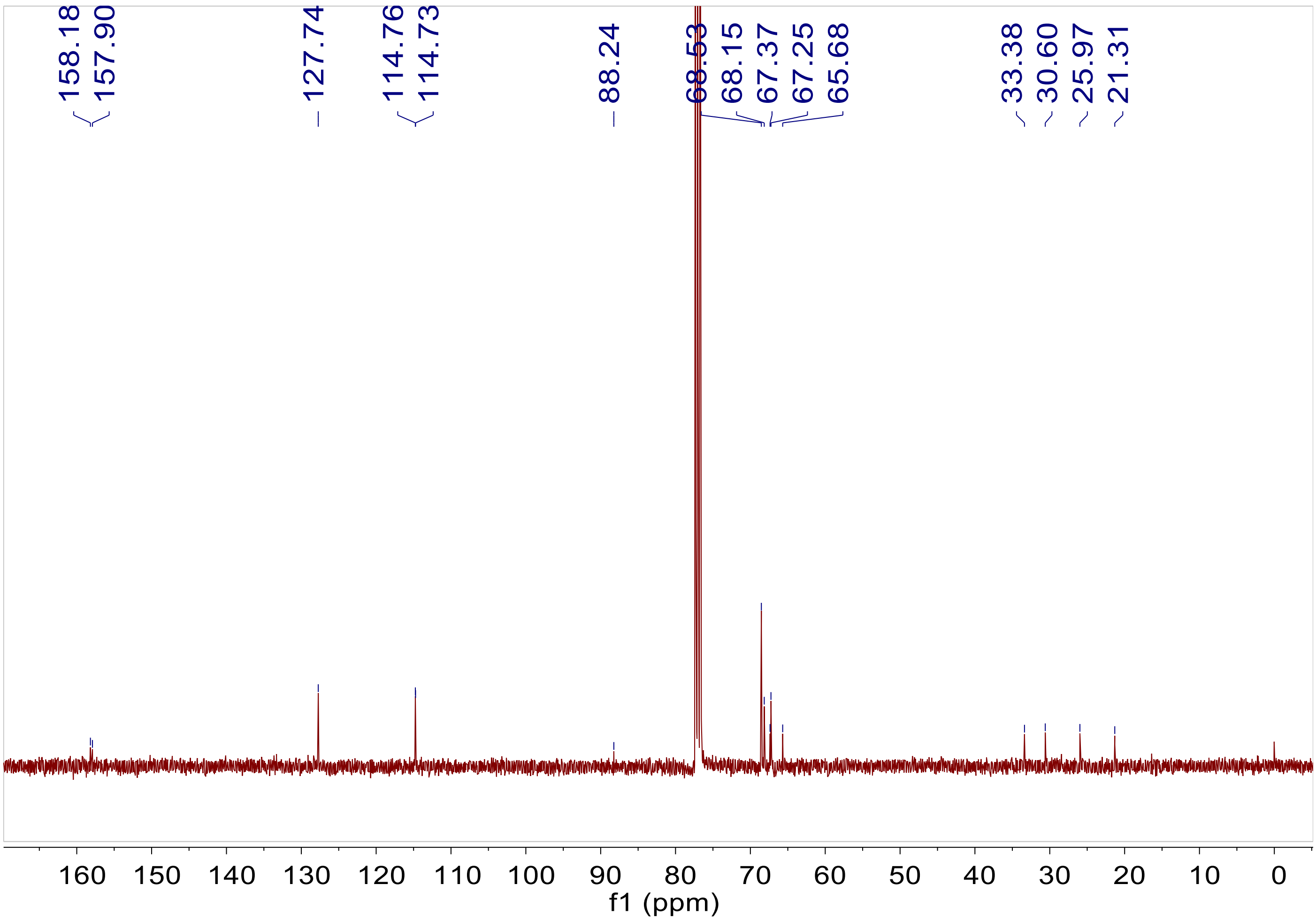


$^{13}$C NMR spectrum of compound **S15** in CDCl3.

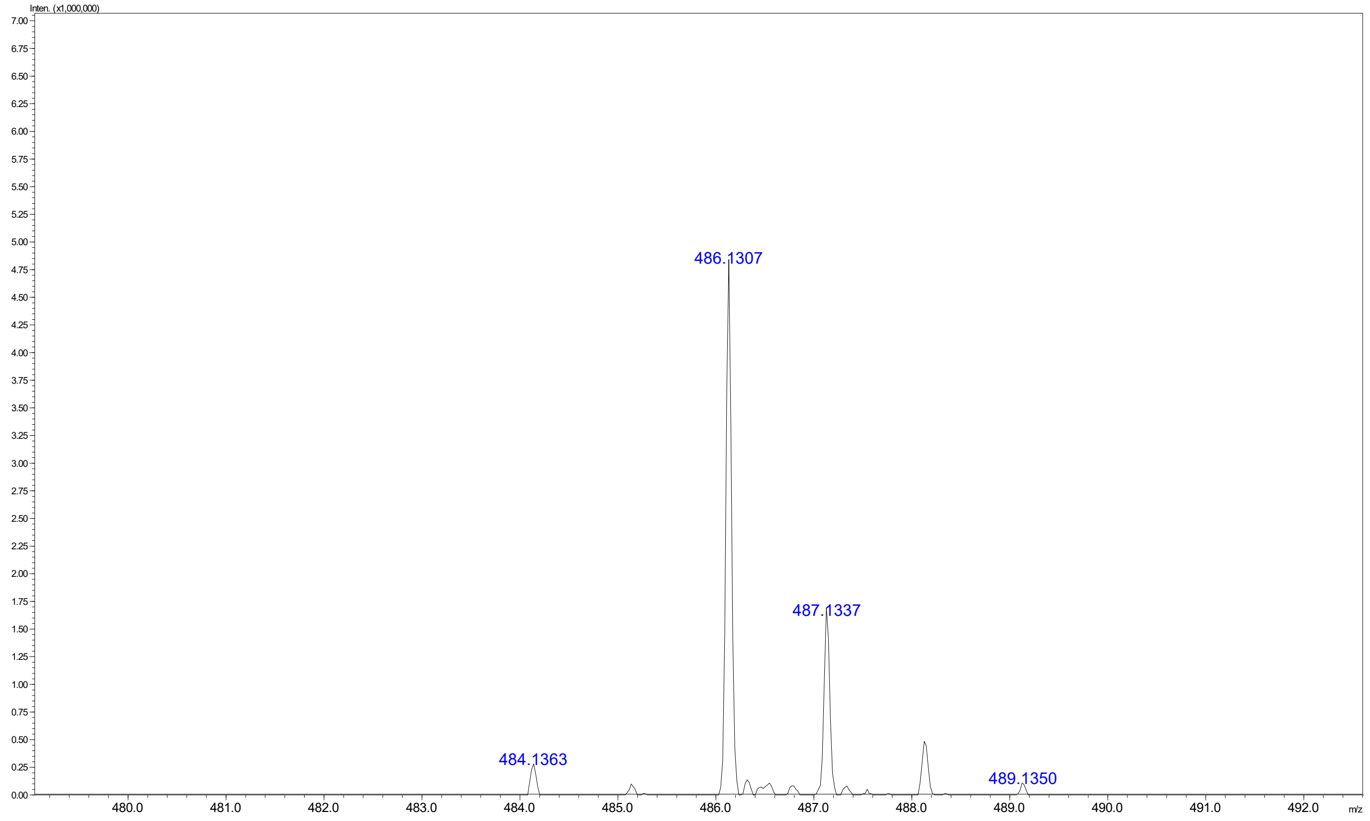


ESI-MS spectrum of compound **S15**.

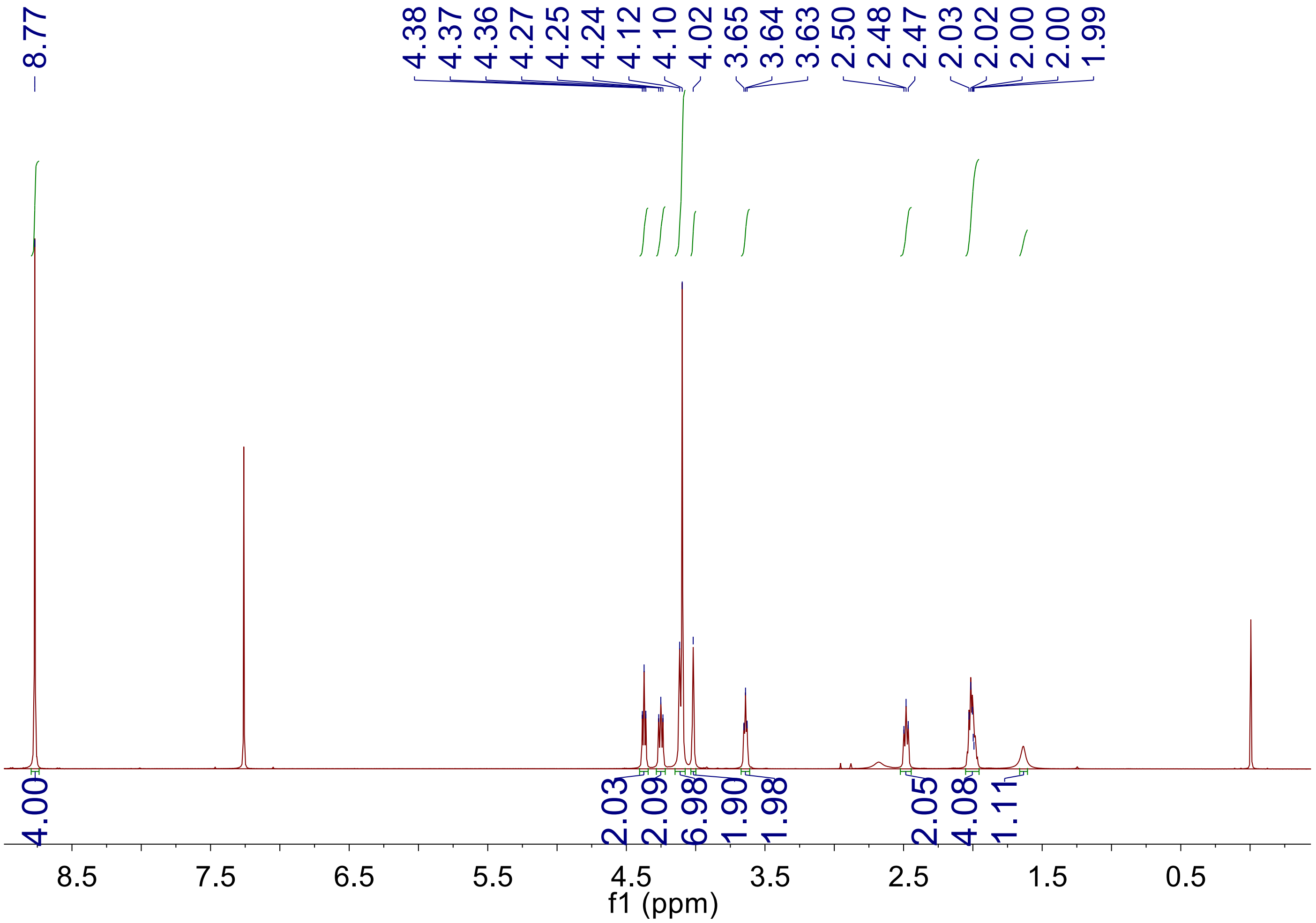


$^{1}$H NMR spectrum of compound **S18** in CDCl3.

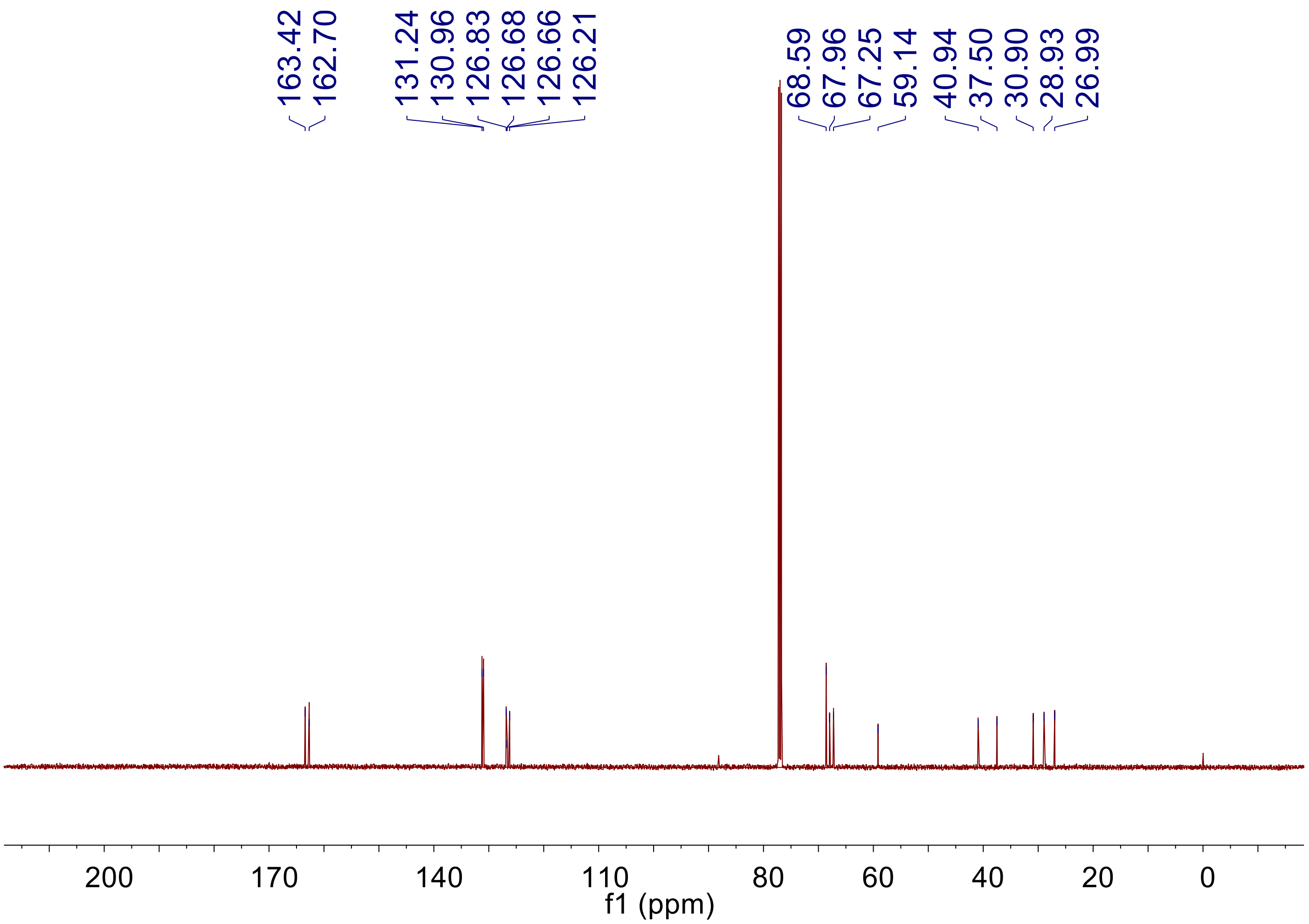


$^{13}$C NMR spectrum of compound **S18** in CDCl3.

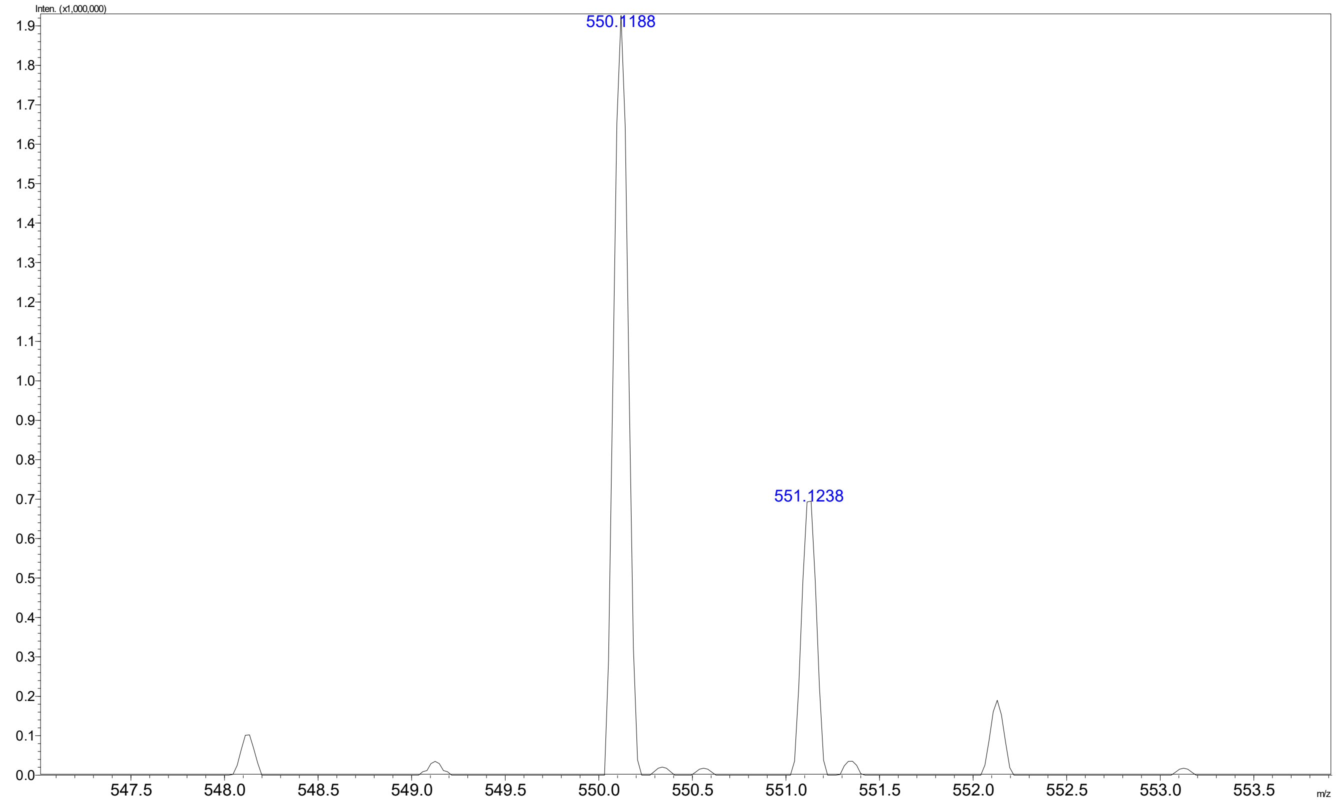


ESI-MS spectrum of compound **S18**.

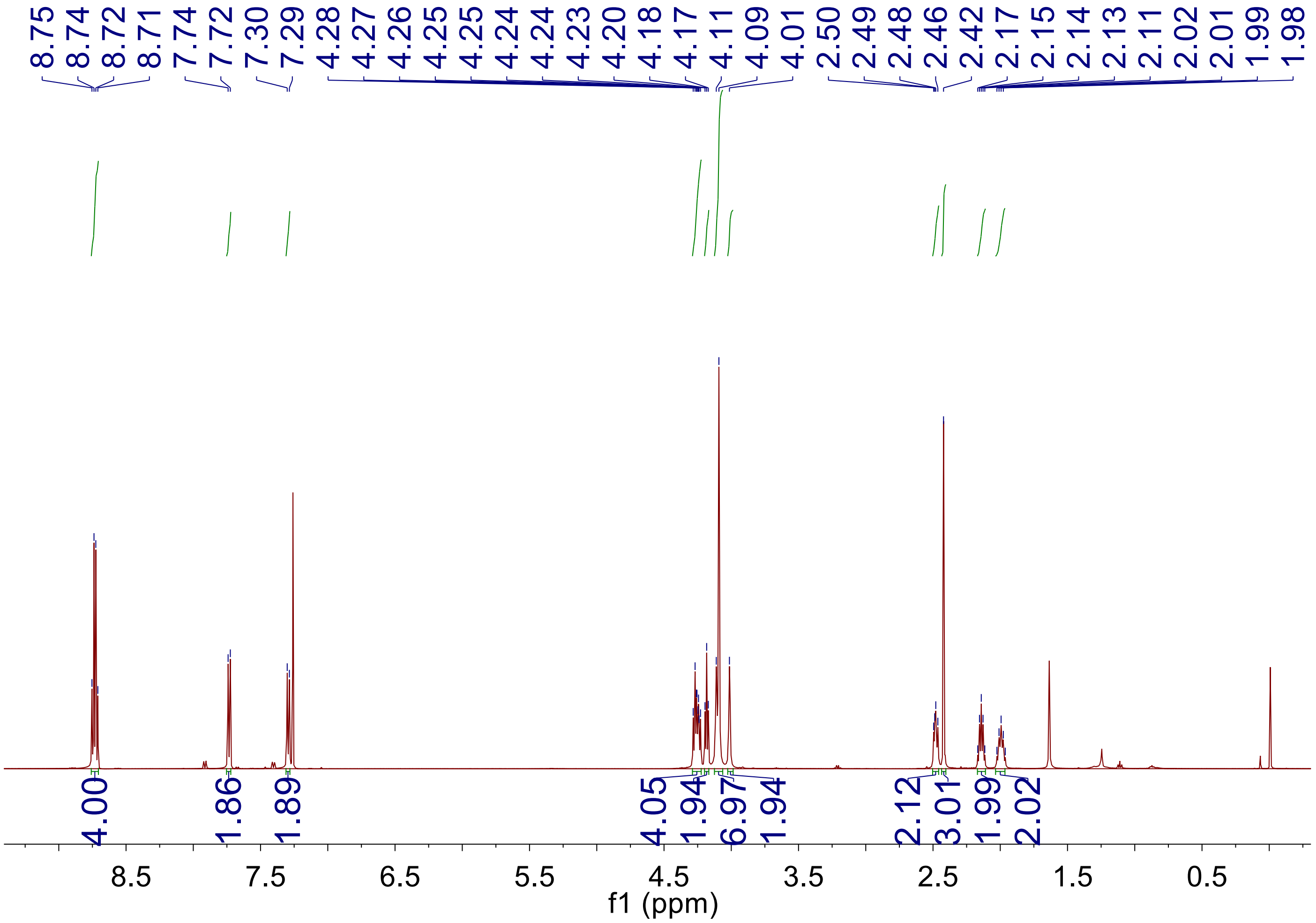


$^{1}$H NMR spectrum of compound **S19** in CDCl3.

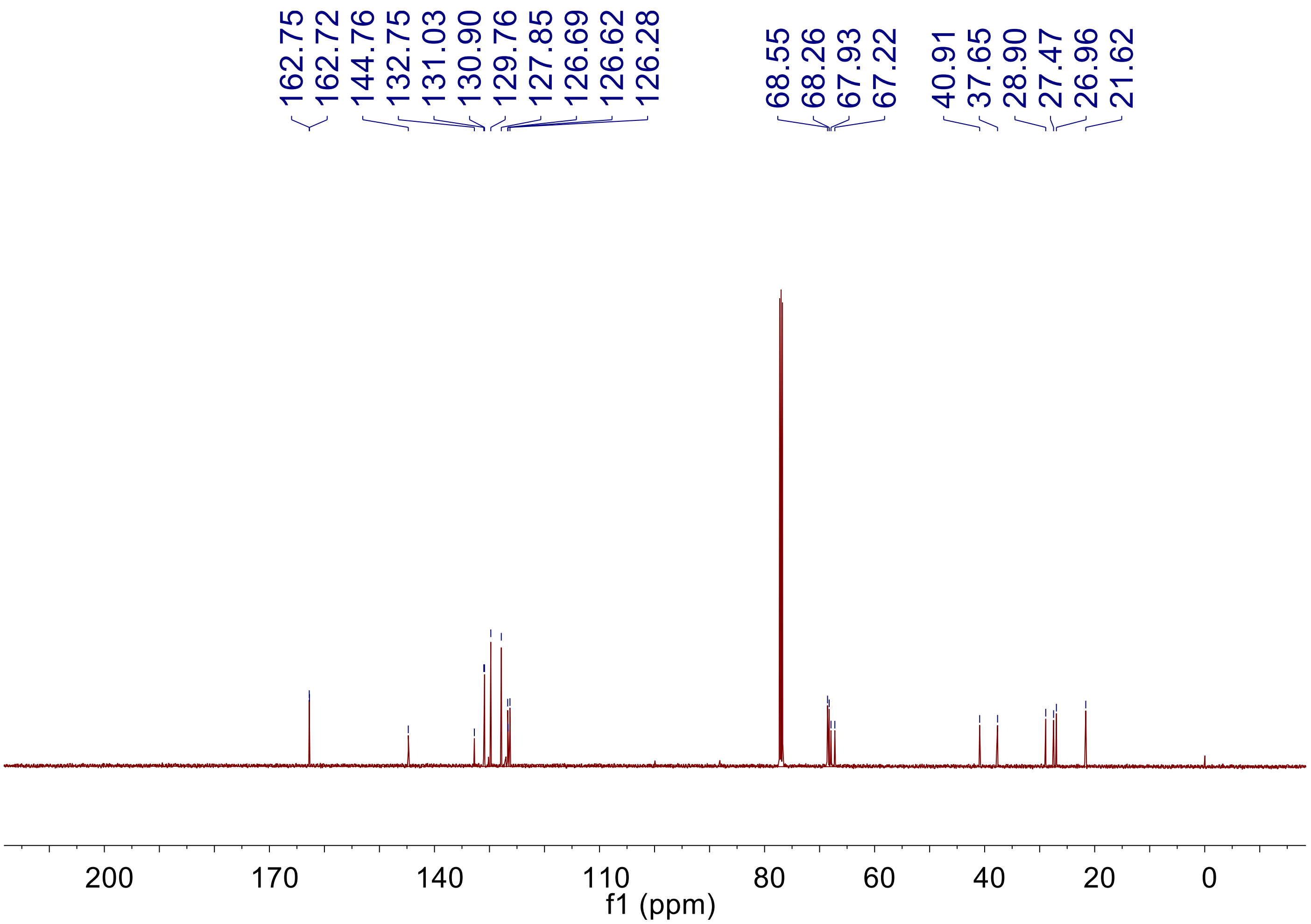


$^{13}$C NMR spectrum of compound **S19** in CDCl3.

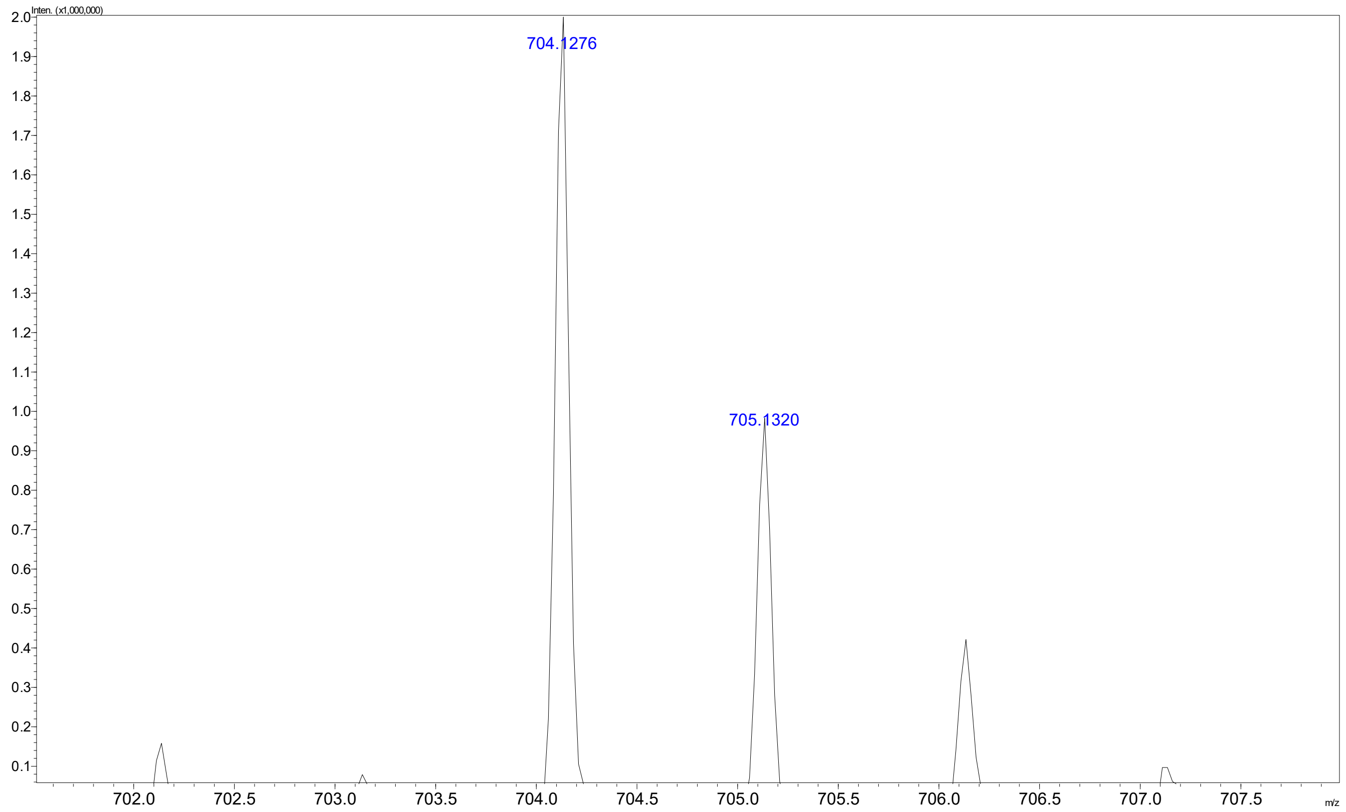


ESI-MS spectrum of compound **S19**.

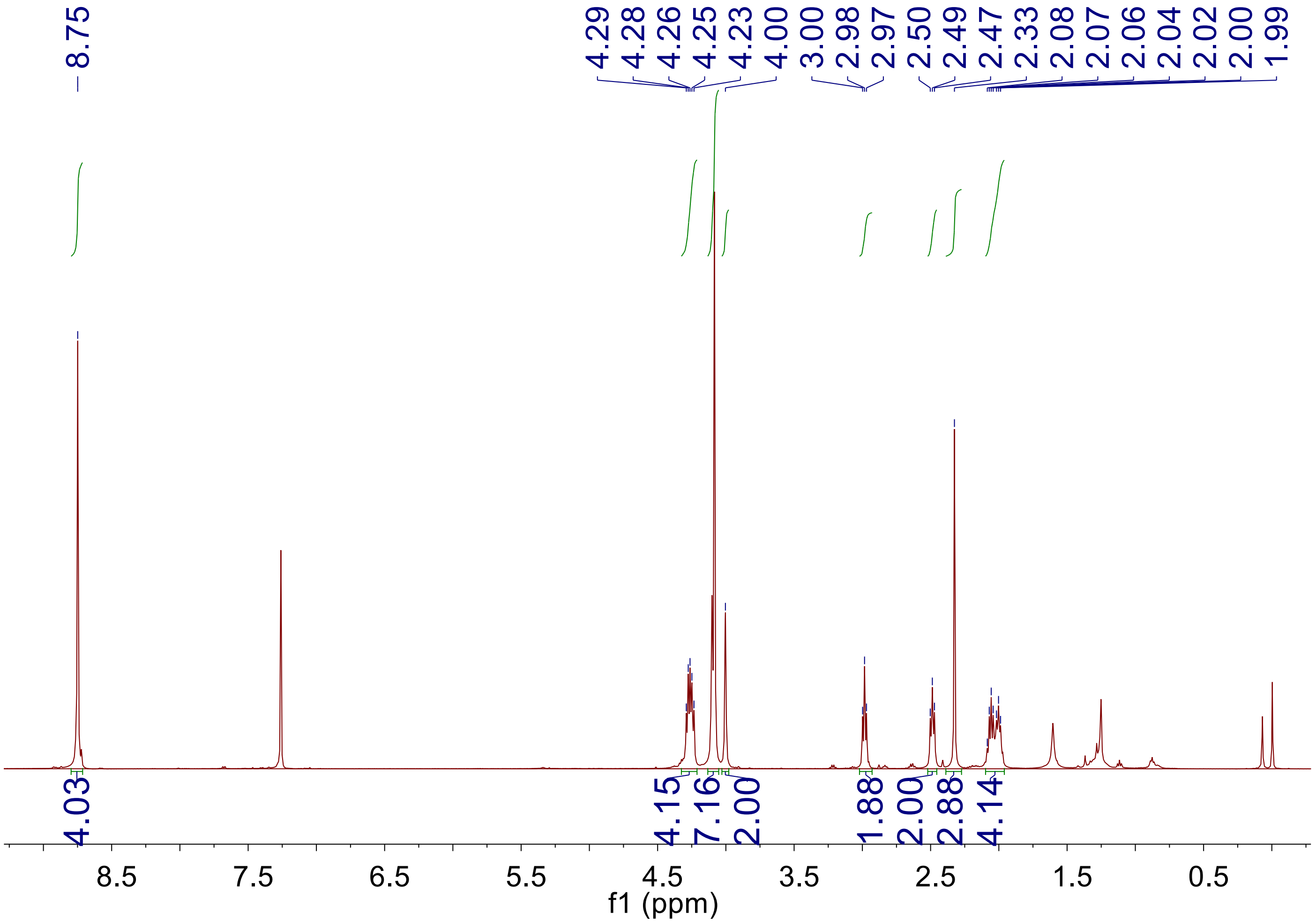


$^{1}$H NMR spectrum of compound **S20** in CDCl3.

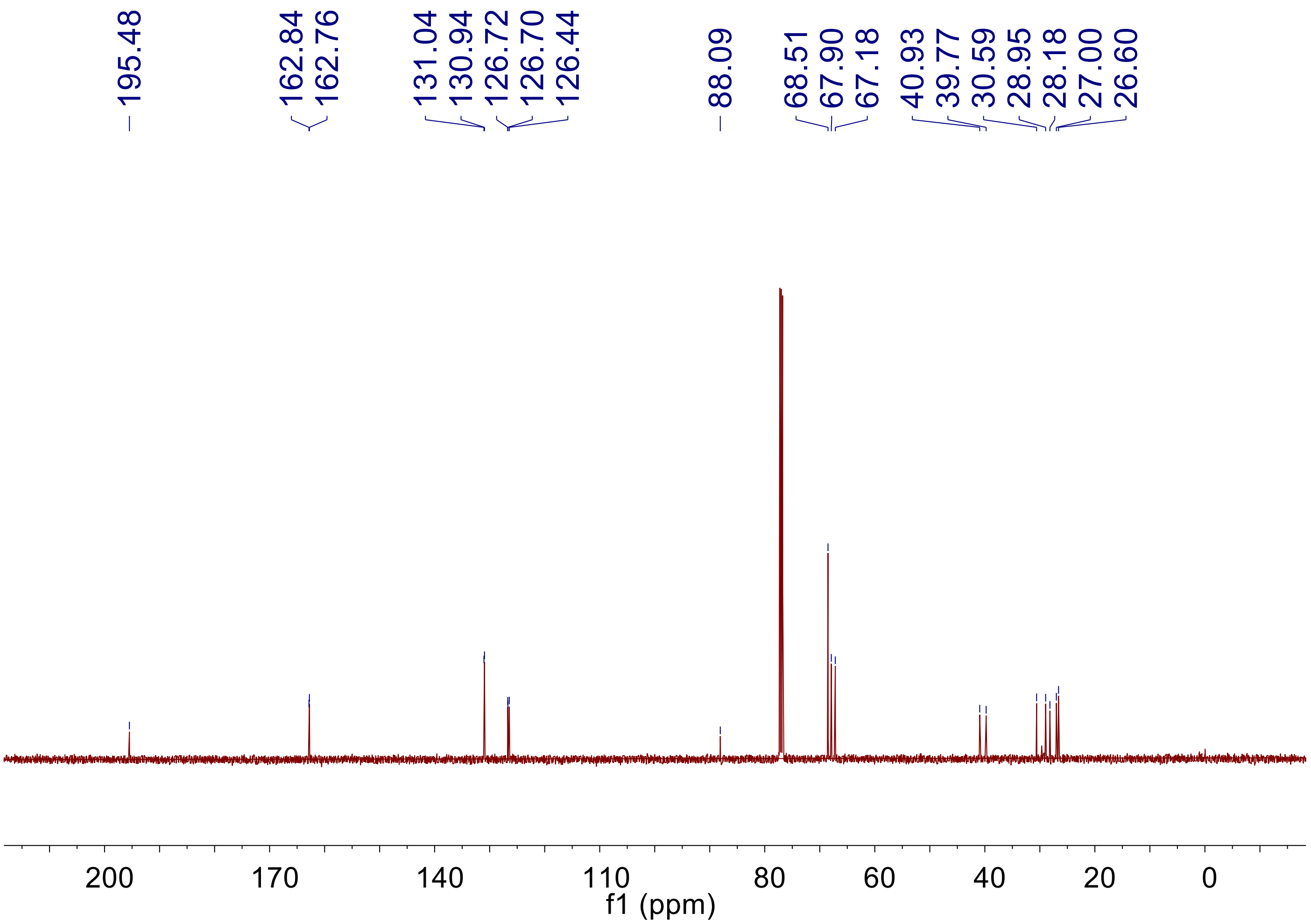


$^{13}$C NMR spectrum of compound **S20** in CDCl3.

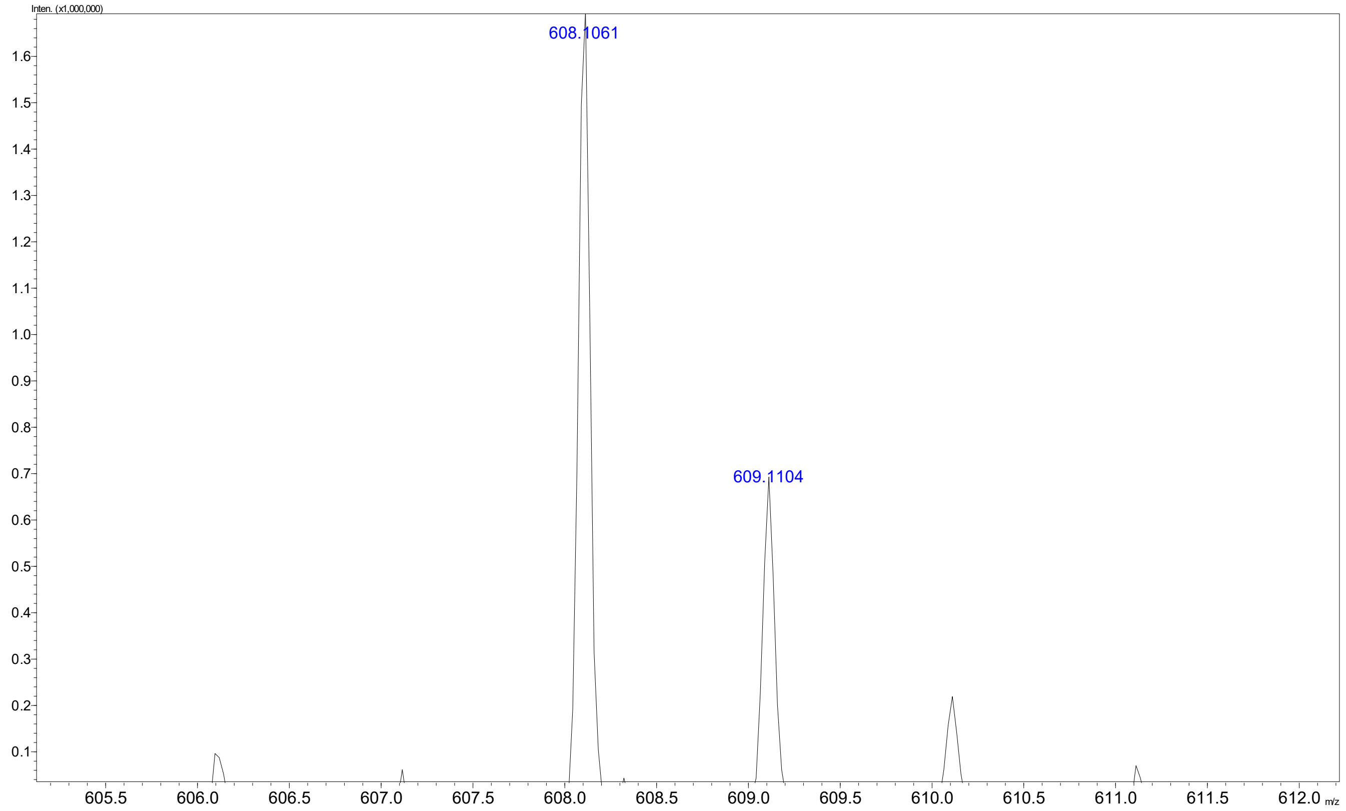


ESI-MS spectrum of compound **S20**.

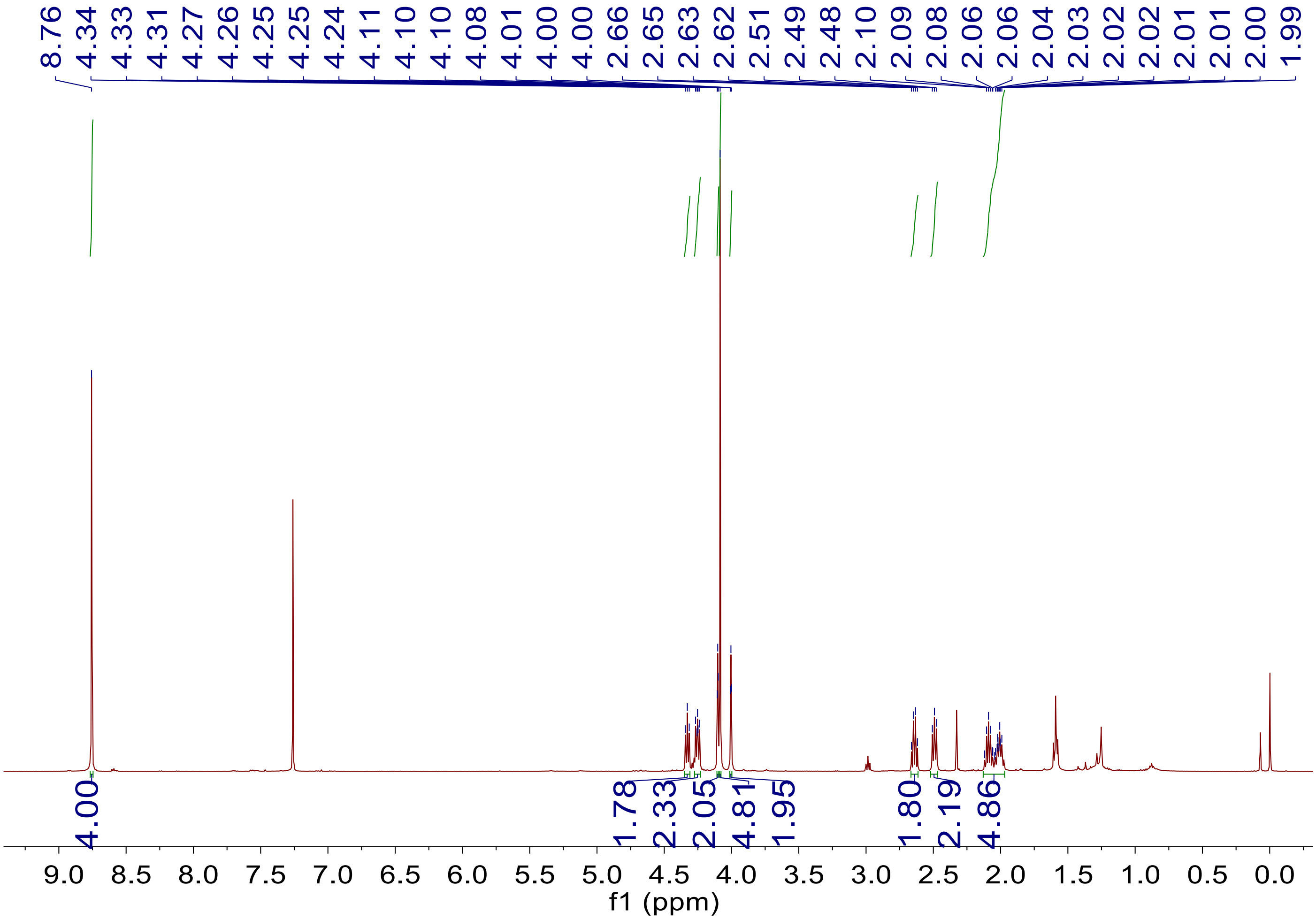


$^{1}$H NMR spectrum of compound **S21** in CDCl3.

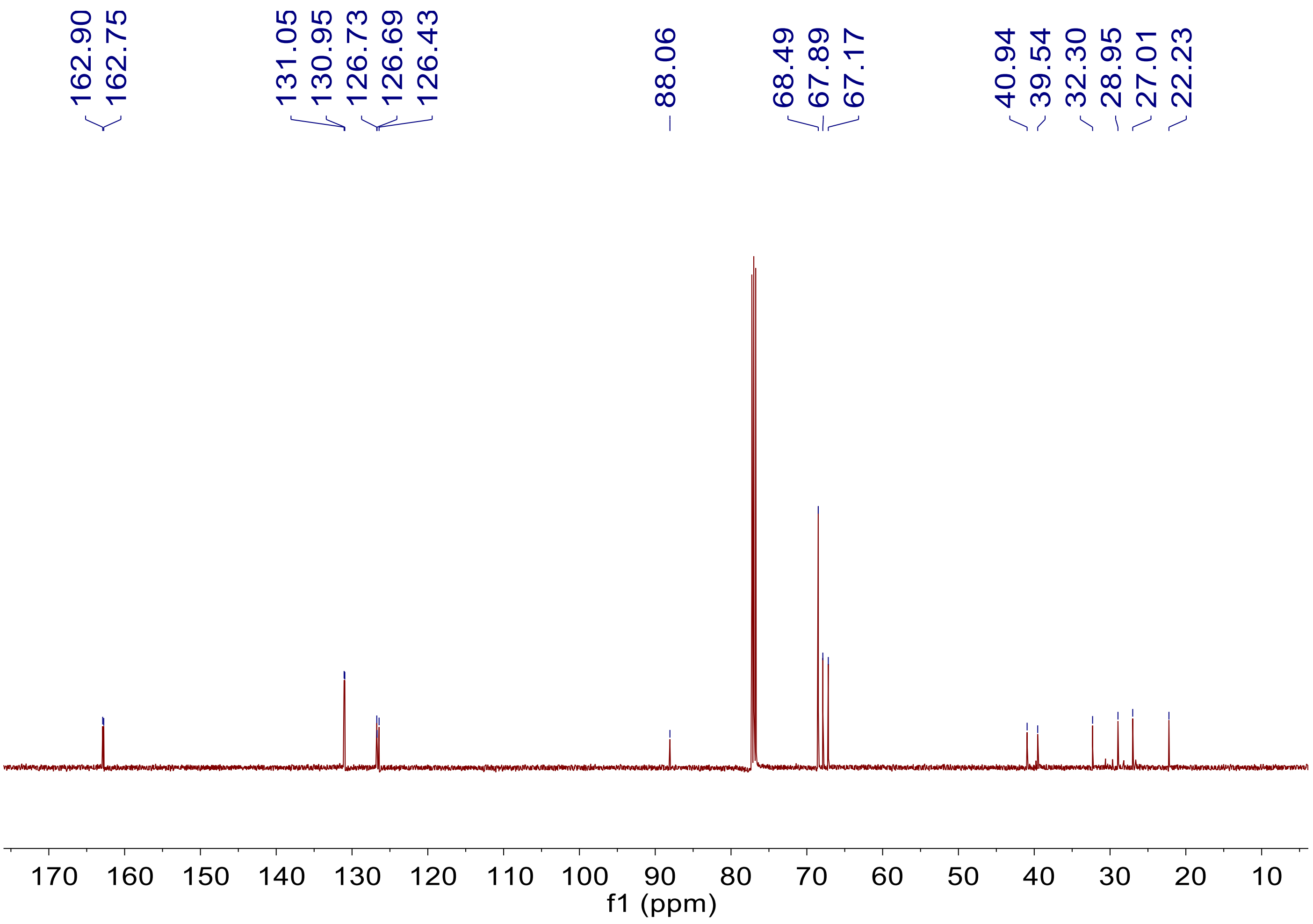


$^{13}$C NMR spectrum of compound **S21** in CDCl3.

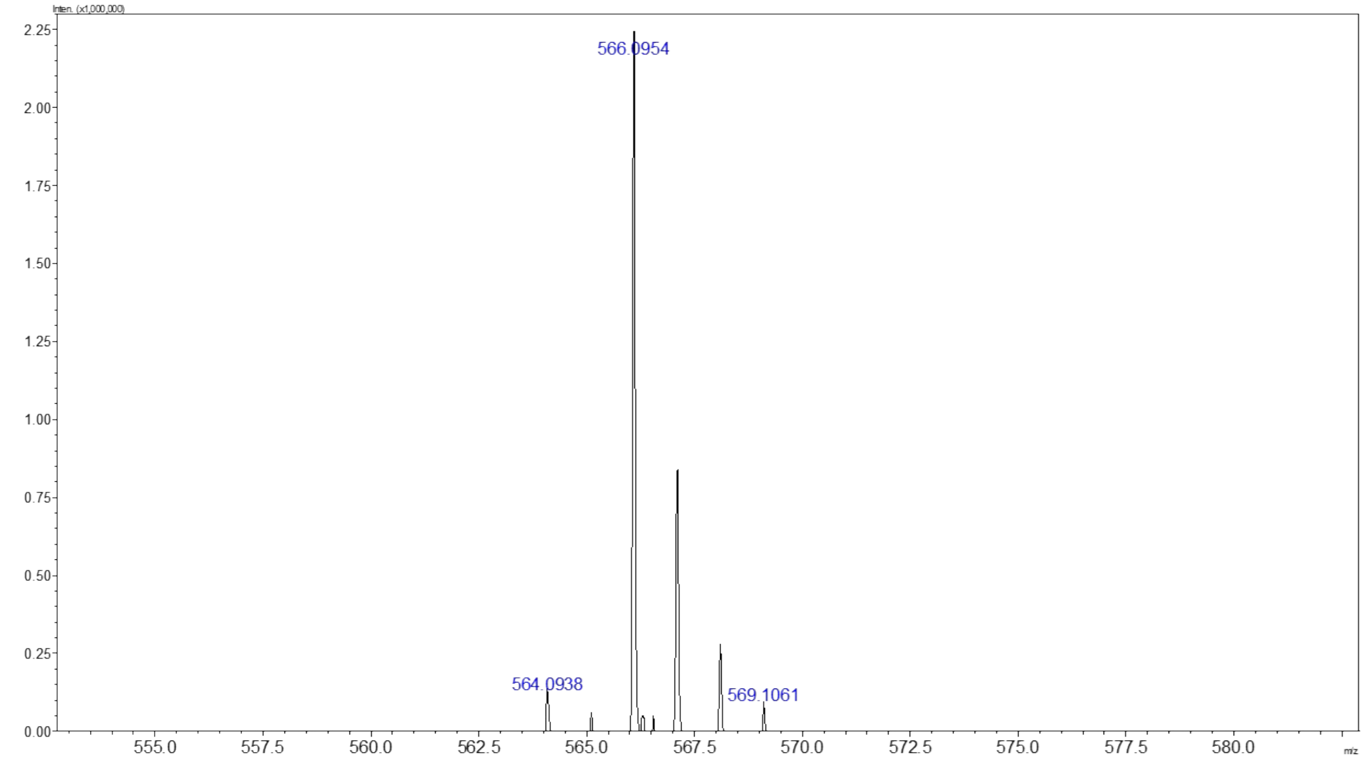


ESI-MS spectrum of compound **S21**.

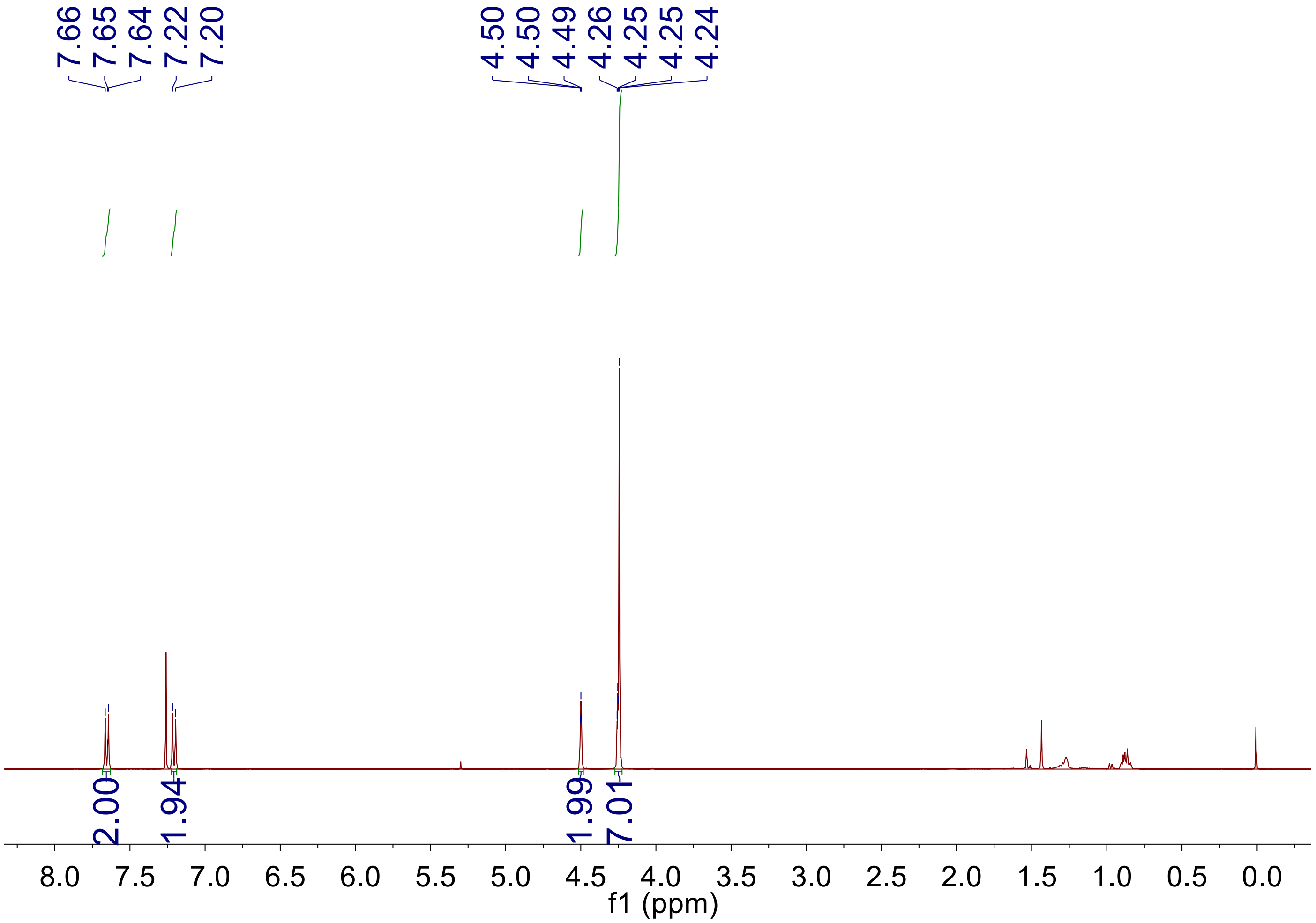


$^{1}$H NMR spectrum of compound **S22** in CDCl3.

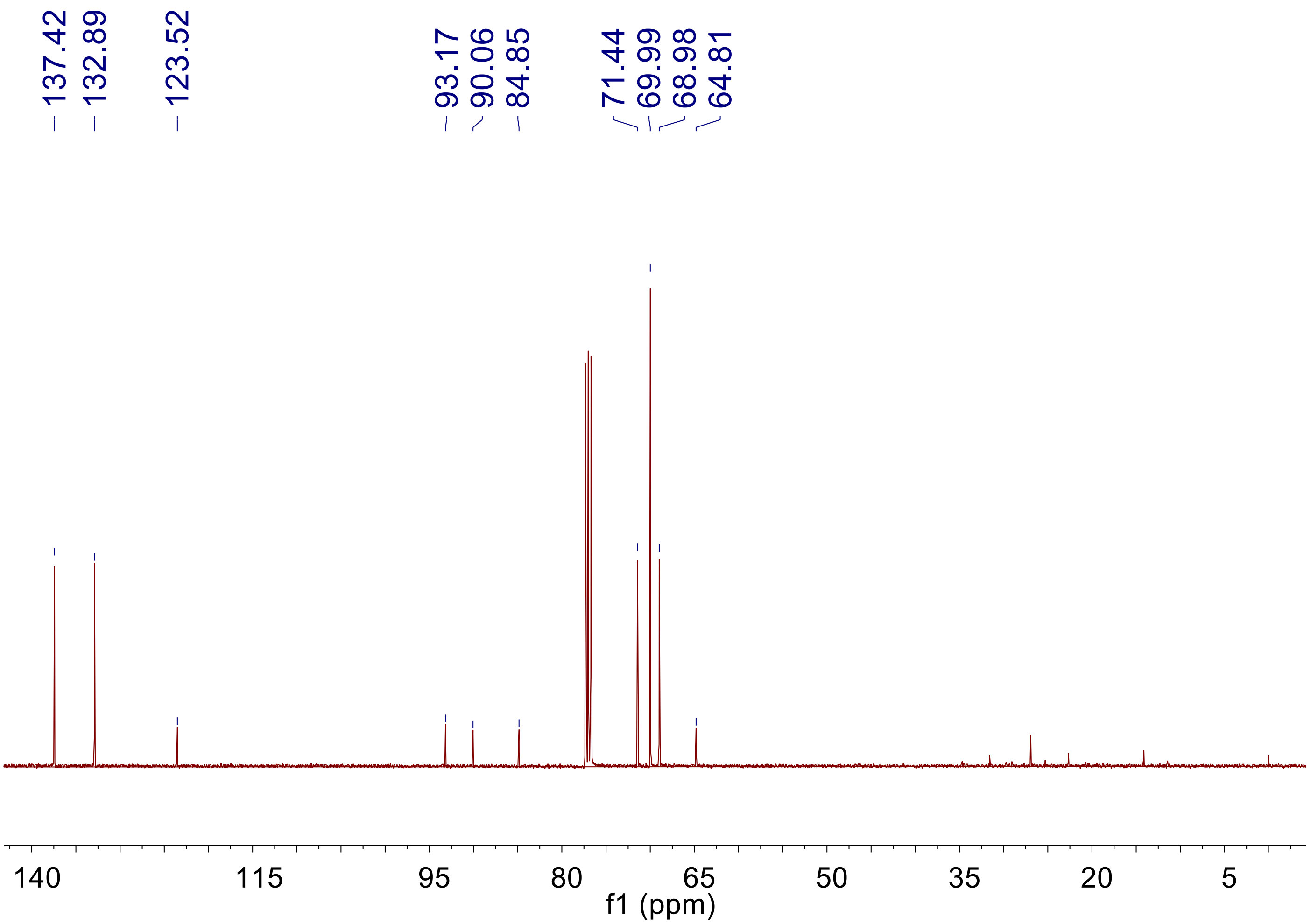


$^{13}$C NMR spectrum of compound **S22** in CDCl3.

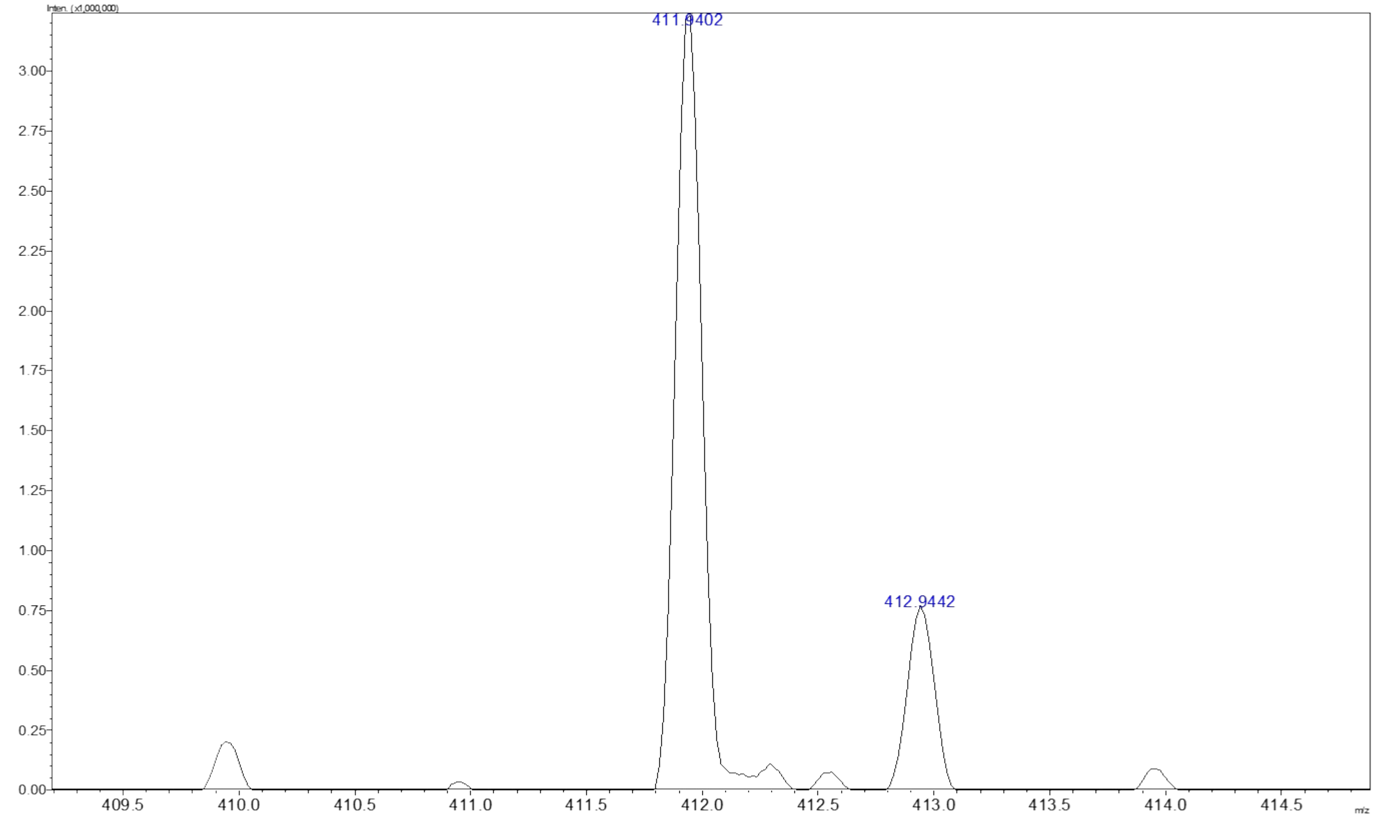


ESI-MS spectrum of compound **S22**.

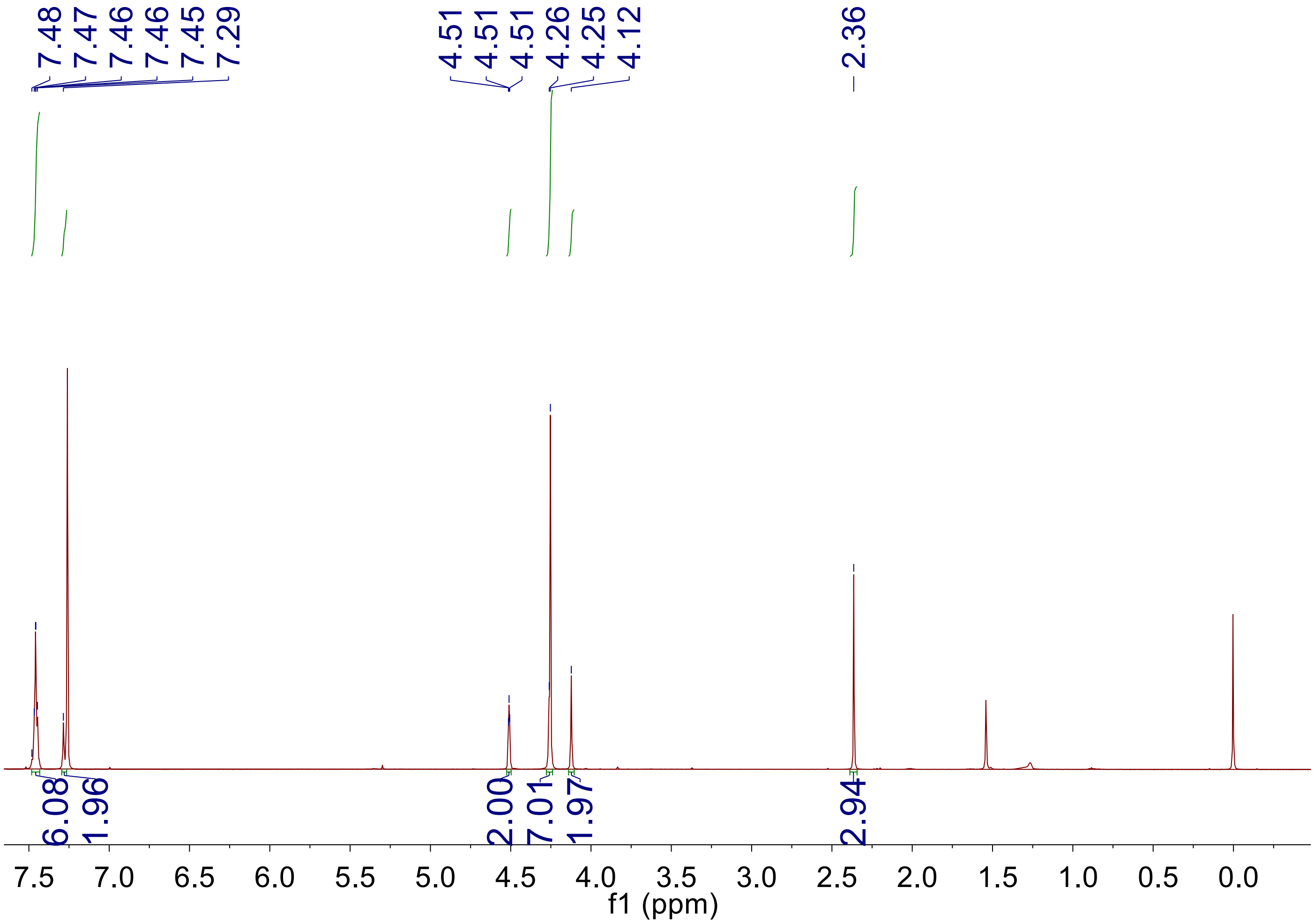


$^{1}$H NMR spectrum of compound **S23** in CDCl3.

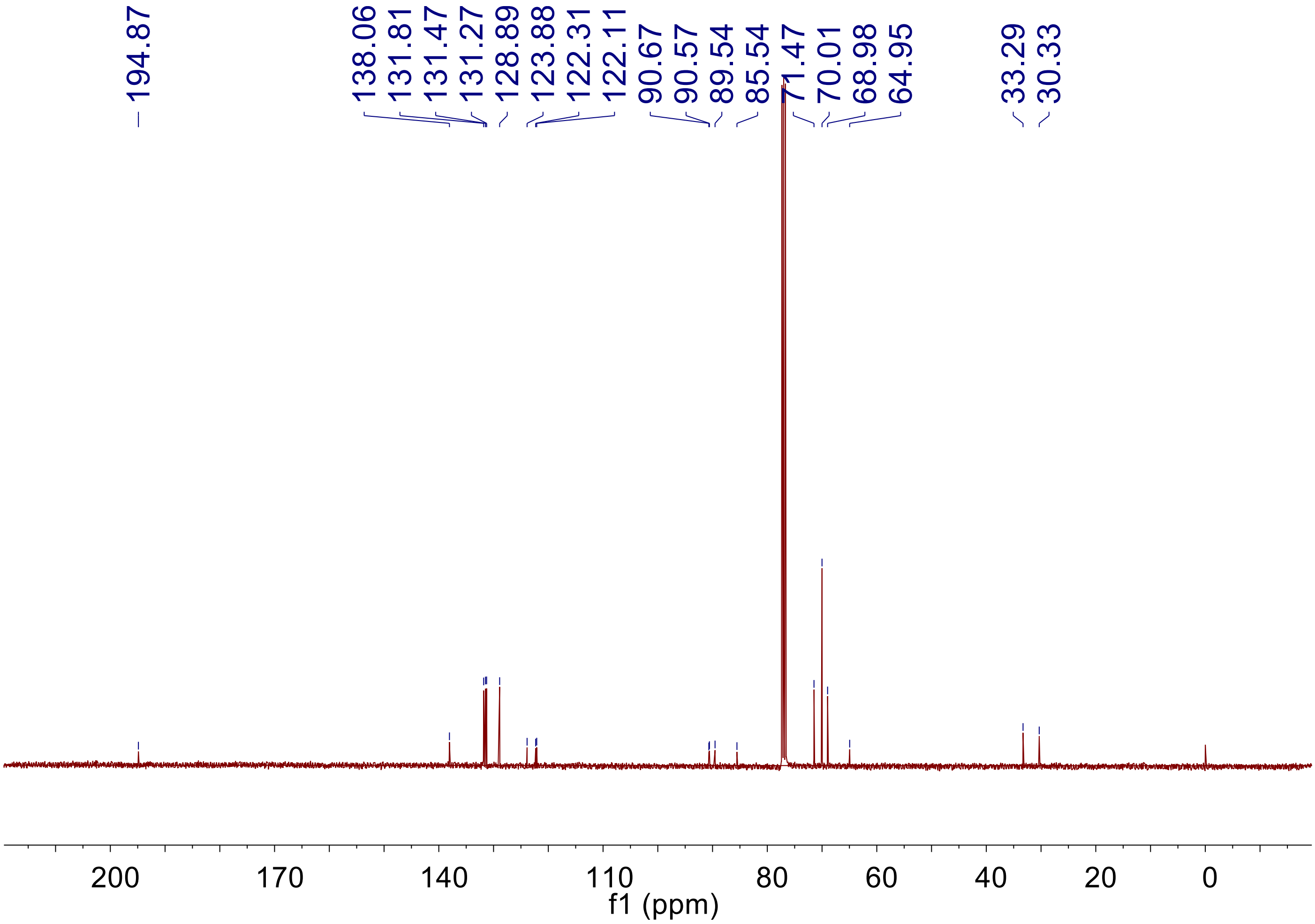


$^{13}$C NMR spectrum of compound **S23** in CDCl3.

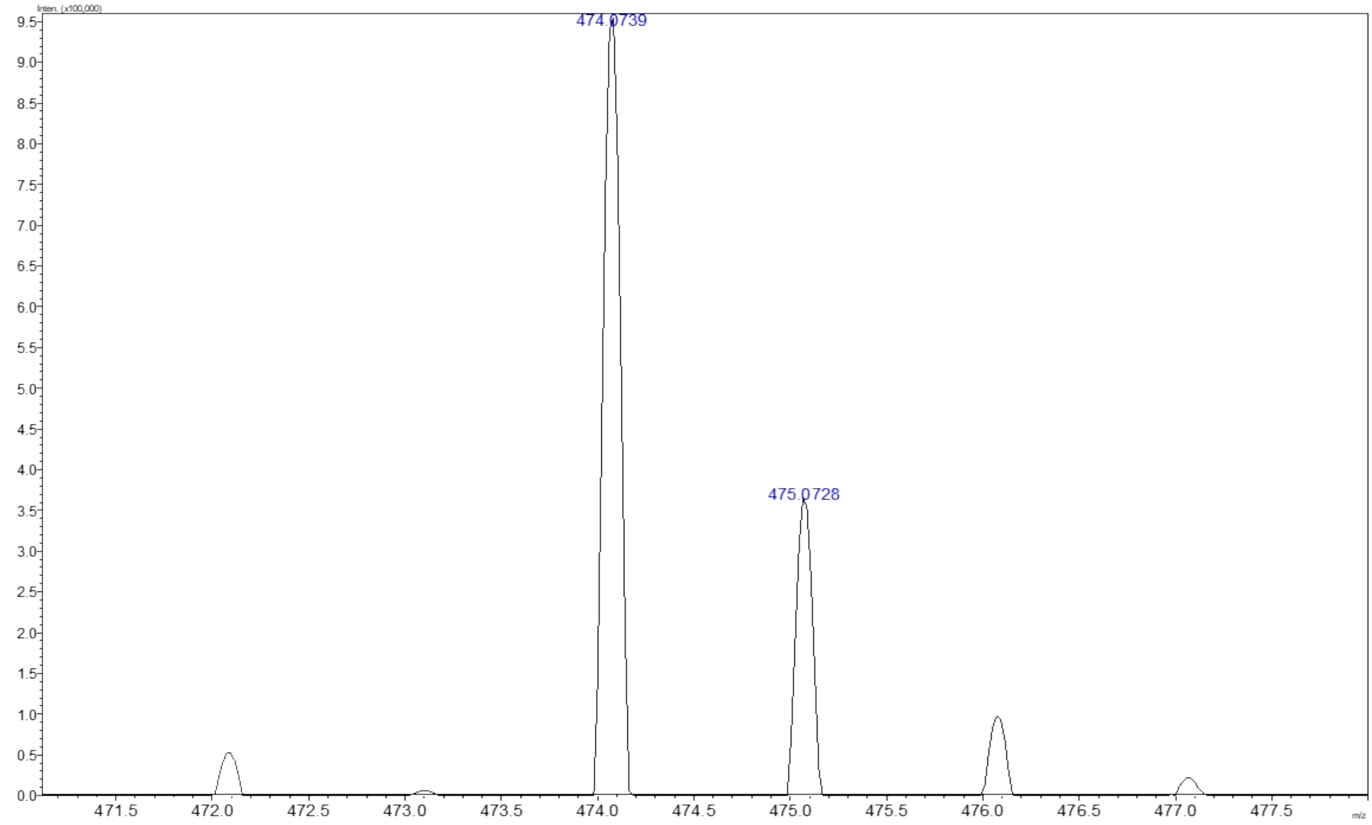


ESI-MS spectrum of compound **S23**.

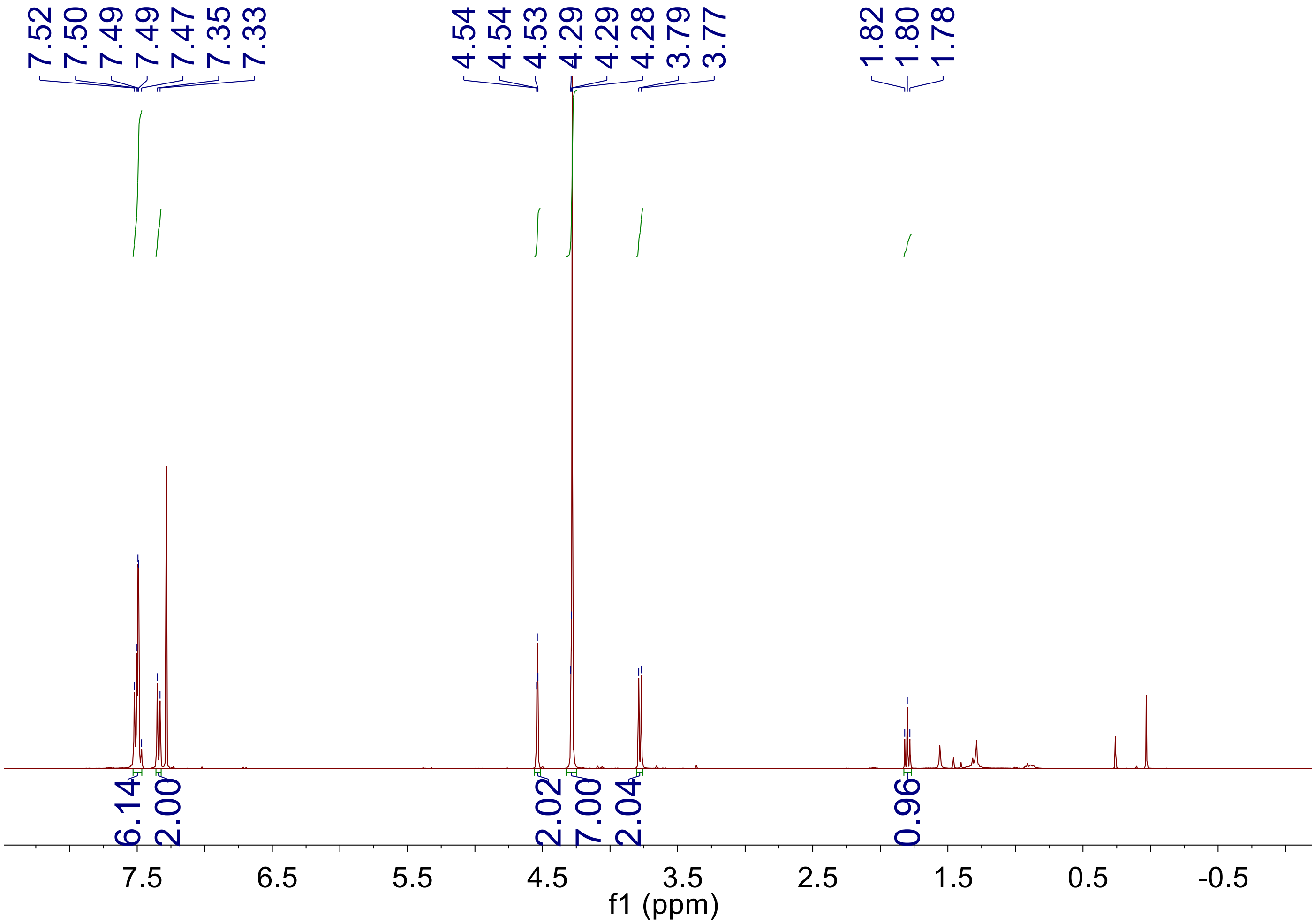


$^{1}$H NMR spectrum of compound **S24** in CDCl3.

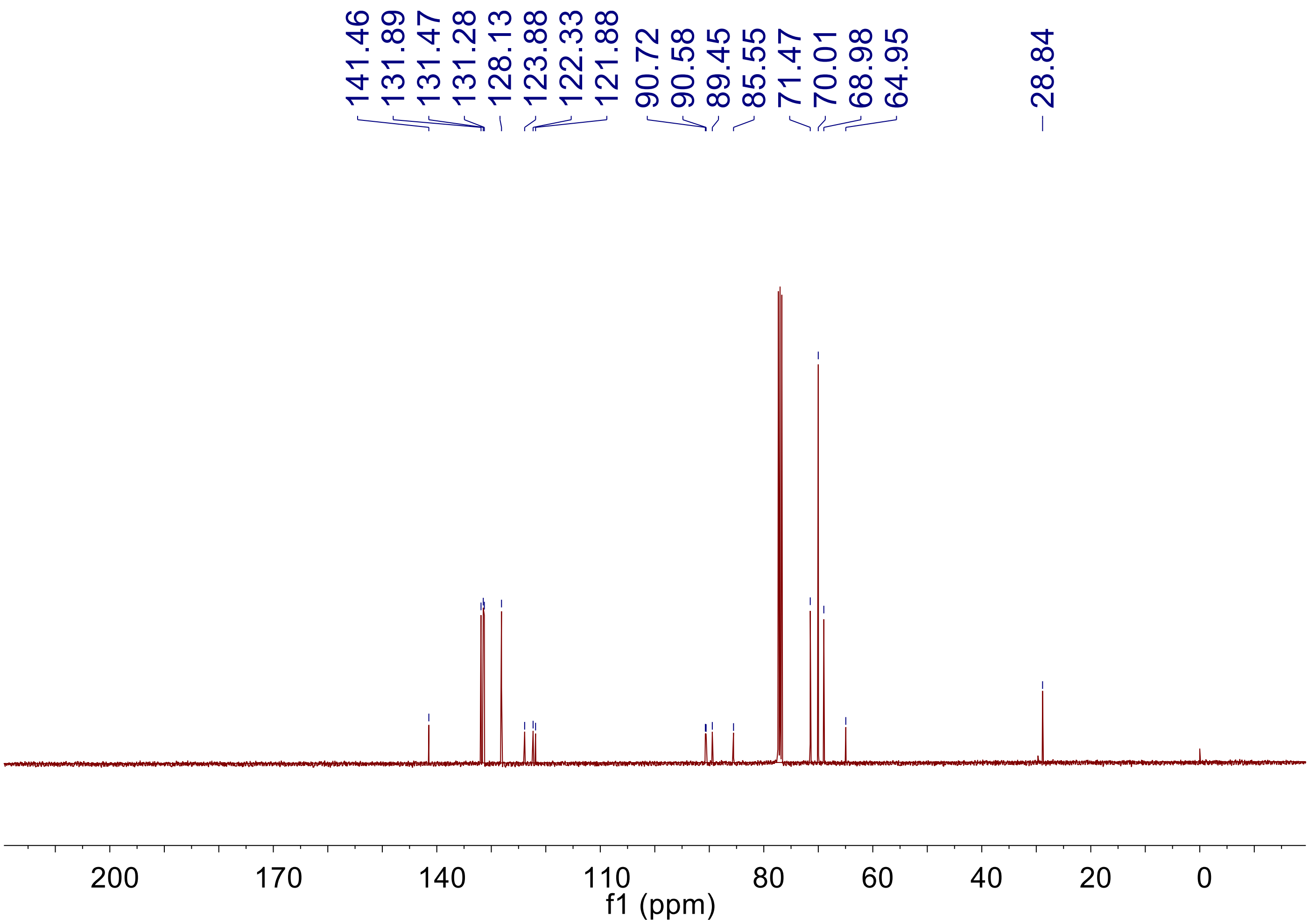


$^{13}$C NMR spectrum of compound **S24** in CDCl3.

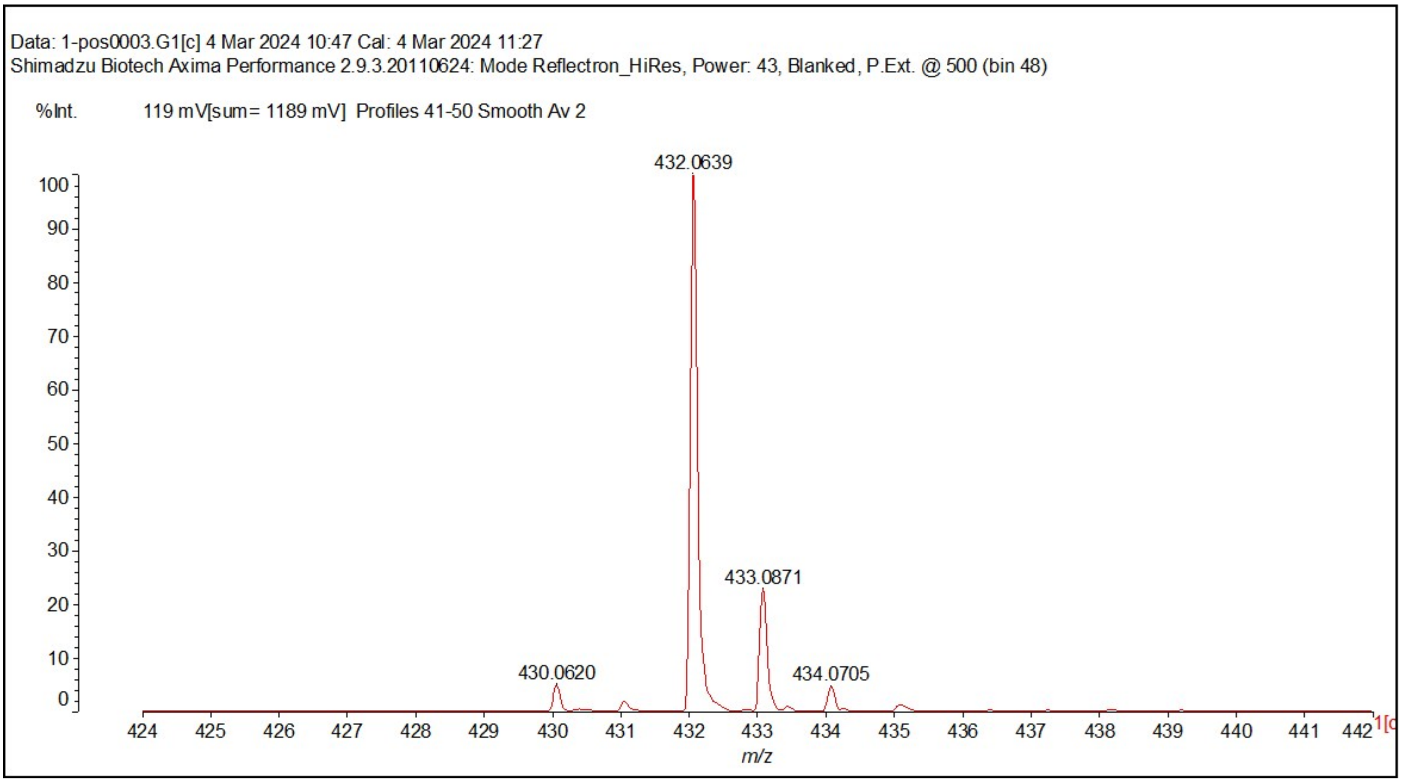


MS(MALDI-TOF) spectrum of compound **S24**.

## 3. Bottom electrode fabrication and preparation of the SAMs

The procedures of fabrication of template-stripped gold ($Au^{TS}$) are described in previous literature.[1] We deposited the first 50.0 nm at a rate of 0.2 Å/s and the following 150.0 nm at a rate of 1.0 Å/s. Glass slides (5 mm × 5 mm) are adhered to gold substrate with adhesive (353ND, Epoxy Technology Inc) and cured under 80 °C for 12 h. After curing, the slide was cleaved off with a razor blade. The fabrication of flexible electrodes followed the aforementioned methods. Polyethylene terephthalate (PET) films, with rectangular shape of 75 mm length and 8 mm width, are adhered to gold substrate with optical adhesive (Norland, No. 61). The flexible substrates were solidified at 365nm UV light for 24 h and peeled off by razor blade. The directly deposited Au bottom electrode ($Au^{DE}$) was firstly deposited on wafer with 10 nm Cr, followed by 50 nm Au at a rate of 1.0 Å/s. The surface topography of bottom electrode was imaged by Bruker Icon with ScanAsyst mode. Figure S1 shows the AFM images of $Au^{TS}$ with rms roughness of 0.4 nm and $Au^{DE}$ with rms roughness of 1.0 nm.

The SAMs were prepared in a glove box to minimize the contaminations from air. About 1.0 mg of pure compound was added into a vial, following the addition of 1.0 ml dry dichloromethane (DCM). After the compound dissolved, dry ethanol (9.0 ml) was then added to dilute the concentration to ~ 0.1 mmol/L of SAM solution. The solution was filtered and a freshly template-stripped metal substrate was immersed into it. The SAMs were formed for 12 hours under the atmosphere of $N_2$. The substrate was rinsed thoroughly with DCM, then ethanol, and finally dried under a stream of nitrogen.

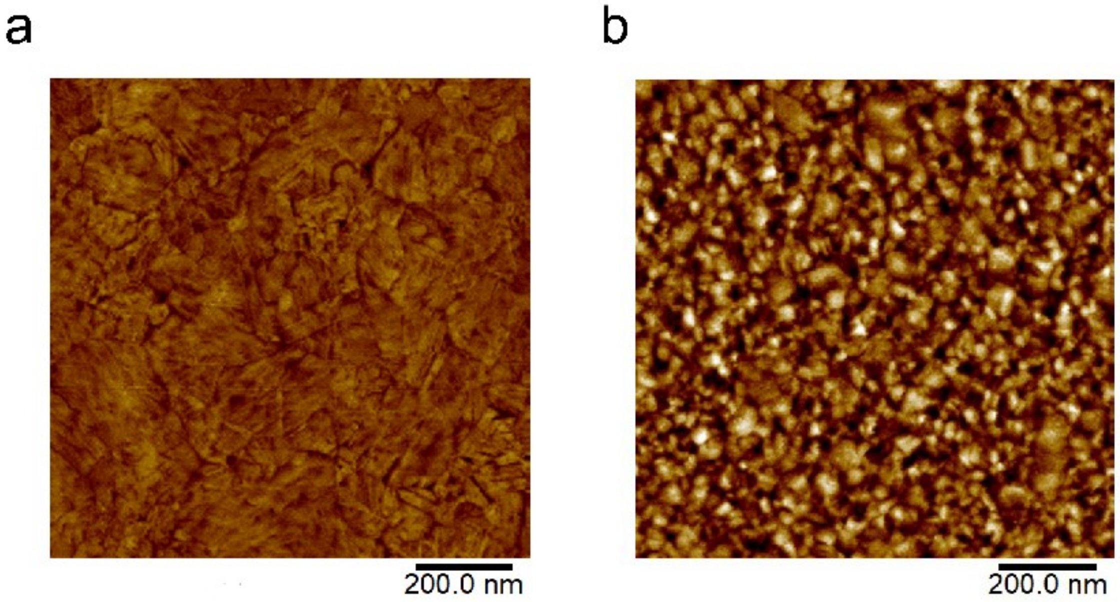


**Figure S1.** AFM images of the a) $Au^{TS}$ and b) $Au^{DE}$ surfaces with scale bar of 200 nm.

## 4. Electrochemistry

### 4.1 Electrochemical Characterization of the SAMs

The CV measurements of Fc-based SAMs on $Au^{TS}$ electrodes were carried out with a three-electrode system (platinum as counter the electrode, Ag/AgCl as reference electrode, and gold bottom electrode as the working electrode) and with the aqueous solution of 1.0 mol/L $HClO_4$ as the electrolyte, to record the redox behavior of Fc termini in SAMs.[2] The values of the surface coverage of Fc-terminated SAMs were also determined by the CV data. Figure S2 shows cyclic voltammograms of SAMs on $Au^{TS}$ electrodes with a three-electrode system (platinum as counter the electrode, Ag/AgCl as reference electrode, and gold bottom electrode as the working electrode) with aqueous 1.0 mol/L $HClO_4$ as electrolyte. We performed the cyclic voltammograms (CVs) between -0.1 to 0.9 V at a scan rate of 1.0 V/s.

Table S1 compiles peak anodic potential ($E_{pa}$), peak cathodic potential ($E_{pc}$), peak separation ($\Delta E_p$) and surface coverage of Ferrocene unit ($\Gamma_{Fc}$).

### 4.2 Electrochemical rearrangement of SAMs in aqueous solution

In order to verify the more ordered structures inside SAMs under electric field, we in situ performed CV measurements before and after the application of electric field. The Au electrode, as the working electrode, generate strong interface electric field, which in turn has potential to align the dipole at each molecule to parallel to the direction of electric field. We used 1.0 mol/L $Na_2SO_4$ as electrolyte and applied bias from +0.3 to -1.3 V. Specifically, we equipped the cell with $Na_2SO_4$ as electrolyte, followed by consecutive 100 sweeps from +0.3 to -1.3 V to modify the structure of SAMs. $Na_2SO_4$ was poured and the cell (together with the electrodes and SAMs) was rinsed with deionized water for 3 times to remove residual electrolyte. Finally, the CV measurements on the modified SAMs were carried out with $HClO_4$ as electrolyte to determine the electrochemical performance of SAMs. To exclude the effect of residue $Na_2SO_4$ on SAMs, we carried out

experiments (denoted as "blank"), in which $Na_2SO_4$ solution was poured into and out of the cell without applying bias, followed by the same procedure depicted above. The values of electrochemical parameters are compiled in Table S2. It suggests that molecules in SAMs are forced to be more ordered attributed to larger peak difference and higher surface coverage after enhancement. Furthermore, since the parameters of "blank" experiments remain unchanged, the effect of aqueous $Na_2SO_4$ on SAMs can be overlooked. It shows that the rearrangement of SAMs can be realized in both solid and aqueous state.

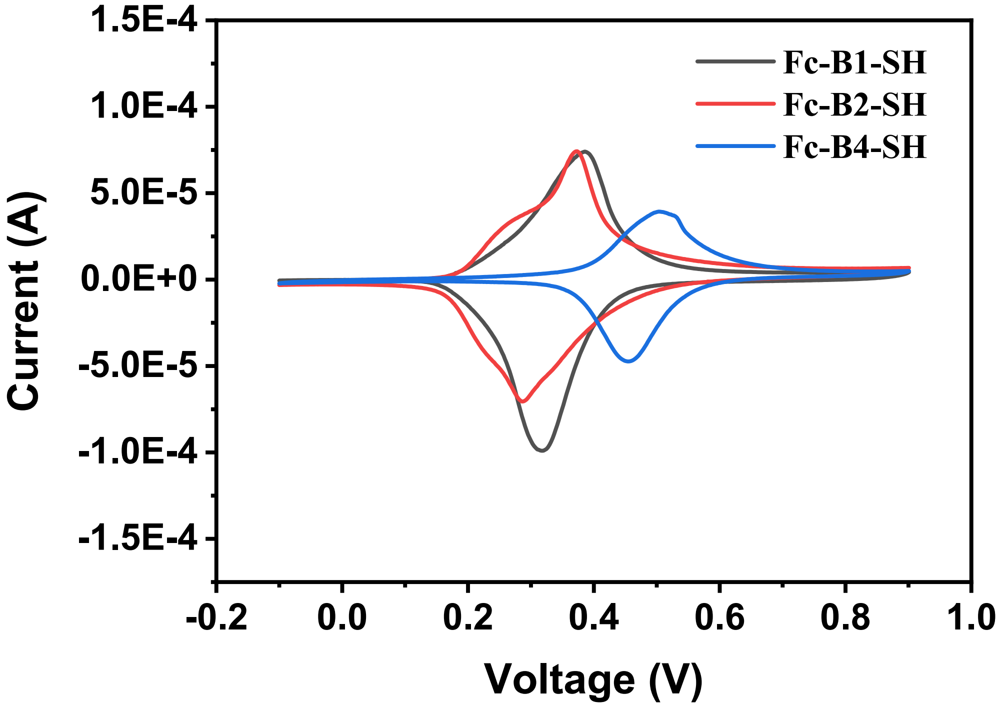


**Figure S2.** Cyclic voltammograms of SAMs of $Au^{TS}$-S-B-Fc, (B1, B2, B4) with aqueous 0.1 mol/L $HClO_4$ as electrolyte solution and Ag/AgCl as a reference electrode recorded at a scan rate of 1.0 V/s.

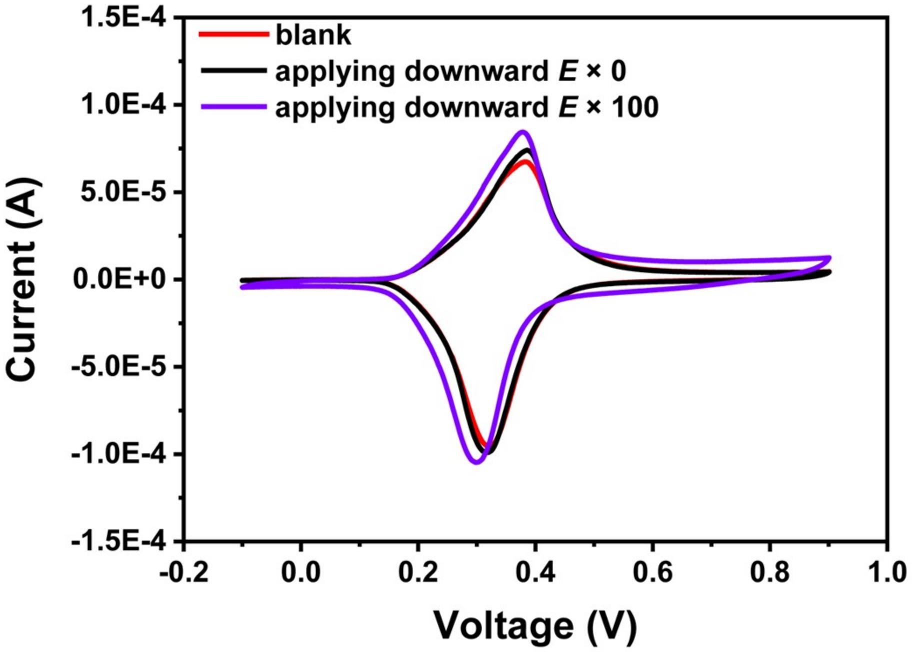


**Figure S3.** Cyclic voltammograms of SAMs of $Au^{TS}$-S-B1-Fc with aqueous 0.1 mol/L $HClO_4$ as electrolyte solution and Ag/AgCl as a reference electrode recorded at a scan rate of 1.0 V/s. Rearrangement process was carried out in 1.0 mol/L aqueous $Na_2SO_4$.

**Table S1. Peak anodic potential ($E_{pa}$), peak cathodic potential ($E_{pc}$), peak separation ($\Delta E_p$) and surface coverage of SAMs with different backbone (B1, B2, B4).**

| Molecule | $E_{pa}$ (mV) | $E_{pc}$ (mV) | $\Delta E_p$ (mV) | $\Gamma_{Fc}$ ($\times10^{-10}$ mol cm$^{-2}$) |
|---|---|---|---|---|
| Fc-B1-SH | 381 ± 9 | 317 ± 7 | 64 ± 13 | 3.79 ± 0.29 |
| Fc-B2-SH | 372 ± 2 | 287 ± 1 | 84 ± 4 | 2.97 ± 0.22 |
| Fc-B4-SH | 507 ± 5 | 460 ± 8 | 47 ± 3 | 1.60 ± 0.28 |

**Table S2. Peak anodic potential ($E_{pa}$), peak cathodic potential ($E_{pc}$), peak separation ($\Delta E_p$) and surface coverage for $Au^{TS}$-S-B1-Fc before and after applying electric field in aqueous solution.**

| State | $E_{pa}$ (mV) | $E_{pc}$ (mV) | $\Delta E_p$ (mV) | $\Gamma_{Fc}$ ($\times10^{-10}$ mol cm$^{-2}$) |
|---|---|---|---|---|
| blank | 384 ± 3 | 321 ± 3 | 63 ± 1 | 3.71 ± 0.08 |
| before rearrangement | 381 ± 9 | 317 ± 7 | 64 ± 13 | 3.79 ± 0.29 |
| after rearrangement | 379 ± 9 | 300 ± 2 | 79 ± 10 | 4.29 ± 0.17 |

## 5. Fabrication and measurement of the molecular junctions.

### 5.1 Junction results under continuously changed electric field *E*.

We use cone-shaped tips of $Ga_2O_3$/EGaIn as top electrodes to form ohmic and non-invasive contact with the SAMs following previous research. [3, 4] Top electrode was biased while bottom Au electrode was grounded. A Keithley 6430 source meter was used to apply voltage and LabView was used to control the Keithley and collect the data. As for *J*(V) measurements, the bias sequence followed 0 → -1.8 → +1.8 → 0 V. At least 150 sweeps were conducted on an individual junction, in order to investigate the trend of rectification ratio. The values of log|*J*(V)| and log(RR) were determined by taking arithmetic mean and standard deviation of data sets since the number of sweeps were not sufficient for Gaussian fitting. All the error bars for traces of $Au^{TS}$-S-B-Fc//$Ga_2O_3$/EGaIn junctions are available in Figure S4-7. Enhancement of rectification ratio can also be observed when bottom electrode is $Pt^{TS}$, indicating that enhancement derives from structure of molecules rather than bottom electrode (Fig.S8). When expanding the length of alkyl chain in molecules, the rectification ratio can still be enhanced.

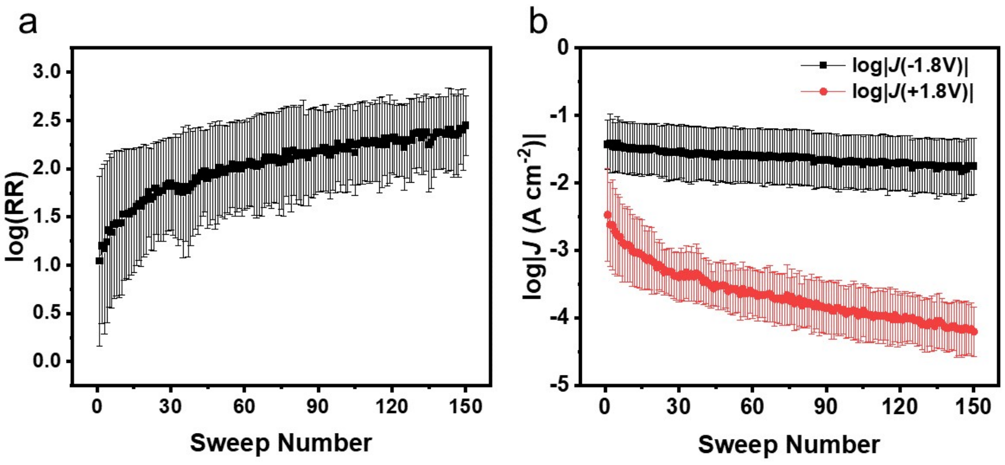


**Figure S4.** a) Average log(RR) and b) log|*J*(±1.8 V)| of $Au^{TS}$-S-B1-Fc//$Ga_2O_3$/EGaIn junctions.

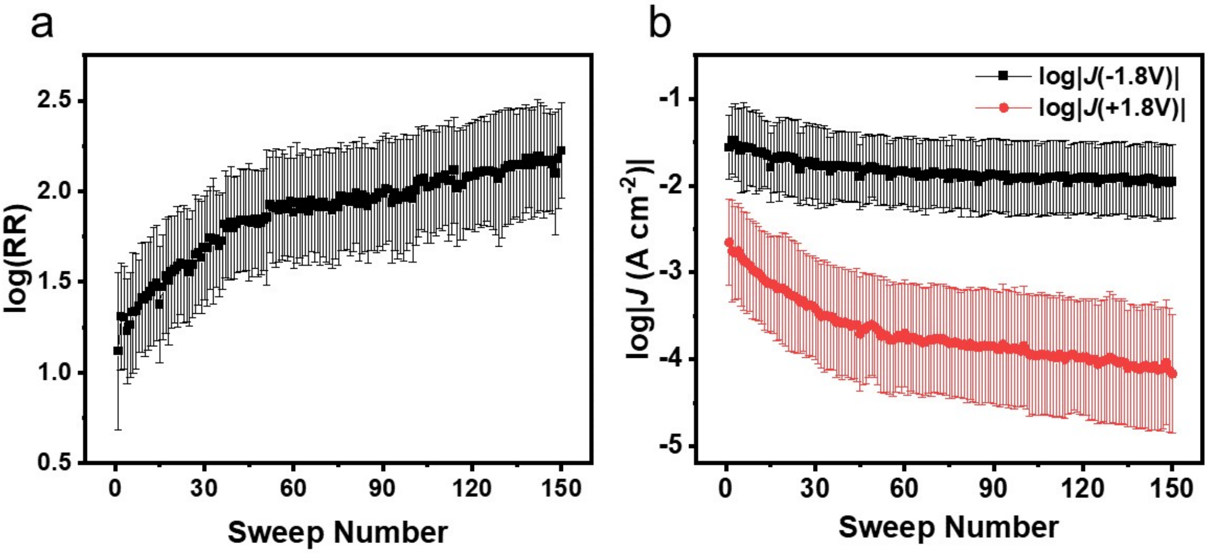


**Figure S5.** a) Average log(RR) and b) log|$J$(±1.8 V)| of Au$^{TS}$-S-B2-Fc//$Ga_2O_3$/EGaIn junctions.

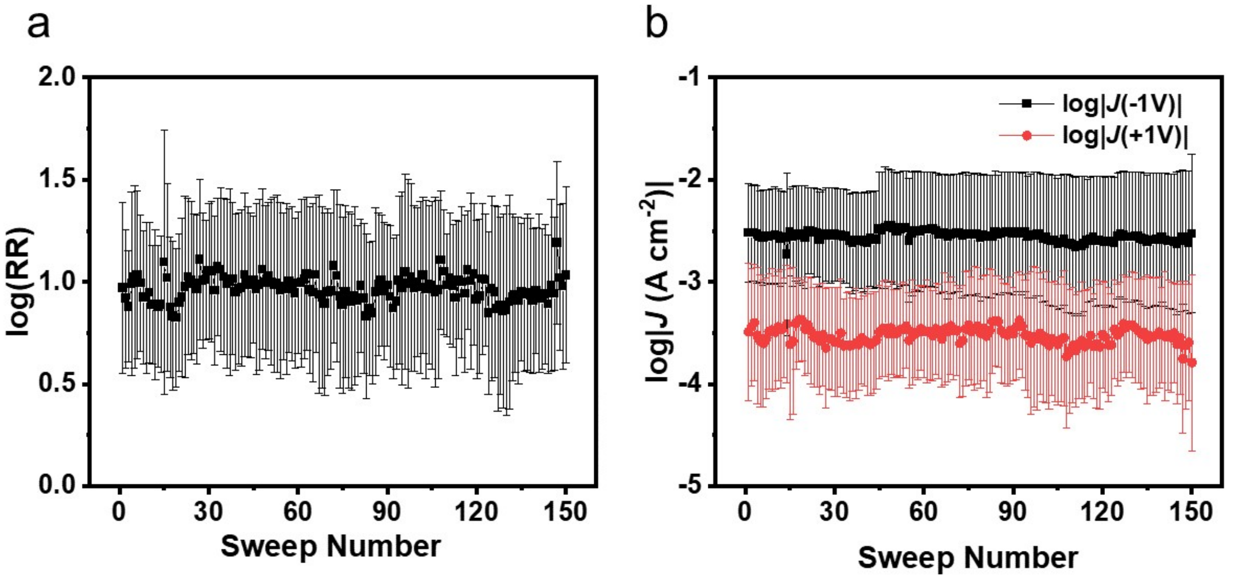


**Figure S6.** a) Average log(RR) and b) log|$J$(±1.8 V)| of Au$^{TS}$-S-B3-Fc//$Ga_2O_3$/EGaIn junctions.

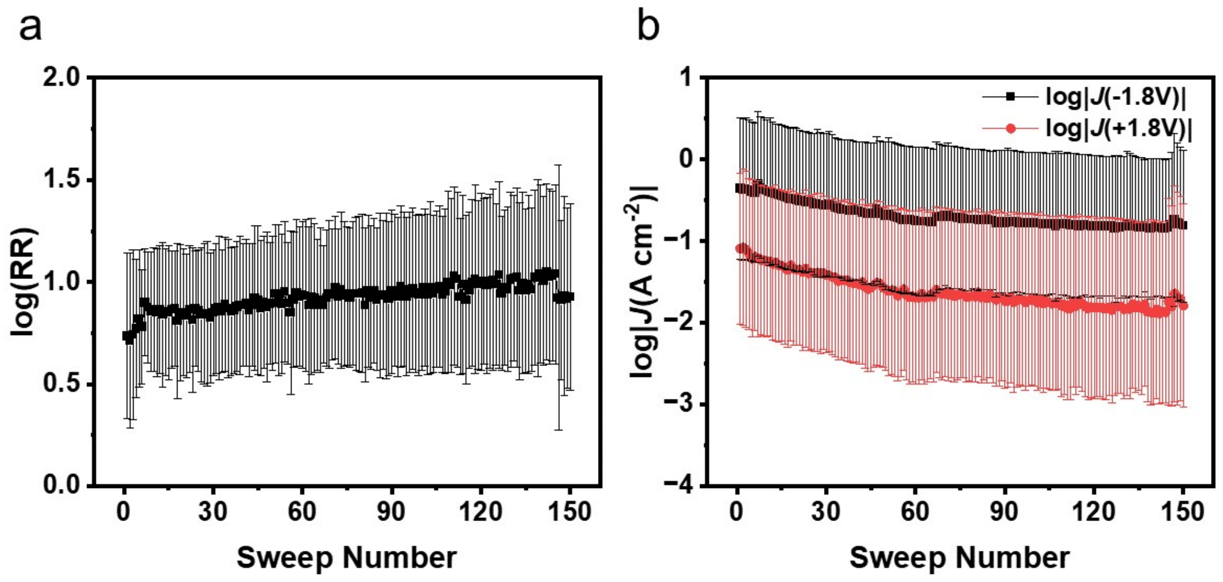


**Figure S7.** a) Average log(RR) and b) log|$J$(±1.8 V)| of Au$^{TS}$-S-B4-Fc//$Ga_2O_3$/EGaIn junctions.

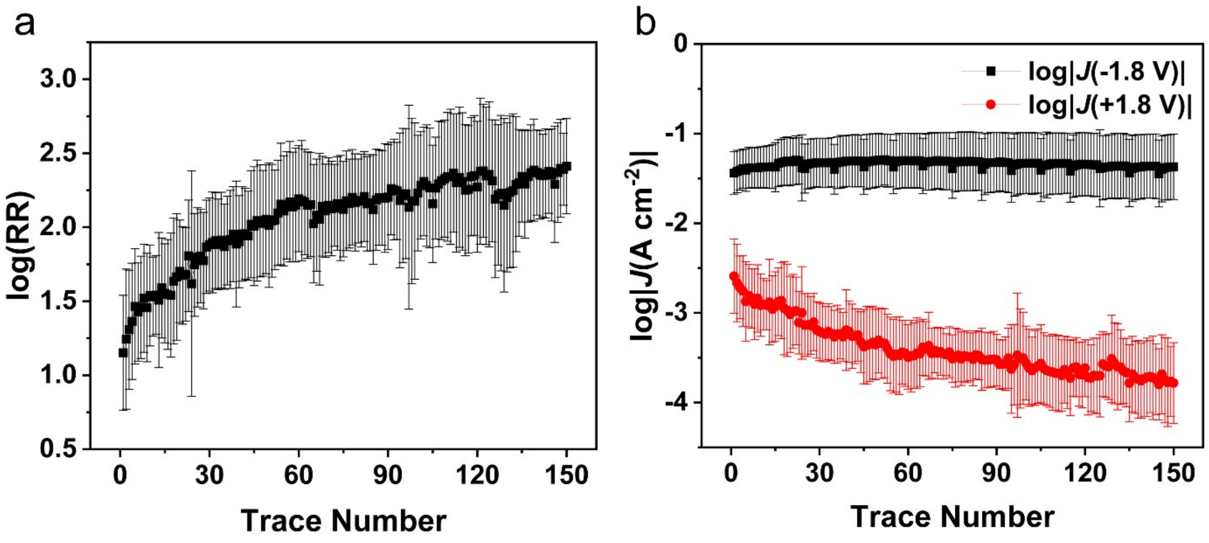


**Figure S8.** a) Average log(RR) and b) log|$J$(±1.8 V)| of Pt$^{TS}$-S-B1-Fc//$Ga_2O_3$/EGaIn junctions.

### 5.2 Light emission experiments

We performed light emission experiments on the $Au^{TS}$-S-B1-Fc//$Ga_2O_3$/EGaIn junctions following previously reported procedures. [5] Glass slides (thickness = 150 μm) were adhered to 30 nm thick gold substrate with optical adhesive to produce semi-transparent $Au^{TS}$ bottom electrode and collect excited plasmons. The testbed of light emission experiments is shown in Figure S9. Optical characterization of the molecular junctions was performed using a wide-field inverted optical microscope (Nikon Eclipse Ti-E) with an air objective (magnification ×60) with an Andor electron-multiplying charge coupled device (iXon Ultra 897). We used 30 seconds integration time to record the light emission images at -1.8 V with a 300 EM Gain.

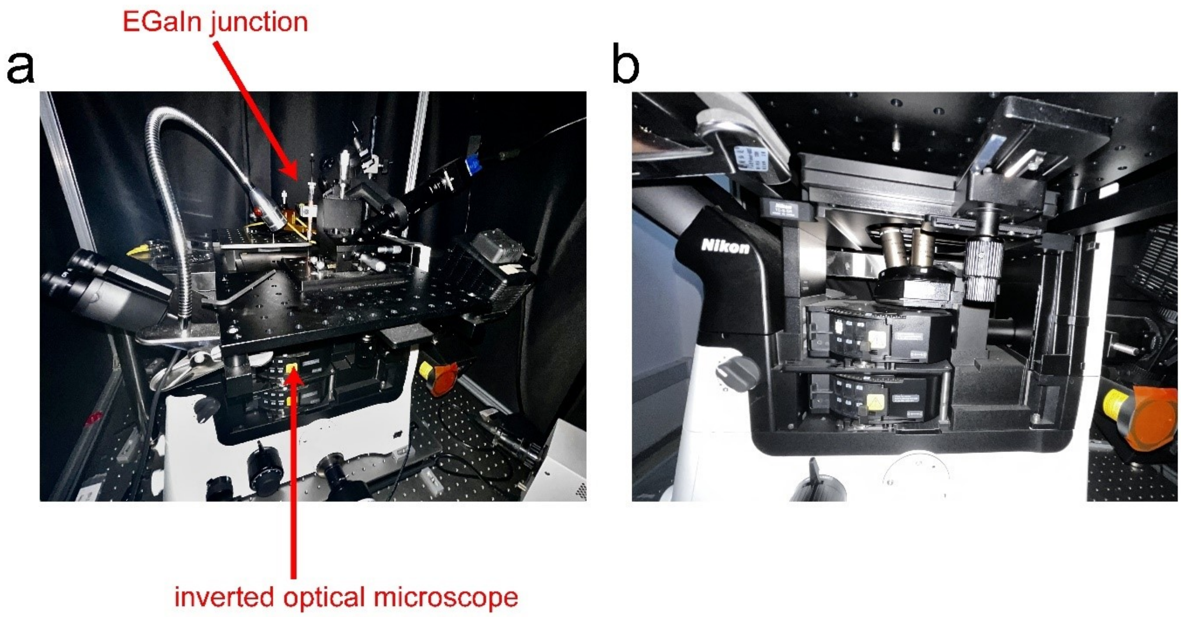


**Figure S9.** a) Setups for light emission capture of molecular junctions. The EGaIn in a glass syringe is manipulated with a micromanipulator. b) Inverted optical microscope was used to capture the footprint of EGaIn tip on SAMs.

### 5.3 Bending testbeds

We used mechanically bent electrodes to construct bending testbed. The home-built equipment is illustrated in Figure S10.

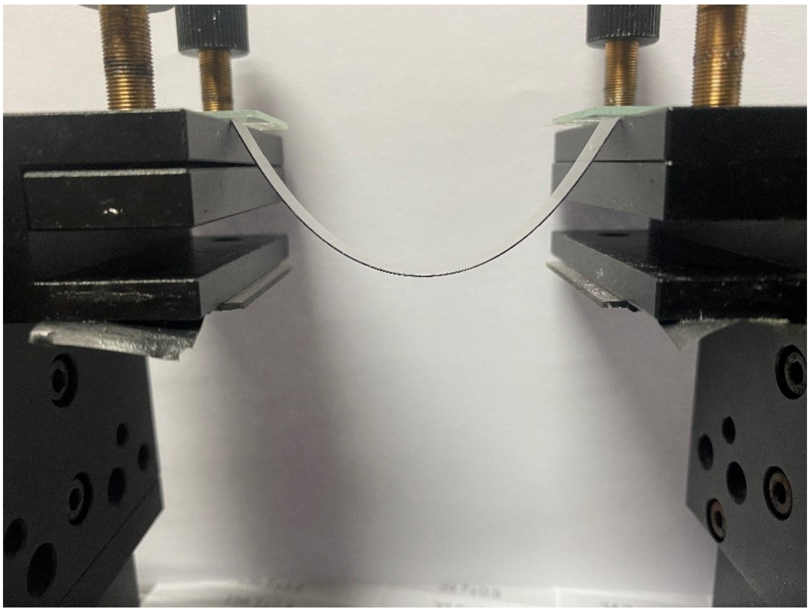

**Figure S10.** Photograph of home-built equipment and mechanically bent electrodes.

## 6. Photoelectron spectroscopy.

Figure S11 shows S 2p and Fe 2p spectra for SAMs. Figure S12 shows the HOMO onset edge and secondary electron peak of the UPS spectra. HOMO is calculated through equation 1 (*hv* = 21.22 eV).

$$E_{HOMO} = E_{cutoff} - E_{onset} - hv \quad (1)$$

Work function (WF) is also calculated from the UPS spectrum and by the following equation:

$$WF = hv - E_{cutoff} \quad (2)$$

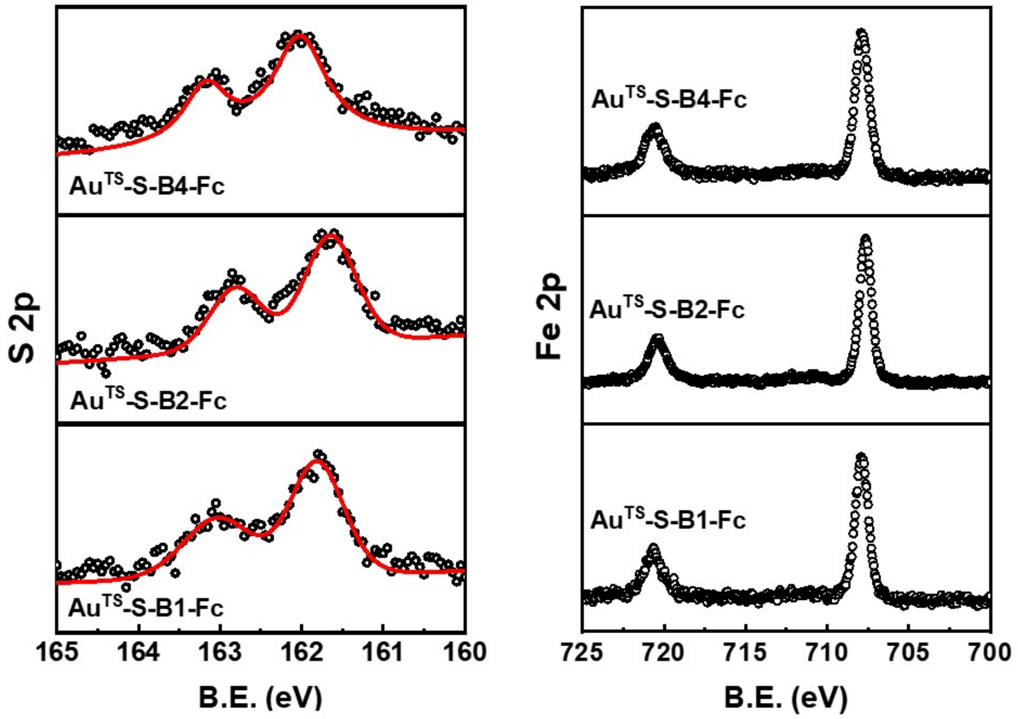


**Figure S11.** S 2p and Fe 2p regions of XPS spectra for SAMs on $Au^{TS}$.

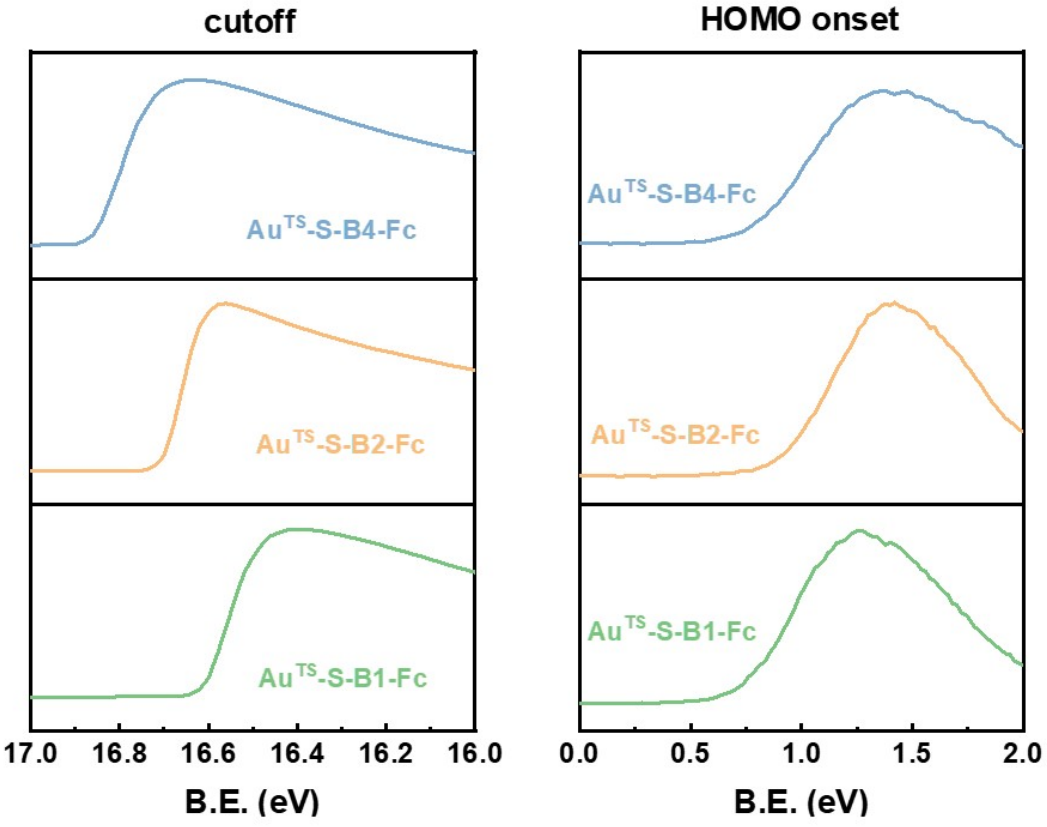


**Figure S12.** Secondary electron cutoff and HOMO onset of UPS spectra.

**Table S3. Summary of HOMO and work function determined from UPS.**

| SAM | HOMO (eV) | WF (eV) |
|---|---|---|
| $Au^{TS}$-B1-Fc | -5.39 | 4.61 |
| $Au^{TS}$-B2-Fc | -5.45 | 4.52 |
| $Au^{TS}$-B4-Fc | -5.17 | 4.36 |

## REFERENCES

1. E. A. Weiss, R. C. Chiechi, G. K. Kaufman, J. K. Kriebel, Z. Li, M. Duati, M. A. Rampi and G. M. Whitesides, Influence of defects on the electrical characteristics of mercury-drop junctions: self-assembled monolayers of n-alkanethiolates on rough and smooth silver, *J. Am. Chem. Soc.*, 2007, **129**, 4336-4349.
2. N. Nerngchamnong, L. Yuan, D. C. Qi, J. Li, D. Thompson and C. A. Nijhuis, The role of van der Waals forces in the performance of molecular diodes, *Nat. Nanotechnol.*, 2013, **8**, 113-118.
3. R. C. Chiechi, E. A. Weiss, M. D. Dickey and G. M. Whitesides, Eutectic gallium-indium (EGaIn): a moldable liquid metal for electrical characterization of self-assembled monolayers, *Angew. Chem. Int. Ed.*, 2008, **47**, 142-144.
4. W. Peng, N. Chen, C. Wang, Y. Xie, S. Qiu, S. Li, L. Zhang and Y. Li, Fine-tuning the molecular design for high-performance molecular diodes based on pyridyl isomers, *Angew. Chem. Int. Ed.*, 2023, **62**, e202307733.
5. W. Du, T. Wang, H.-S. Chu, L. Wu, R. Liu, S. Sun, W. K. Phua, L. Wang, N. Tomczak and C. A. Nijhuis, On-chip molecular electronic plasmon sources based on self-assembled monolayer tunnel junctions, *Nat. Photonics*, 2016, **10**, 274-280.
